# SAV742, a Novel AraC-Family Regulator from *Streptomyces avermitilis*, Controls Avermectin Biosynthesis, Cell Growth and Development

**DOI:** 10.1038/srep36915

**Published:** 2016-11-14

**Authors:** Di Sun, Jianya Zhu, Zhi Chen, Jilun Li, Ying Wen

**Affiliations:** 1 State Key Laboratory of Agrobiotechnology and MOA Key Laboratory of Soil Microbiology, College of Biological Sciences, China Agricultural University, Beijing 100193, China

## Abstract

Avermectins are useful anthelmintic antibiotics produced by *Streptomyces avermitilis*. We demonstrated that a novel AraC-family transcriptional regulator in this species, SAV742, is a global regulator that negatively controls avermectin biosynthesis and cell growth, but positively controls morphological differentiation. Deletion of its gene, *sav_742*, increased avermectin production and dry cell weight, but caused delayed formation of aerial hyphae and spores. SAV742 directly inhibited avermectin production by repressing transcription of *ave* structural genes, and also directly regulated its own gene (*sav_742*) and adjacent gene *sig8 (sav_741*). The precise SAV742-binding site on its own promoter region was determined by DNase I footprinting assay coupled with site-directed DNA mutagenesis, and 5-nt inverted repeats (GCCGA-n_10_/n_12_-TCGGC) were found to be essential for SAV742 binding. Similar 5-nt inverted repeats separated by 3, 10 or 15 nt were found in the promoter regions of target *ave* genes and *sig8*. The SAV742 regulon was predicted based on bioinformatic analysis. Twenty-six new SAV742 targets were identified and experimentally confirmed, including genes involved in primary metabolism, secondary metabolism and development. Our findings indicate that SAV742 plays crucial roles in not only avermectin biosynthesis but also coordination of complex physiological processes in *S. avermitilis*.

Soil-inhabiting species of *Streptomyces* are Gram-positive filamentous bacteria having a complex life cycle that begins with spore germination to form branched multinucleoid vegetative hyphae. The subsequent differentiation process results in formation of erect sporogenic aerial hyphae, followed by chains of unigenomic spores^1^. During the initiation of morphological differentiation, most *Streptomyces* species produce bioactive secondary metabolites, including a variety of many important antibiotics with antimicrobial, antitumor, anthelmintic and immunosuppressive activities^2^,^3^. The processes of morphological differentiation and antibiotic biosynthesis are typically under the tight control of multiple transcriptional regulators that sense and respond to numerous environmental and physiological signals, including nutrient depletion, environmental stress, imbalanced metabolism, growth rate and small signaling molecules such as ppGpp, γ-butyrolactone and c-di-GMP^4^,^5^,^6^,^7^,^8^,^9^. The genes responsible for antibiotic synthesis are typically clustered and under the direct control of cluster-situated regulators (CSRs) that are controlled in turn by various types of higher-level pleiotropic regulators, thus forming a complex regulatory network^5^,^6^,^7^.

Bacterial transcriptional regulators are classified into ~50 families on the basis of sequence alignment and structural and functional criteria^10^. Among the known transcriptional factors in *Streptomyces*, AraC family members participate in the control of genes involved in important biological processes such as carbon source utilization, morphological differentiation, secondary metabolism, pathogenesis and stress responses^10^. The functions of many AraC family members remain unknown. Proteins of this family consist of two domains: a variable N-terminal domain involved in dimerization and ligand binding, and a C-terminal conserved helix-turn-helix (HTH) DNA-binding domain. AdpA from *S. griseus*, the best-studied AraC family transcriptional regulator in the genus, plays a central role in the A-factor regulatory cascade. Transcription of the encoding *adpA* gene is triggered by A-factor, which activates hundreds of target genes that are involved in morphological differentiation and also secondary metabolism^11^,^12^. In the model *S. coelicolor*, AdpA acts as a master regulator that coordinates processes of differentiation and secondary metabolism^13^,^14^. Certain AraC family members besides AdpA are also involved in regulation of secondary metabolism. In *S. hygroscopicus*, RapG is an essential activator of rapamycin biosynthesis by positively regulating transcription of rapamycin polyketide synthase genes *rapA* and *rapB*^15^. NanR4 in *S. nanchangensis* is a repressor of nanchangmycin biosynthesis^16^. Pathogenicity of many plant-pathogenic *Streptomyces* strains depends on synthesis of thaxtomin A, a potent cellulose biosynthesis inhibitor^17^. In the well-studied phytopathogen *S. scabies*, TxtR is required for activating transcription of thaxtomin biosynthetic genes *txtA, txtB* and *txtC*, and plays a crucial role in thaxtomin biosynthesis and virulence^18^.

*S. avermitilis* produces avermectins, a series of 16-membered macrocyclic anthelmintic antibiotics widely utilized in medicine, agriculture and animal husbandry^19^,^20^. Because of the importance of avermectins, many groups have attempted to elucidate the biosynthetic pathway, improve yield, generate new active derivatives and identify regulatory factors^21^,^22^,^23^. However, the use of targeted genetic engineering for construction of avermectin high-producing strains has been limited because the complex regulatory mechanisms of avermectin biosynthesis are poorly understood. *aveR* in avermectin biosynthesis gene cluster encodes a LuxR family cluster-situated activator^24^,^25^. We demonstrated recently that PhoP is a direct repressor of *aveR*^26^, but no other regulators have been shown to control avermectin biosynthesis directly via *aveR*, or via *ave* structural genes. The complete genome sequencing of *S. avermitilis*^27^ revealed 26 encoded AraC family transcriptional regulators, of which only AdpA has been reported to be involved in morphogenesis and melanogenesis by our group^28^. The functions of the other 25 AraC family members remain to be elucidated.

In a search for novel regulators of avermectin biosynthesis, we previously applied a whole-genome chip to compare transcriptomes of *S. avermitilis* wild-type strain and avermectin high-producing strain 76–02-e^29^. We observed that transcription level of *sav_742*, which encodes an AraC-family transcriptional regulator, was greatly downregulated in 76–02-e, suggesting that SAV742 is involved in control of avermectin production. In the present study, we characterized SAV742 as a new global regulator in *S. avermitilis*. SAV742 acts as a direct repressor of avermectin production by directly controlling transcription of several avermectin biosynthetic genes, and also affects cell growth and morphological development. We predicted the SAV742 regulon based on the consensus binding motif of SAV742, and identified new target genes involved in primary metabolism, secondary metabolism and morphological differentiation.

## Results

### SAV742 affects avermectin production, cell growth and morphological differentiation

**Figure 1:**
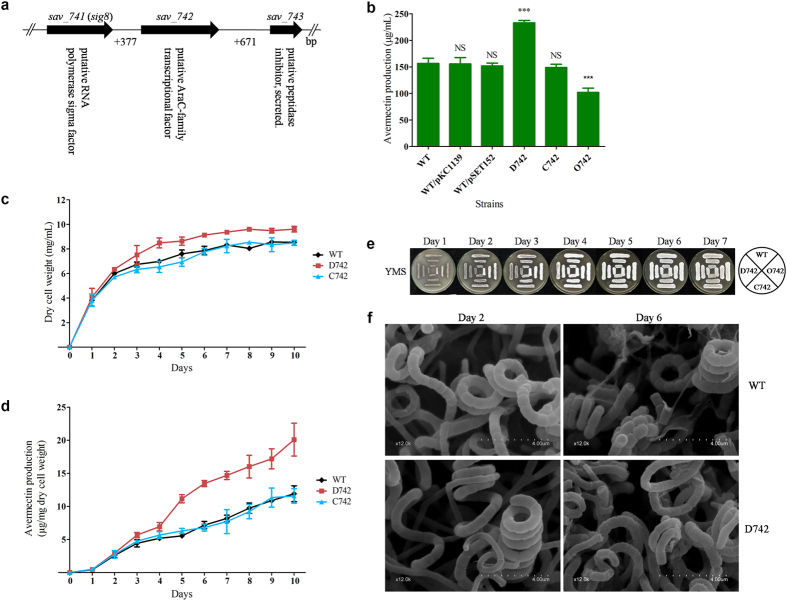
Effects of *sav_742* deletion and overexpression on avermectin production, cell growth and morphological differentiation in *S. avermitilis*. (**a**) Schematic diagram of *sav_742* and its neighboring genes. (**b**) Avermectin yield in WT strain, *sav_742* deletion mutant (D742), complemented strain (C742) and overexpression strain (O742) after fermentation in FM-I for 10 days. Error bar: standard deviation from three replicate experiments. ***P < 0.001; NS, not significant (Student’s t-test). (**c**) Growth curves of WT, D742 and C742 in soluble FM-II. (**d**) Avermectin production per mg dry cell weight of WT, D742 and C742 in soluble FM-II. (**e**) Phenotypes of WT, D742, C742 and O742 grown on YMS agar. (**f**) SEM images showing morphological development of WT and D742 grown on YMS for 2 or 6 days.

The *sav_742* gene, located 0.24 Mb from the *ave* cluster in the left arm of the *S. avermitilis* chromosome, contains 996 nucleotides (nt) and encodes a 331-amino-acid AraC family transcriptional factor that includes an N-terminal ligand-binding domain and a conserved C-terminal helix-turn-helix (HTH) DNA-binding domain homologous to AraC. *sig8 (sav_741*), a convergently transcribed gene located 377 nt upstream of *sav_742*, encodes a sigma70 family alternative sigma factor. *sav_743*, a convergently transcribed gene located 671 nt downstream of *sav_742*, encodes a putative peptidase inhibitor (Fig. 1a). BLAST analysis revealed that SAV742 homologs are distributed widely among *Streptomyces* species, reflecting the important role of this transcriptional regulator in the genus.

To elucidate the function of SAV742 in *S. avermitilis*, we constructed *sav_742* deletion mutant D742 by homologous recombination (see Supplementary Fig. S1). Shake-flask fermentation and HPLC analysis showed that avermectin production of D742 grown in FM-I for 10 days was ~49% higher than that of WT strain ATCC31267 (Fig. 1b). In comparison with WT level, avermectin yield was restored in complemented strain C742, and reduced ~35% in *sav_742* overexpression strain O742. Avermectin contents of vector control strains WT/pSET152 and WT/pKC1139 were nearly the same as that of WT. These findings suggest that SAV742 negatively regulates avermectin production.

To investigate whether avermectin overproduction in D742 was due to changed cell growth, we determined biomass and avermectin yield of WT, D742 and C742 cultured in soluble FM-II^24^. Dry weight and avermectin yield per unit dry weight were higher for D742 than for WT or C742 (Fig. 1c,d), indicating that SAV742 affects both avermectin production and cell growth, and that the increased avermectin yield of D742 resulted at least in part from increased cell growth.

To evaluate the effect of *sav_742* deletion and overexpression on morphological differentiation, spores of strains WT, D742, C742 and O742 were streaked on YMS plates for phenotypic observation. In comparison with WT, D742 showed notably delayed formation of aerial hyphae and spores. The WT phenotype was restored in C742, while O742 displayed earlier differentiation and sporulation (Fig. 1e). Scanning electron microscopy (SEM) was performed to examine the effect of *sav_742* deletion in greater detail. Degree of separation of aerial hyphae was lower in D742 than in WT on day 2, although the two strains showed nearly identical spore size and shape on days 2 and 6 (Fig. 1f); *i. e.*, differentiation was delayed in D742. These findings, taken together, indicate that SAV742 acts as a global regulator to modulate avermectin biosynthesis, cell growth and morphological differentiation in *S. avermitilis*.

### SAV742 is negatively autoregulated

**Figure 2:**
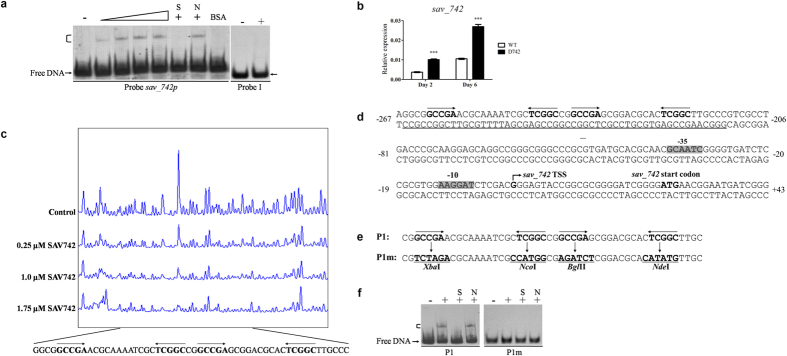
SAV742 directly represses its own gene. (**a**) EMSAs of the interaction of His_6_-SAV742 with its own promoter region. 0.15 nM labeled probe was added to each reaction mixture. For specific (lane S) or nonspecific (lane N) competition assays, a 300-fold excess of unlabeled competitor DNA was used. 0.1% BSA and labeled nonspecific probe I were used as negative protein and probe controls, respectively. Lanes –: EMSAs without His_6_-SAV742. Lanes 2 to 5 contained 125, 250, 375, and 500 nM His_6_-SAV742, respectively. 500 nM His_6_-SAV742 was used for competition assays and probe I (Lanes +). Arrows: free probes. Bracket: SAV742-DNA complex. (**b**) qRT-PCR analysis of *sav_742* transcription level in WT and D742 grown in FM-I for 2 or 6 days. Relative values were obtained using 16 S *rRNA* as internal reference. *sav_742*: 81-bp transcript amplified from the remaining *sav_742* ORF in D742 with primers SD66A and SD66B. ***P < 0.001 (Student’s t-test). (**c**) Determination of SAV742-binding sites on its own promoter region by DNase I footprinting assay. Top fluorogram: control reaction without protein. Protection regions were acquired with increasing concentrations (0.25, 1.0, 1.75 μM) of His_6_-SAV742 protein. (**d**) Nucleotide sequences of *sav_742* promoter region and SAV742-binding sites. Numbers indicate distance (nt) from *sav_742* TSS. Bent arrow: *sav_742* TSS. Straight arrows: inverted repeats. Shaded areas: putative -10 and -35 regions. Underlining: SAV742-binding site. (**e**) Mutational analysis of SAV742-binding sites. Each probe was 50-bp. Mutations were introduced into WT probe P1 to generate mutated probe P1m. Underlining: altered nucleotides. (**f**) EMSAs using WT probe P1 and mutated probe P1m. Each lane contained 0.15 nM labeled probe. A 300-fold excess of unlabeled competitor probe was used in competition assays. His_6_-SAV742 concentration: 500 nM.

To identify SAV742 target genes, we performed electrophoretic mobility shift assays (EMSAs) using soluble His_6_-SAV742 that was overexpressed in and purified from *Escherichia coli*. AraC family transcriptional factors are generally autoregulated^30^,^31^,^32^. To determine whether SAV742 directly regulates its own gene, we labeled the promoter region of *sav_742* as probe *sav_742p* and used it for EMSAs. His_6_-SAV742 did not bind to negative control probe I, but bound specifically to probe *sav_742p* and generated a clearly shifted band (Fig. 2a). Binding specificity was examined by competitive assays with a 300-fold excess of unlabeled specific probe *sav_742p* (lane S), which abolished binding of SAV742 to labeled probe *sav_742p*, or of nonspecific probe I (lane N), which did not reduce the retarded signal. BSA was used as negative protein control.

To investigate the effect of *sav_742* deletion on the expression of its own gene, *sav_742* transcription level in WT and D742 grown in FM-I was analyzed by quantitative real-time RT-PCR (qRT-PCR). *sav_742* transcription level was higher in D742 than in WT on days 2 and 6 (Fig. 2b), indicating that SAV742 represses its own transcription.

To clarify the mechanism whereby SAV742 regulates its own gene, we determined the *sav_742* TSS by 5′ rapid amplification of cDNA ends (5′-RACE). The TSS was localized to G (see Supplementary Fig. S2), 24 nt upstream of the *sav_742* translational start codon. Subsequent DNase I footprinting assays revealed that SAV742 protected a 54-nt region on the *sav_742* promoter region (Fig. 2c). The binding sequence of SAV742 is located far upstream of the *sav_742* TSS, extending from positions −266 to −213 nt relative to the TSS (Fig. 2d). Analogously, ScbR2 was shown to directly repress *kasO*^33^. The mechanism of such transcriptional repression remains to be clarified. Perhaps SAV742 molecules on the binding site produce a higher-order DNA-protein structure that prevents RNA polymerase binding to the *sav_742* promoter, or recruit other repressors.

AraC family transcriptional factors generally form symmetric dimers and bind to palindromic sequences^30^,^31^,^32^. DNAMAN analysis of the SAV742-binding site revealed two perfect palindromic sequences, both including identical 5-nt inverted repeats separated by 10 or 12 nt (GCCGA -n_10_/n_12_-TCGGC) (Fig. 2c,d). To test the importance of the palindromic sequences in SAV742 binding, site-directed mutagenesis of WT probe P1 (within the binding site) was performed on the inverted repeats (Fig. 2e). Binding activities of SAV742 with WT probe P1 or mutated probe P1m (lacking inverted repeats) were determined by EMSAs. In contrast to P1, P1m showed no binding to SAV742 (Fig. 2f). These findings indicate that the 5-nt inverted repeats are essential for SAV742 binding. Such 5-nt inverted repeats separated by 15 nt (GCCGACCAAAGTGTCTTTGGTCGGC) were found on the promoter region of the gene adjacent to *sav_742, sig8 (sav_741*). Results of EMSAs and qRT-PCR showed that SAV742 directly activates *sig8* expression (see Supplementary Fig. S3a,b), indicating that spacer sequence and spacer length between the 5-nt inverted repeats are not important for SAV742 binding.

### SAV742 directly represses *ave* structural genes

**Figure 3:**
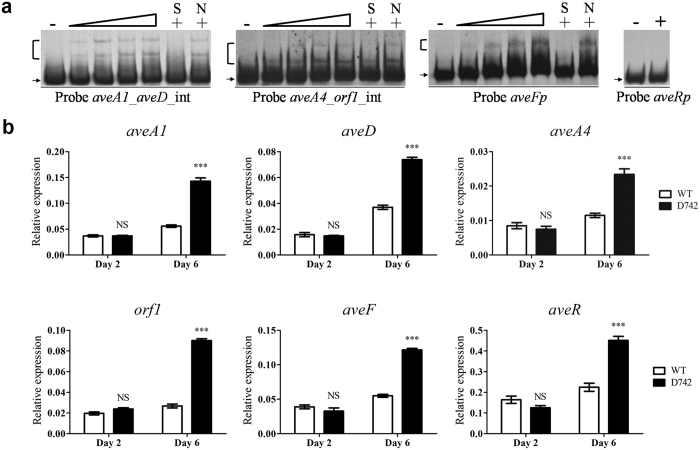
SAV742 directly represses transcription of *ave* structural genes. (**a**) EMSAs of SAV742 binding to intergenic regions of *aveA1*-*aveD* and *aveA4-orf1*, and the *aveF* promoter region. EMSA conditions as described for Fig. 2a. (**b**) qRT-PCR analysis of *aveA1, aveD, aveA4, orf1, aveF* and *aveR* in WT and D742 grown in FM-I. ***P < 0.001; NS, not significant (Student’s t-test).

Transcriptional factors generally bind to similar DNA motifs in the regulatory regions of target genes. EMSAs and footprinting assays revealed the presence of 5-nt inverted repeats that play an important role in SAV742 binding. Similar imperfect 5-nt inverted repeats separated by 3 or 10 nt were present in several promoter regions of the *ave* gene cluster: the bidirectional promoter regions of *aveA1*-*aveD* (GCCGAGCATTGCC) and *aveA4-orf1* (GCCGATCCGAGAGCGCAGGC), and the promoter region of *aveF* (ACCGCTAGGCAATGCTCGGC). The ability of SAV742 to bind to these promoter regions was evaluated by EMSAs. Purified His_6_-SAV742 bound specifically to the intergenic regions of *aveA1*-*aveD* (probe *aveA1*_*aveD_*int) and *aveA4-orf1* (probe *aveA4_orf1*_int), and the promoter region of *aveF* (probe *aveFp*) (Fig. 3a). It did not bind to the promoter region of cluster-situated activator gene *aveR* (probe *aveRp*), which is essential for activating transcription of *ave* structural genes. These findings suggest that SAV742 regulates avermectin production through direct control of *ave* structural genes, rather than *aveR*.

qRT-PCR analysis was performed to assess the effect of SAV742 on expression of target *ave* structural genes. Transcription levels of *aveA1, aveD, aveA4, orf1* and *aveF* did not differ significantly between D742 and WT on day 2, but were higher in D742 on day 6 (Fig. 3b), consistent with the increased avermectin production in D742. Thus, SAV742 appears to directly repress transcription of target *ave* structural genes primarily in the mid-to-late stage of fermentation. *aveR* transcription level was also increased in D742 on day 6 (Fig. 3b), contributing to increased avermectin production. However, the repressing effect of SAV742 on *aveR* was indirect.

### Prediction of the SAV742 regulon

Analysis of 5-nt inverted repeats in the five SAV742-binding promoter regions mentioned above (*sav_742p, sig8p, aveA1*_*aveD_*int, *aveA4_orf1*_int, *aveFp*) revealed a consensus sequence RCCGM- n_3_/n_10_/n_12_/n_15_-YHGSC (R = A/G; M = A/C; Y = C/T; H = A/C/T; S = G/C). Because spacer length is not important for SAV742 binding, we used palindromic sequence RCCGM- n_0–15_-YHGSC to scan the *S. avermitilis* genome, using the RSAT (Regulatory Sequence Analysis Tools) web-based application (http://embnet.ccg.unam.mx/rsa-tools/) to search for new putative SAV742 target genes. A total of 3278 putative SAV742 target genes were identified, of which over half (1747) have unknown function or are unclassified (see Supplementary Table S1). The remaining 1531 putative targets are listed in Supplementary Table S1 and include the confirmed SAV742 targets (*sav_742, sig8, aveA1, aveD, aveA4, orf1, aveF*), as expected. Among the 1531 genes, 350 are associated with regulatory functions, and the rest were assigned to 17 groups based on the KEGG *S. avermitilis* pathways database (www.genome.jp/kegg-bin/show_organism?org=sma) (see Supplementary Table S1), reflecting the extent and complexity of the SAV742 regulatory network.

### Targets of SAV742 involved in secondary metabolism and development

**Figure 4:**
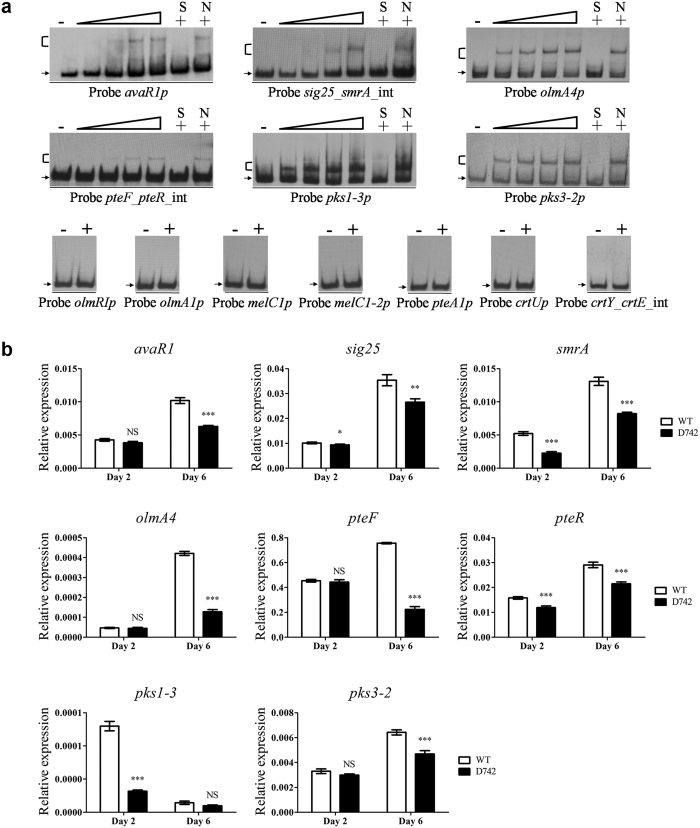
Predicted new target genes involved in secondary metabolism. (**a**) EMSAs of His_6_-SAV742 with promoter regions of 16 predicted target genes. (**b**) qRT-PCR analysis of 8 newly identified SAV742 target genes in WT and D742. *P < 0.05; **P < 0.01; ***P < 0.001; NS, not significant (Student’s t-test).

The 84 predicted targets of SAV742 are involved in secondary metabolism, including the confirmed five target *ave* genes for avermectin biosynthesis (see Supplementary Table S1). Among 350 putative SAV742 targets having regulatory function, three have been reported to negatively regulate avermectin production: *avaR1 (sav_3705*, encoding a putative gamma-butyrolactone receptor protein)^34^,^35^, *sig25 (sav_3351*, encoding an ECF sigma factor) and *smrA (sav_3352*, encoding a two-component system response regulator)^36^. EMSAs showed that SAV742 bound specifically to the *avaR1* promoter region and *sig25-smrA* bidirectional promoter region (Fig. 4a). Transcription levels of *sig25, smrA* and *avaR1* were all downregulated in D742 (Fig. 4b), consistent with the increased avermectin yield in this mutant. These findings indicate that SAV742 regulates avermectin production both directly and via regulatory cascades.

Some putative SAV742 target genes associated with biosynthesis of other secondary metabolites were selected for EMSA evaluation, including genes involved in oligomycin biosynthesis: *olmRI (sav_2902*, encoding a LuxR-family pathway-specific activator)^37^, *olmA1 (sav_2899*, encoding a modular polyketide synthase) and *olmA4 (sav_2892*, encoding a modular polyketide synthase); in melanin biosynthesis: *melC1 (sav_1136*) and *melC1-2 (sav_5361*), both encoding tyrosinase co-factor proteins; in filipin biosynthesis: *pteF (sav_409*, encoding a LuxR-family pathway-specific activator)^38^, *pteR (sav_410*, encoding a DnrI/RedD/AfsR-family putative pathway-specific activator)^38^ and *pteA1 (sav_419*, encoding a modular polyketide synthase); in lycopene biosynthesis: *crtU (sav_1019*, encoding a beta-carotene desaturase/methylase), *crtY (sav_1021*, encoding a lycopene cyclase) and *crtE (sav_1022*, encoding a geranylgeranyl diphosphate synthase); in biosynthesis of unknown secondary metabolites: *pks1-3 (sav_7362*) and *pks3-2 (sav_2281*), encoding putative modular polyketide synthases that belong respectively to the *pks1* and *pks3* cluster. The EMSA results showed that SAV742 bound to the promoter regions of *olmA4, pks1-3, pks3-2* and *pteF*-*pteR* (bidirectional), but not to those of *olmRI, olmA1, melC1, melC1-2, pteA1, crtU* or *crtY*-*crtE* (bidirectional) (Fig. 4a). Transcription levels of *olmA4, pteF, pteR, pks1-3* and *pks3-2* were all reduced in D742 (Fig. 4b), indicating that SAV742 acts as an activator of these newly identified target genes related to secondary metabolism.

**Figure 5:**
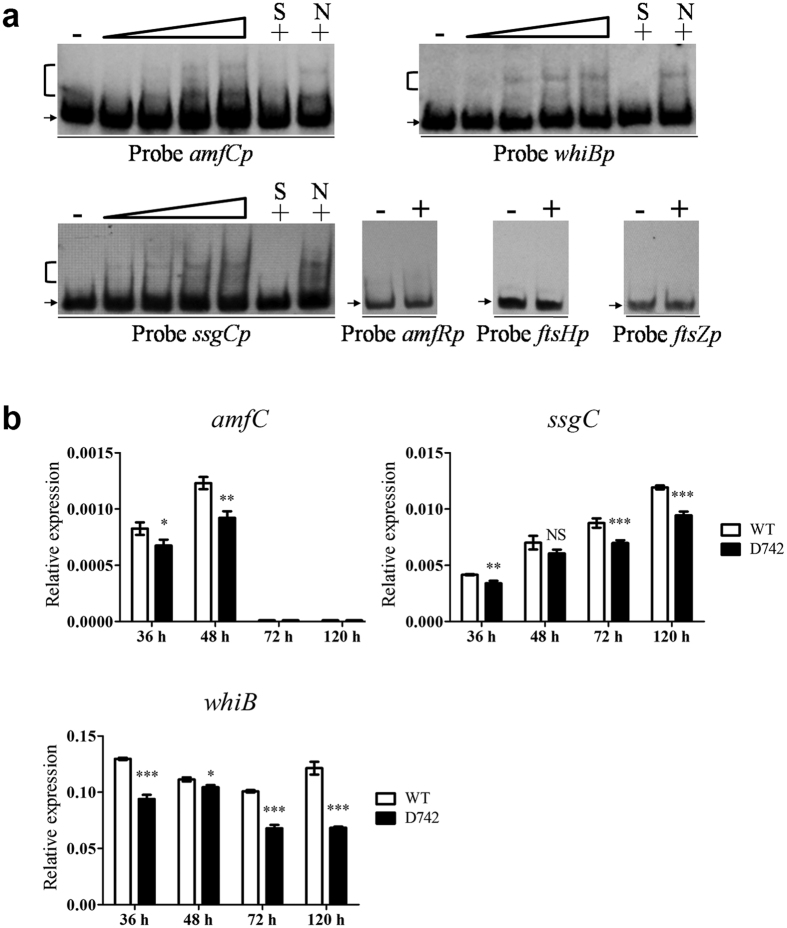
Predicted new target genes involved in morphological differentiation. (**a**) EMSAs of His_6_-SAV742 with promoter regions of *amfC, whiB, ssgC, amfR, ftsH* and *ftsZ*. (**b**) qRT-PCR analysis of newly identified SAV742 target genes *amfC, whiB* and *ssgC* in WT and D742. Statistical notations as in Fig. 4.

Our phenotypic observations showed that SAV742 affects morphological differentiation. We therefore performed EMSAs on several putative SAV742 targets involved in morphological differentiation: *amfC (sav_4026*, encoding an aerial mycelium-associated protein)^39^, *amfR (sav_7499*, encoding a two-component system response regulator essential for aerial hyphae formation)^40^, *whiB (sav_5042*, encoding a WhiB-family transcriptional regulator essential for sporulation)^41^, *ssgC (sav_6810*, encoding a putative cell division protein homologous to *ssgB* in *S. coelicolor*)^42^, *ftsH (sav_4666*, encoding a putative cell division protein) and *ftsZ (sav_6124*, encoding a putative cell division GTPase). The results showed that SAV742 bound to the promoter regions of *amfC, whiB* and *ssgC*, but not to those of *amfR, ftsH* or *ftsZ* (Fig. 5a). Transcription levels of *amfC, whiB* and *ssgC* were determined by qRT-PCR using RNAs extracted from WT and D742 grown on YMS plates for 36 h (vegetative growth stage), 48 h (aerial growth stage), 72 h (early stage of sporulation) or 120 h (spore maturation stage). Transcription levels of *whiB* and *ssgC* were lower in D742 than in WT at various time points (Fig. 5b). *amfC* expression level was lower in D742 than in WT at 36 h and 48 h, and was zero at 72 h and 120 h in both strains. These findings are consistent with the delayed differentiation phenotype of D742, suggesting that SAV742 positively regulates morphological differentiation by directly activating transcription of *amfC, whiB* and *ssgC*.

### Targets of SAV742 involved in primary metabolism

Many genes involved in primary metabolism were predicted to be targets of SAV742 (see Supplementary Table S1). We selected representative candidate genes involved in carbon, nitrogen or energy metabolism for evaluation. These candidates included genes involved in the tricarboxylic acid (TCA) cycle: *aceE1 (sav_5800*, encoding a putative pyruvate dehydrogenase E1 component), *acnA (sav_2258*, encoding a putative aconitase), *korA (sav_4877*, encoding the alpha subunit of 2-oxoglutarate ferredoxin oxidoreductase) and *icdA (sav_7214*, encoding a putative isocitrate dehydrogenase); in glycolysis: *pgmA (sav_803*, encoding a putative phosphoglucomutase), *gap2 (sav_6296*, encoding a putative glyceraldehyde-3-phosphate dehydrogenase) and *pykA1 (sav_2825*, encoding a putative pyruvate kinase); in N-acetylglucosamine (GlcNAc) utilization: *dasR (sav_3023*, encoding a putative GntR-family transcriptional regulator)^43^; in fatty acid biosynthesis and degradation: *accA3 (sav_3337*, encoding the alpha subunit of acyl-CoA carboxylase), *accD4 (sav_3331*, encoding the beta subunit of acyl-CoA carboxylase), *fadD5 (sav_1258*, encoding a putative acyl-CoA synthetase/long chain fatty acid: CoA ligase), *fadA6 (sav_7026*, encoding a putative 3-ketoacyl-CoA thiolase/acetyl-CoA acetyltransferase) and *pcaF1 (sav_1604*, encoding a putative beta-ketoadipyl-CoA thiolase); in glutamine biosynthesis: *glnA1 (sav_5954*, encoding a putative glutamine synthetase); in valine, leucine and isoleucine biosynthesis: *leuA2 (sav_5601*, encoding a putative 2-isopropylmalate synthase), *leuC (sav_2686*, encoding the large subunit of 3-isopropylmalate dehydratase), *ilvB1 (sav_2733*, encoding the large subunit of acetolactate synthase), *ilvC (sav_2731*, encoding a putative ketol-acid reductoisomerase), *ilvD1 (sav_4716*, encoding a putative dihydroxy-acid dehydratase) and *ilvE (sav_2717*, encoding a putative branched-chain amino acid aminotransferase); in oxidative phosphorylation: *ndh1 (sav_1892*, encoding a putative NADH dehydrogenase) and *ctaD2 (sav_6537*, encoding the subunit I of cytochrome c oxidase).

**Figure 6:**
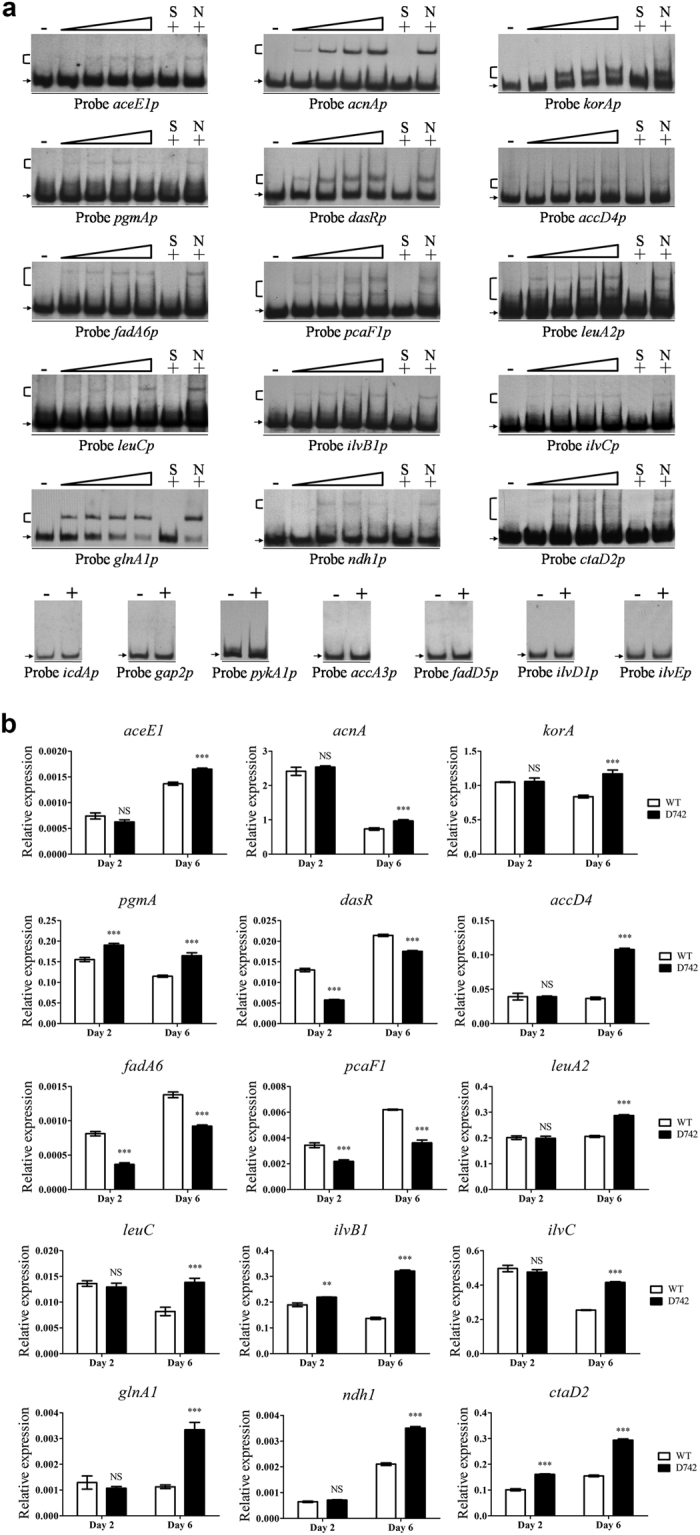
Predicted new target genes involved in primary metabolism. (**a**) EMSAs of His_6_-SAV742 with promoter regions of 22 predicted target genes. (**b**) qRT-PCR analysis of 15 newly identified target genes of SAV742. Statistical notations as in Fig. 4.

EMSA results demonstrated specific binding of SAV742 to the promoter regions of *aceE1, acnA, korA, pgmA, dasR, accD4, fadA6, pcaF1, leuA2, leuC, ilvB1, ilvC, glnA1, ndh1* and *ctaD2*, but not to those of *icdA, gap2, pykA1, accA3, fadD5, ilvD1* or *ilvE* (Fig. 6a). qRT-PCR analysis was performed to evaluate the regulatory role of SAV742 in expression of these newly identified target genes. SAV742 was found to repress transcription of *aceE1, acnA, korA, pgmA, accD4, leuA2, leuC, ilvB1, ilvC, glnA1, ndh1* and *ctaD2; i.e.*, transcription levels of these genes were higher in D742 than in WT on day 6 or on days 2 and 6 (Fig. 6b). In contrast, transcription levels of *dasR, fadA6* and *pcaF1* were lower in D742 than in WT (Fig. 6b). These findings indicate that SAV742 has a dual repressor/activator function and plays a pleiotropic role in primary metabolism.

## Discussion

The present study clarified the molecular mechanism underlying SAV742 function in avermectin biosynthesis, and identified the SAV742 regulon in *S. avermitilis*. SAV742 clearly functions as a global regulator of avermectin production, cell growth and morphological development in this species. EMSA and qRT-PCR results indicate that SAV742 plays a direct role in repressing avermectin production, which is mediated by *ave* structural genes, but not by the CSR gene *aveR*. AveR is the only CSR of the *ave* gene cluster essential for activating transcription of all *ave s*tructural genes^24^,^25^. Among transcriptional regulators located outside the *ave* gene cluster in *S. avermitilis*, SAV742 is the first shown to directly regulate *ave* structural genes, reflecting the subtlety and complexity of avermectin biosynthesis regulation. It is generally uncommon for global/pleiotropic regulators to directly control structural genes involved in antibiotic biosynthesis; however, similar phenomena have been observed in other *Streptomyces* species. For instance, WhiG from *S. chattanoogensis* L10, an industrial natamycin producer, controls natamycin production as well as spore development^44^. It directly activates natamycin production by binding to the promoters of biosynthetic genes *scnC* and *scnD*. In *S. roseosporus*, the pleiotropic regulator AtrA is essential for both daptomycin production and development^45^. AtrA directly stimulates daptomycin production by interacting with the promoter of biosynthetic gene *dptE*. In *S. coelicolor*, the two-component system AfsQ1/Q2 is involved in regulation of primary metabolism, secondary metabolism and morphological differentiation^46^. The response regulator AfsQ1 positively regulates production of yellow-pigmented coelimycin P2 by directly activating expression of the *cpkABC* operon, which encodes three large PKSs for coelimycin biosynthesis^47^. Direct regulatory mechanisms of antibiotic biosynthesis mediated by structural genes rather than CSR genes, as in these cases, may facilitate precise and rapid regulation of specific biosynthetic genes in response to intracellular and/or extracellular signals. Our observations that SAV742 indirectly represses *aveR* expression, directly activates expression of *sig25, smrA* and *avaR1*, which are involved in indirect inhibition of avermectin production, suggest that the effect of SAV742 on avermectin production is also exerted in a cascade manner. In addition to controlling avermectin biosynthesis, SAV742 also regulates oligomycin, filipin and unknown metabolites by activating transcription of structural genes (*olmA4, pks1-3, pks3-2*) or CSR genes (*pteF, pteR*), indicating a pleiotropic role of SAV742 in secondary metabolism.

We identified 15 new SAV742 target genes involved in primary metabolism. Among these, one gene encoding an enzyme in glycolysis (*pgmA*), and three genes encoding enzymes in the TCA cycle (*aceE1, acnA* and *korA*), were found to be upregulated in D742, suggesting an acceleration of central metabolism that in turn promoted cell growth and added precursors and energy for avermectin biosynthesis. *ndh1* and *ctaD2* encode key enzymes in oxidative phosphorylation and energy generation. Enhanced expression of these two genes in D742 may promote both avermectin production and cell growth by increasing energy availability. *glnA1* encodes glutamine synthetase, which plays a key role in nitrogen assimilation. Enhanced expression of this gene in D742 may accelerate primary metabolism and increase cell growth. The starter units 2-methylbutyryl-CoA (“a” components) and isobutyryl-CoA (“b” components) of avermectin biosynthesis are derived from isoleucine and valine, respectively^48^. Transcription of *leuA2, leuC, ilvB1* and *ilvC* (which are involved in isoleucine and valine biosynthesis) was elevated in D742, thus providing more precursors for avermectin production. Elongation of the avermectin polyketide chain requires addition of seven malonyl-CoA units and five methylmalonyl-CoA units to the starter units^48^. *accD4* encodes the beta subunit of acyl-CoA carboxylase, which is essential for conversion of acetyl-CoA to malonyl-CoA. Increased transcription of *accD4* in D742 may thus direct more acetyl-CoA toward malonyl-CoA conversion for avermectin biosynthesis. *fadA6* and *pcaF1* are involved in fatty acid degradation, which can yield acetyl-CoA. The reduced transcription of these two genes and enhanced glycolysis observed in D742 suggest that the acetyl-CoA pool in this mutant is generated mainly by central metabolism, not by fatty acid degradation. The GntR-family regulator DasR is involved in regulation of metabolism and transport of GlcNAc, a preferred carbon and nitrogen source for *Streptomyces*^6^. The direct control of *dasR* by SAV742 suggests that SAV742 is involved in regulation of nutrition utilization. DasR also regulates antibiotic synthesis in *S. coelicolor*^6^. The regulatory role of DasR in avermectin biosynthesis remains to be investigated.

We showed that SAV742 plays a positive role in morphological differentiation, and identified three development-associated genes (*amfC, whiB, ssgC*) as SAV742 targets. *amfC* encodes a protein required for mycelium formation in *S. coelicolor* and *S. griseus*^39^*. whiB* expression is required for the initiation stage of sporulation septation in *S. coelicolor*^41^,^49^. The *ssgC* homologue gene (*ssgB*) in *S. coelicolor* is also essential for initiation of sporulation^42^. Reduced expression of *amfC, whiB* and *ssgC* in D742 may thus account for the delayed formation of aerial hyphae and spores. However, at this stage we cannot rule out the possibility that other putative development-related target genes of SAV742 contribute to the phenotype observed in D742. This possibility awaits further investigation.

**Figure 7:**
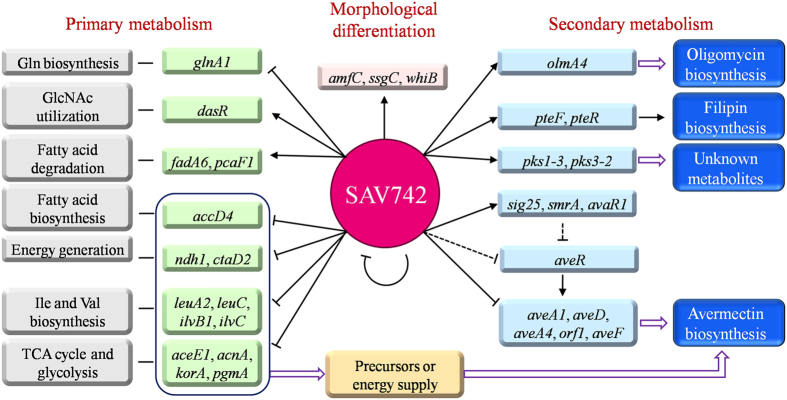
Proposed model of SAV742-mediated regulatory network in *S. avermitilis*. SAV742 exerts pleiotropic regulatory effects on primary metabolism, secondary metabolism and morphological development, and interacts with regulatory genes such as *dasR, sig25, smrA* and *avaR1*. Solid arrows: activation. Bars: repression. Solid lines: direct control. Dashed lines: indirect control. Hollow arrows: production of energy or small molecules.

Based on the present findings, we propose a model of the SAV742-mediated regulatory network involved in primary metabolism, secondary metabolism and morphological development (Fig. 7). In this model, SAV742 exerts its negative regulatory effect on avermectin production through at least four pathways: (i) directly repressing transcription of *ave* structural genes; (ii) indirectly repressing expression of CSR gene *aveR* through a yet-unknown mechanism; (iii) directly controlling regulatory genes (*sig25, smrA, avaR1*) related to avermectin biosynthesis; (iv) directly regulating genes involved in primary metabolism, thereby controlling energy supply and precursor pools for avermectin biosynthesis, in addition to cell growth. SAV742 has been shown to control other secondary metabolites, such as oligomycin and filipin, by interacting with the promoter regions of structural genes or CSR genes. In regard to morphological development, SAV742 directly activates expression of *amfC, whiB* and *ssgC*, thereby affecting aerial hyphae formation and sporulation. Certain primary metabolism genes involved in carbon, nitrogen and energy metabolism are also under the direct control of SAV742, suggesting that SAV742 plays a crucial key role in the overall co-ordination of *S. avermitilis* metabolism.

*sig8*, the homologue gene *sigB* in *S. coelicolor* is required for osmoprotection^50^, was identified as a SAV742 target. Other stress response genes were also predicted SAV742 targets. The *sav_348* homologue *katB* encodes a catalase that protects *S. coelicolor* from osmotic stress^51^. *sig29 (sav_3490*) is the homologue of *S. coelicolor sigI*, whose expression is induced by osmotic stress^52^. *osaA*/*osaB (sav_2512*/*sav_2511*) encodes a two-component system involved in osmotic stress adaptation^53^. *sig22 (sav_3038*) encodes an ECF sigma factor similar to *S. coelicolor* SigR, which controls the response to thiol-oxidative stress^54^. *lexA (sav_2463*) encodes a putative SOS regulatory protein. *cspB1 (sav_3907*), *cspB2 (sav_3932*), *cspD1 (sav_826*), *cspD2 (sav_893*), *cspD3 (sav_4154*), *cspD4 (sav_4447*) and *cspD5 (sav_4776*) encode putative cold shock proteins. *htpG (sav_2672*) and *htpX2 (sav_6003*) encode putative heat shock proteins. These previous findings suggest that SAV742 plays a role in eliciting “survival responses” for adaptation to stress signals. This possibility remains to be investigated.

The identified SAV742 targets contain consensus 5-nt inverted repeats with intervals of 0, 1 to 15 nt (see Supplementary Table S1). The DNA-binding specificity of SAV742 is therefore very low. The predicted SAV742 regulon contains >3000 genes, which have been extensively catalogued and are distributed widely across the *S. avermitilis* chromosome. It is unlikely that SAV742 binds to all the predicted targets; however, our findings strongly indicate that SAV742 is an important global regulator in *S. avermitilis* that coordinates multiple physiological processes for adaptation to complex and changing environments. The DNA-binding characteristic of SAV742 is analogous to that of another AraC-family regulator, AdpA, which also functions as a global regulator of secondary metabolism and development in *Streptomyces*^11^,^12^,^13^,^14^. AdpA binds to >1200 sites and directly controls >500 genes in *S. griseus*^12^. Analysis of the crystal structure of the AdpA DNA-binding domain in *S. griseus* provided an explanation of the tolerant DNA-binding specificity of AdpA^55^. The DNA-binding domains of SAV742 and AdpA have structural similarities (see Supplementary Fig. S4), which may account for the tolerant DNA-binding specificity of SAV742. The low DNA-binding specificity of SAV742 and AdpA allows them to bind to many sites on the chromosome, which may be advantageous in terms of the ability to alter expression profiles of many genes within a short period. Further studies utilizing high-throughput technologies (*e.g.,* ChIP-seq) are underway to precisely identify the SAV742 targets, and are expected to clarify the broader roles and biological significance of SAV742 in *S. avermitilis*.

## Methods

### Plasmids, strains and growth conditions

Plasmids and bacterial strains used in this study are listed in Supplementary Table S2. *S. avermitilis* ATCC31267, the wild-type (WT) strain, was used as parent strain for gene disruption and propagation. ATCC31267 and its derivatives were grown at 28 °C on YMS agar for sporulation^56^, or in YEME liquid medium^57^ containing 25% sucrose for mycelia growth. Media and culture conditions for avermectin production and protoplast regeneration were as described in our previous reports^58^,^59^. *E. coli* strains were grown at 37 °C in standard LB medium. *E. coli* JM109 was used as cloning host for propagating plasmids. *E. coli* BL21 (DE3) was used to overexpress SAV742 protein. *E. coli* ET12567 (*dam dcm hsdS*)^60^ was used for propagating non-methylated plasmids for introduction into *S. avermitilis*. When necessary, antibiotics were added as described previously^28^.

### Gene deletion, complementation, and overexpression

To construct a *sav_742* deletion mutant, two homologous arms flanking *sav_742* were prepared by PCR from WT genomic DNA. A 551-bp 5′-flanking region (positions −476 to +75 relative to the *sav_742* start codon) was amplified with primers SD4A and SD4B, and a 545-bp 3′-flanking region (positions +978 to +1522) was amplified with primers SD5A and SD5B. The two PCR fragments were digested with *Hin*dIII/*Xba*I and *Xba*I/*Eco*RI, respectively, and then simultaneously ligated into *Hin*dIII/*Eco*RI-digested pKC1139 vector to generate *sav_742* deletion vector pKCD742. Non-methylated pKCD742 was introduced into WT protoplasts. Double-crossover recombinant strains were selected as described previously^28^, and the resulting *sav_742* deletion mutants were analyzed and confirmed by PCR using primer pairs SD24A/SD24B (flanking the exchange regions) and SD35A/SD35B (located within the deletion region of *sav_742*) (see Supplementary Fig. S1). When primers SD24A and SD24B were used, a 1.4-kb band appeared, whereas a 2.2-kb band was detected from WT genomic DNA. When primers SD35A and SD35B were used, only WT DNA produced a 524-bp band, as predicted. We thus obtained *sav_742* gene deletion mutant D742, in which the coding region of *sav_742* was mostly deleted.

For complementation of D742, a 1606-bp DNA fragment containing the promoter and ORF of *sav_742* was amplified from WT genomic DNA with primers SD22D and SD22B. The PCR product was digested with *Xba*I/*Hin*dIII and cloned into the integrative vector pSET152 at the corresponding sites to give *sav_742*-complemented vector pSET152–742, which was transformed into D742 to obtain complemented strain C742.

For overexpression of *sav_742*, a 1278-bp fragment carrying *sav_742* ORF was amplified from WT genomic DNA by PCR using primers SD22C and SD22B. The PCR product was digested with *Xba*I/*Hin*dIII, and then inserted into the corresponding sites of pJL117 to generate pJL117–742. The 1.6-kb *Eco*RI/*Hin*dIII fragment containing *sav_742* ORF and *Streptomyces* strong constitutive promoter ermE*p from pJL117–742 was ligated into pKC1139 to generate *sav_742* overexpression vector pKC1139-erm-742, which was introduced into WT strain to obtain *sav_742* overexpression strain O742.

The primers used in this study are listed in Supplementary Table S3.

### Overexpression and purification of His_6_-SAV742

The 1232-bp *sav_742* coding region (331 amino acids) was amplified by PCR from WT genomic DNA using primers SD22A and SD22B. The obtained PCR product was cut with *Bam*HI/*Hin*dIII and ligated into expression vector pET-28a (+) to generate pET-742, which was then transformed into *E. coli* BL21 (DE3) for overexpression of N-terminal His_6_-tagged SAV742 recombinant protein. Following induction by 0.2 mM IPTG, bacteria containing His_6_-SAV742 were collected, washed, resuspended in a lysis buffer^36^, and sonicated on ice. Soluble His_6_-SAV742 was purified by Ni-NTA agarose chromatography (Qiagen) according to the manufacturer’s instructions. The purified protein was quantified by Quick Start Bradford Dye Reagent (Bio-Rad) and stored at −80 °C until use.

### EMSAs

EMSAs were performed using a digoxigenin (DIG) gel shift kit (Roche, 2^nd^ Generation) as described previously^58^. DNA probes were generated by PCR using primers as listed in Supplementary Table S3, and labeled with DIG at their 3′ ends. Each reaction mixture (20 μL) contained 1 μg poly [d(I-C)] (vial 9), 0.15 nM labeled probe, and various quantities of His_6_-SAV742 in binding buffer (vial 5), and was incubated for 30 min at 25 °C. Samples were loaded on 5% (w/v) native polyacrylamide gels, subjected to electrophoresis, and DNAs in the gels were transferred onto a nylon membrane (Roche). Signals of labeled DNAs on X-ray film were recorded by chemiluminescence detection. Binding specificity between His_6_-SAV742 and DNA probes was analyzed by addition of appropriate 300-fold specific or non-specific unlabeled probes to the reaction mixture before incubation.

### DNase I footprinting

A fluorescent labeling procedure^61^ was used for these assays. To determine the binding site of SAV742 in the promoter region of its own gene, a 524-bp 5′ FAM-labeled fragment corresponding to the upstream region of *sav_742* was amplified by PCR using primers FAM-SD169A/SD169C, followed by purification from agarose gel. Labeled probe (400 ng) and various concentrations of His_6_-SAV742 were mixed in the binding buffer, and incubated for 30 min at 25 °C. DNase I (0.016 U; TaKaRa) treatment was performed for 70 s at 25 °C, and terminated by addition of 50 mM EDTA and heating for 10 min at 80 °C. DNA samples were analyzed with a 3730XL DNA Genetic Analyzer (Applied Biosystems) after purification, and data analyses were performed using GeneMarker software program v2.2.

### qRT-PCR

Total RNAs were isolated at various time points from mycelia of *S. avermitilis* grown in FM-I fermentation medium^59^ or on YMS solid medium. At each time point, mycelia were triturated in liquid nitrogen and suspended in 1 mL Trizol reagent (Tiangen) for RNA extraction. RNA samples were treated with DNase I (TaKaRa) to remove genomic DNA. 4 μg RNA was used to synthesize cDNA, and qRT-PCR analysis was performed to determine transcription levels of various genes, as described previously^58^. Transcription of housekeeping gene 16S *rRNA* was used as internal control. Each experiment was performed in triplicate.

### 5′-RACE

The transcriptional start site (TSS) of *sav_742* was identified using a 5′/3′ RACE kit (Roche, 2^nd^ Generation). 4 μg total RNA extracted from 48-h WT culture grown in FM-I was used for reverse transcription with 40 pmol gene-specific primer SD165A. After purification, the obtained cDNAs had oligo (dA) tails added to their 3′ ends by treatment with terminal deoxynucleotidyl transferase (TaKaRa) for 30 min at 37 °C. PCR was first performed using the tailed cDNA as template, and a second inner gene-specific primer SD165B and oligo (dT)-anchor primer. To obtain a single specific band, the first-round PCR product was diluted 100-fold, and used as template for second-round PCR with nested primer SD165D and an anchor primer. The final PCR product was purified and sent for sequencing. The TSS was found to be the first nucleotide adjacent to the oligo (dT) sequence.

### Production and analysis of avermectins

Fermentation of *S. avermitilis* ATCC31267 and its derivatives, isolation of avermectins, and HPLC analysis of avermectin yield were performed as described in our 2007 study^59^.

### SEM

Spores and mycelia of *S. avermitilis* strains grown on YMS agar for 2 or 6 days were observed by SEM. For specimen preparation, coverslips were embedded at an angle in agar inoculated with the strains, and gently lifted out after various periods of growth. Specimens were fixed with 2.5% glutaraldehyde and 1% osmium tetroxide for 2 h each, washed three times with phosphate buffer, dehydrated by ethanol concentration gradient (30%, 50%, 70%, 80%, 90%, 100%), dried in a LEICA critical point dryer (model EM CPD300), sputter-coated with a layer of gold, and examined by SEM (model S3400N, Hitachi).

## Additional Information

**How to cite this article**: Sun, D. *et al*. SAV742, a Novel AraC-Family Regulator from *Streptomyces avermitilis*, Controls Avermectin Biosynthesis, Cell Growth and Development. *Sci. Rep.* **6**, 36915; doi: 10.1038/srep36915 (2016).

**Publisher’s note**: Springer Nature remains neutral with regard to jurisdictional claims in published maps and institutional affiliations.

## Supplementary Material

Supplementary Information
