# Supplementary material for: SAV742, a Novel AraC-Family Regulator from Streptomyces avermitilis, Controls Avermectin Biosynthesis, Cell Growth and Development
Source: Sci Rep. 2016 Nov 14;6:36915. doi: 10.1038/srep36915 (PMC5107987; doi:10.1038/srep36915)
Supplement: Supplementary Information [file srep36915-s1.pdf]

Supplementary information

SAV742, a Novel AraC-Family Regulator from *Streptomyces avermitilis*, Controls Avermectin Biosynthesis, Cell Growth and Development

Di Sun, Jianya Zhu, Zhi Chen, Jilun Li, Ying Wen*

State Key Laboratory of Agrobiotechnology and MOA Key Laboratory of Soil Microbiology, College of Biological Sciences, China Agricultural University, Beijing 100193, China

* Author for correspondence. E-mail: wen@cau.edu.cn. Phone: +86-10-62732715.

Supplementary Figures:

Figure S1. Strategy for in-frame deletion of *sav_742* in *S. avermitilis*.

Figure S2. Determination of the *sav_742* TSS by 5'-RACE.

Figure S3. SAV742 directly activates *sig8* transcription.

Figure S4. Comparison of the predicted secondary structure of the DNA-binding domain of SAV742 (blue) with that of *S. griseus* AdpA (orange).

Supplementary Tables:

Table S1. Putative targets of SAV742.

Table S2. Strains and plasmids used in this study.

Table S3. Primers used in this study.

References

Figure S1

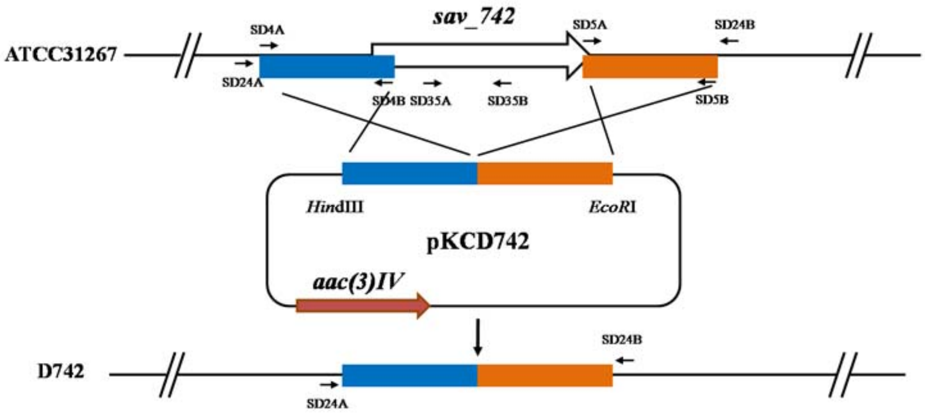

Figure S1. Strategy for in-frame deletion of *sav_742* in *S. avermitilis*. Thick arrow: gene and orientation of transcription. Short arrow: primer position. Block: homologous exchange fragment for *sav_742* deletion.

Figure S2

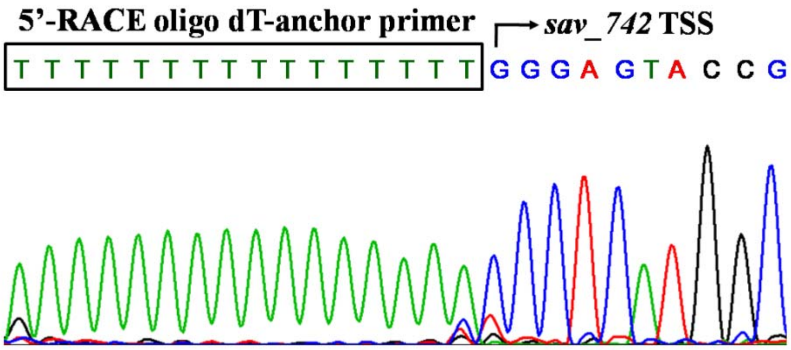

Figure S2. Determination of the *sav_742* TSS by 5'-RACE. Boxed area: sequence of oligo dT-anchor primer. Bent arrow: base of TSS.

Figure S3

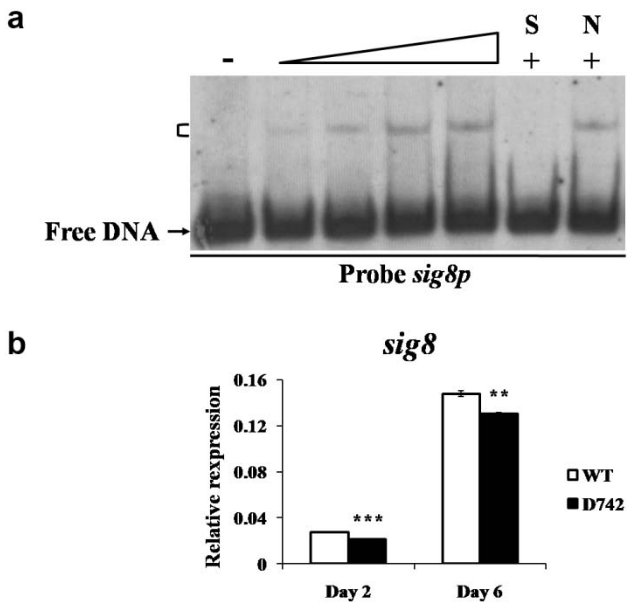

Figure S3. SAV742 directly activates *sig8* transcription. (**a**) EMSAs of His_6-SAV742 interaction with *sig8* promoter region. Procedures and notations same as for Fig. 2a. (**b**) qRT-PCR analysis of *sig8* transcription level in WT and D742 grown in FM-I for 2 or 6 days. Relative values were obtained using 16S *rRNA* as internal reference. **, P< 0.01; ***, P< 0.001 (Student's t-test).

Figure S4

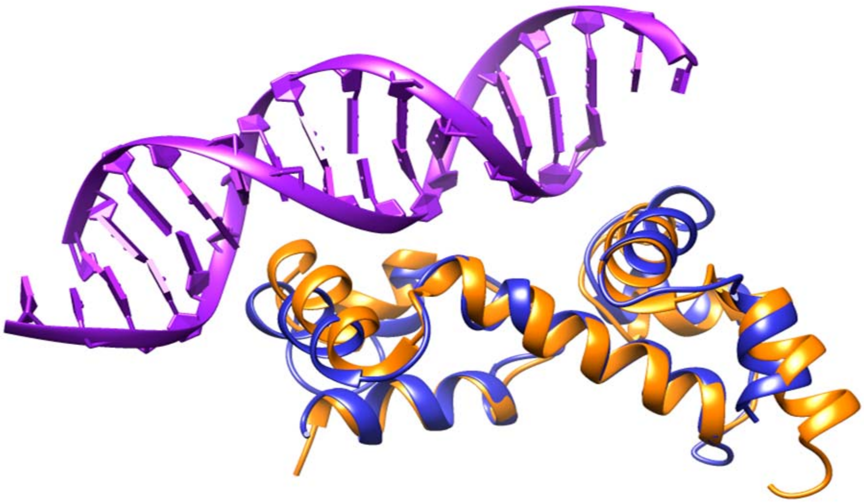

Figure S4. Comparison of the predicted secondary structure of the DNA-binding domain of SAV742 (blue) with that of *S. griseus* AdpA (orange). Purple color: DNA. The protein structure of the SAV742 DNA-binding domain was modeled using the SWISS-MODEL Workplace (https://swissmodel.expasy.org/). The Chimera program (v1.11rc) was used to align the SAV742 model with the structure of AdpA DNA-binding domain (Protein Data Bank ID: 3w6v).

Table S1. Putative targets of SAV742.

#	Accession number	Gene	Putative target sites	Function
**Regulatory function (350)**				
1	*sav_1934*	*alkA1*	GCCGCCACGCCGCC<br>GCCGCGGCCGCC<br>GCCGCCCTCGCC<br>GCCGCGGCCCGTGGAGGCCGGC<br>ACCGCCGGGGGCGGGGCTGGC	putative ADA-like regulatory protein
2	*sav_2085*	*alkA2*	GCCGCGGCGCGGGGCTGGC	putative ADA-like regulatory protein
3	*sav_7499*	*amfR*	GCCGCGGGAATCGGCACCGGC<br>ACCGAGAACCCGGC<br>GCCGCCCGCC	putative two-component system response regulator
4	*sav_3705*	*avaR1*	ACCGCCGAAGACAGGC	putative gamma-butyrolactone receptor protein
5	*sav_3702*	*avaR2*	GCCGCGCGGTACGGCTCGGC<br>ACCGCGTCGGC<br>GCCGCCCGCGCTGCCCGCTGCCGCC	putative TetR-family transcriptional regulator
6	*sav_1199*	*bdpC*	GCCGCCCGGGCCCTTCCCGGC	putative AraC-family transcriptional regulator
7	*sav_659*	*bdpD*	GCCGCCCTTCGTGACCGTTGCC<br>GCCGACCCGCC	putative AraC-family transcriptional regulator
8	*sav_1667*	*bdpL*	GCCGCGCGGCGCTGCC	putative AraC-family transcriptional regulator
9	*sav_1371*	*bdpM*	ACCGCGACTCCTGTGAGCTGCC	putative AraC-family transcriptional regulator
10	*sav_4130*	*bldC*	ACCGACTCGACTCGCGTCCGCC<br>ACCGATGGAATTGCC<br>GCCGCGTAGGC	putative MerR-family transcriptional regulator
11	*sav_4614*	*bldG*	ACCGCCCACAGCGCCGCCGCC	anti-sigma factor antagonist
12	*sav_5332*	*copZ*	ACCGCCACCGCC	putative copper chaperone
13	*sav_1252*	*cvnA1*	ACCGATCGCC<br>ACCGCCCCAGTGTTTCGCTGCC<br>ACCGCGGGTGTCCGGGTCGCC<br>GCCGATCTTGCCACCGGC<br>ACCGATGACGCGTCCGCC	putative sensor-like histidine kinase
14	*sav_1582*	*cvnA2*	GCCGCCCGGGTCCTCGCC	putative sensor-like histidine kinase
15	*sav_1633*	*cvnA3*	GCCGAGGAGCACAGTGCTCCTCGGC<br>ACCGCACATGATGCGTTGCC<br>GCCGAGGAGCACTGTGCTCCTCGGC<br>ACCGATAACCCCGCC	putative sensor-like histidine kinase

16	*sav_2179*	*cvnA4*	ACCGATTGGC<br>ACCGACCGAGAACCGGC	putative sensor-like histidine kinase
17	*sav_2188*	*cvnA5*	ACCGCTCATCGGC	putative sensor-like histidine kinase
18	*sav_2694*	*cvnA6*	ACCGCCGCGGGATCGAGATCCGGC<br>GCCGACACCGTCCCGCC<br>GCCGCCACTGGC	putative sensor-like histidine kinase
19	*sav_2962*	*cvnA8*	GCCGAACGGGAACGACCCCGCAGGC<br>ACCGCCGGTTTTGCC<br>GCCGCCGACGTCCAACCGGCCCGCC<br>GCCGCACGCGCCAGAGGAACTTGCC	putative sensor-like histidine kinase
20	*sav_3877*	*cvnA9*	GCCGCGCGGTCCGGCTCGCC<br>ACCGCCAACGCGGCGGACCGCTGCC<br>ACCGCGCGGCGCCGGC<br>GCCGCGTTGGC	putative sensor-like histidine kinase
21	*sav_6702*	*cvnA10*	GCCGAGCGTACTCCTGGC	putative sensor-like histidine kinase
22	*sav_6958*	*cvnA11*	GCCGCGCCCAGGC	putative sensor-like histidine kinase
23	*sav_3023*	*dasR*	GCCGATCGGACAGGTCGGC	putative GntR-family transcriptional regulator
24	*sav_2430*	*dcuS*	GCCGAAACCCCCGCATACCGCCGCC	putative two-component system sensor kinase
25	*sav_3690*	*fruR*	GCCGCGCTCAGGC	putative DeoR-family transcriptional regulator
26	*sav_6666*	*gylR*	GCCGAGCCATTGGC<br>ACCGCCCCGTTCGGC	putative IclR-family glycerol operon regulatory protein
27	*sav_5569*	*hrcA*	GCCGCCCCGCCCGGC<br>ACCGCCCTTGCC	putative HrcA-family heat-inducible transcriptional repressor protein
28	*sav_7365*	*hypX*	GCCGCCCACGCCGTCCTCGCCGGC<br>GCCGCGTCATGAACTCCTTGGC<br>GCCGATCCACCGTCCGGC<br>GCCGACTGCGATCGTCAGGC	putative [NiFe] hydrogenase
29	*sav_2396*	*kdpD*	ACCGCGCTCACCCTGCCTTTTGCC	putative two-component system sensor kinase for high-affinity potassium transport system
30	*sav_2463*	*lexA*	ACCGCTCCAGCAGGC<br>GCCGACCCGCGTCGGC<br>ACCGACCCCCTTTCCCCACACCGCC<br>GCCGACGCGGGTCGGC	putative SOS regulatory protein LexA
31	*sav_1214*	*litR*	GCCGCGAGAGCGGTCCGGCCGCC	putative MerR-family transcriptional regulator

32	*sav_7090*	*lpsR*	GCCGCATCGGTGAAATCGCC	putative *lpsA1* transcriptional activator
33	*sav_5976*	*malR1*	GCCGACAGTTACCCCGCC<br>GCCGCTCGAACGCTCGCGCTCGGC	putative repressor of *malE* transcription
34	*sav_4047*	*mprA*	GCCGCTCCCGGC<br>GCCGACCGCACGGCCGGCGCCGGC<br>GCCGACCCCACTGGC<br>ACCGATCCGGGGCCGACCCCCTGGC<br>ACCGACCTCCCGCTGATCCCCGGC<br>ACCGATGCTTTCCGCC<br>GCCGAGGAAGGCCCCGCC	putative two-component system response regulator
35	*sav_2462*	*nrdR*	GCCGACGCGGGTCGGC<br>ACCGACCCCCTTTCCCCACACCGCC<br>GCCGACCCGCGTCGGC<br>ACCGCTCCAGCAGGC	putative regulatory protein
36	*sav_2512*	*osaA*	ACCGCCCAGGCCGGACGGACCGGC<br>GCCGCGCGTCTGCC<br>GCCGAGTACTAGGACGCGGCAGGC<br>GCCGCGTCCTAGTACTCGGC	putative two-component system sensor kinase (response to osmoadaptation and osmotic stress)
37	*sav_2511*	*osaB*	GCCGCACCCCGCCTGTCGCC	putative two-component system response regulator (response to osmoadaptation and osmotic stress)
38	*sav_1148*	*popR*	GCCGAGGCGATGGGCCGGC<br>GCCGATCCCTTCCCCTTCGGC<br>GCCGAAGGGGAAGGGATCGGC	putative DNA-binding protein
39	*sav_5320*	*rbsR*	ACCGCCCCAGCC	putative LacI-family transcriptional regulator, ribose operon repressor
40	*sav_4738*	*rex*	ACCGAGTAGTCCAAACAGCC<br>ACCGCCTGCAGCC<br>ACCGCCCCGTCGAAAGCAGGC	putative redox-sensing transcriptional repressor
41	*sav_2444*	*rpoD*	ACCGCTGATTCGGC<br>GCCGCCGGGTCGCTCGCC<br>ACCGACGACCGCC	RNA polymerase major sigma factor, sigma-70 family (*hrdB*)
42	*sav_4734*	*rsbN*	GCCGCTCGGC<br>GCCGCGATCCGCC	putative anti-sigma factor
43	*sav_1094*	*rsbR*	ACCGCCTTTCCCCGGC	putative positive regulatory protein
44	*sav_1096*	*rsbT*	GCCGCGTCGCCGCCTGCCTGCC	putative anti-sigma factor
45	*sav_3012*	*rsbW*	GCCGAGGGTCGCC	putative anti-sigma factor
46	*sav_4262*	*rstP*	ACCGACTGGCTGATGACCGCC<br>GCCGAGCAGGC<br>ACCGCATGAGCCCTACTATCGGC	putative LacI-family transcriptional regulator

47	*sav_5141*	*sdrB*	GCCGCCGCGGAGCGCAGCC<br>GCCGCCAGCCATCGGC<br>GCCGCGAGGAGGCCTGCC<br>GCCGCCGATGGCTGGC	putative IclR-family transcriptional regulator
48	*sav_93*	*sig2*	GCCGCCCGGATCCCGGGCCGGC<br><br>GCCGCCGGCACACGTGGGTCCGCC	putative RNA polymerase ECF-subfamily sigma factor
49	*sav_527*	*sig4*	GCCGCTCAAGTCCGGC<br>ACCGACGCACCGCC<br>ACCGCACCCAGCC<br>GCCGAGCATCCGCGGCTTGCCGGC<br>GCCGCCCAAGCAGCC<br>GCCGCGGATGCTCGGC	putative RNA polymerase ECF-subfamily sigma factor
50	*sav_663*	*sig6*	GCCGCCCCCTCGCC	putative RNA polymerase ECF-subfamily sigma factor
51	*sav_741*	*sig8*	GCCGACCAAAGACACTTTGGTCGGC<br>GCCGCCGGCTGCTGGAGACCGGC	RNA polymerase alternative sigma factor, similar to *sco0600*, *sigB*
52	*sav_809*	*sig9*	ACCGCTCGGC	putative RNA polymerase ECF-subfamily sigma factor
53	*sav_1215*	*sig17*	GCCGCGAGAGCGGTCCGGCCGCC	putative RNA polymerase ECF-subfamily sigma factor
54	*sav_1808*	*sig18*	GCCGCGCACCGGCAGGC	putative RNA polymerase ECF-subfamily sigma factor
55	*sav_1873*	*sig19*	GCCGCGTGGCCCTGCC<br>ACCGCCTGCC	RNA polymerase alternative sigma factor, similar to *sco6520*, *sigK*
56	*sav_2597*	*sig20*	GCCGCCGGCCGCCTCGCC	putative RNA polymerase ECF-subfamily sigma factor
57	*sav_3038*	*sig22*	GCCGCATGGGTTCGACCTTCTGCC	putative RNA polymerase ECF-subfamily sigma factor
58	*sav_3117*	*sig23*	GCCGACGCCTTTGTCGCC<br>GCCGCACCTCCCCTATCGGC	putative RNA polymerase ECF-subfamily sigma factor
59	*sav_3318*	*sig24*	GCCGAGGCACCGGC	putative RNA polymerase ECF-subfamily sigma factor
60	*sav_3351*	*sig25*	GCCGCGAGCGCACCCCCGCC	putative RNA polymerase ECF-subfamily sigma factor
61	*sav_3385*	*sig27*	GCCGATGGCAACGGCCGGGGCCGCC	putative RNA polymerase ECF-subfamily sigma factor
62	*sav_3490*	*sig29*	ACCGCAGTGCACGTATTGGC	RNA polymerase alternative sigma factor, similar to *sco3068*, *sigI*
63	*sav_3844*	*sig31*	GCCGCGGATGGTAGGGGCTCGGC	putative RNA polymerase ECF-subfamily sigma factor

64	*sav_3888*	*sig32*	GCCGACACGGCACAACCCGGC	putative RNA polymerase ECF-subfamily sigma factor
65	*sav_4185*	*sig34*	GCCGAACCGGC	RNA polymerase alternative sigma factor, similar to *sco4035*, *sigF*
66	*sav_4186*	*sig35*	GCCGACATGTGACATTCTGCC<br>GCCGCGGCCTCGCC<br>GCCGCGGCAAACGCGCTTCCGGC	RNA polymerase alternative sigma factor, similar to *sco4034*, *sigN*
67	*sav_4207*	*sig36*	GCCGATCCGCC	putative RNA polymerase ECF-subfamily sigma factor
68	*sav_4427*	*sig39*	GCCGACAGAAGCAGGACCGGC<br>GCCGACTTCGCC	putative RNA polymerase ECF-subfamily sigma factor
69	*sav_4561*	*sig40*	ACCGCGGTACAGGC<br>GCCGACCCCGCATGCCCGGC<br>ACCGCAAAGACGCTCCCCGCC	putative RNA polymerase ECF-subfamily sigma factor
70	*sav_4706*	*sig41*	ACCGACCGCCGGC<br>GCCGATACACAGCCCCCGGC<br>ACCGACGGACAGCCGAGCGTTCGCC<br>GCCGCGCTTCTGGC	putative RNA polymerase ECF-subfamily sigma factor
71	*sav_4735*	*sig42*	ACCGCTCCCGCC	putative RNA polymerase ECF-subfamily sigma factor, *bldN*
72	*sav_4987*	*sig43*	GCCGCGGCTCGAGTCGTGGCTTGGC<br>GCCGCGGCTCGGCGCCTGGCCCGGC	putative RNA polymerase ECF-subfamily sigma factor
73	*sav_5123*	*sig45*	ACCGATGCGCACACTGCC<br>GCCGACGGAGATTTCACTCTCAGGC	putative RNA polymerase ECF-subfamily sigma factor
74	*sav_5398*	*sig46*	GCCGAACGTTTCCGGC<br>GCCGCAGGATCGGGGCCCGGCCGCC<br>GCCGCCCGGC<br>GCCGCAGGCGCCGGAAACGTTCGGC	putative RNA polymerase ECF-subfamily sigma factor
75	*sav_5679*	*sig47*	GCCGCGCTCCGAGGACTCAGGC	putative RNA polymerase sigma factor, *hrdA*
76	*sav_5903*	*sig48*	GCCGCCGGTCCTGTGCCGTCCGGC	putative RNA polymerase ECF-subfamily sigma factor
77	*sav_6077*	*sig49*	GCCGCGGGCATGCGAAAGGCCCGCC	putative RNA polymerase ECF-subfamily sigma factor
78	*sav_6337*	*sig50*	GCCGCTCCGGACCCGGACATCCGGC	putative RNA polymerase ECF-subfamily sigma factor
79	*sav_6385*	*sig52*	ACCGCCGGGTACGAGGAGTCTCGCC	putative RNA polymerase ECF-subfamily sigma factor
80	*sav_6582*	*sig53*	ACCGCATTCGGAGCGGTCGCC<br>GCCGATGAAACAACTGGC	putative RNA polymerase ECF-subfamily sigma factor
81	*sav_6785*	*sig54*	GCCGCCCCGGCAACCTCCCGCC<br>ACCGCGGGACCTCAGCC	putative RNA polymerase ECF-subfamily sigma factor

			GCCGCCGCCCCGGCGGGAGGTTGCC	
82	*sav_7068*	*sig55*	GCCGACACCGGTTCCGCC	putative RNA polymerase ECF-subfamily sigma factor
83	*sav_7292*	*sig56*	GCCGCACCGCCCGAGTCGCC<br>ACCGCAACGGCGTCACGTCCGGC<br>ACCGATCGGGACTGCC<br>ACCGCGCTCCGCC	putative RNA polymerase ECF-subfamily sigma factor
84	*sav_7400*	*sig57*	GCCGCCGTCGCC<br>ACCGCGCATTCGGC	RNA polymerase alternative sigma factor, similar to *sco7341, sigG*
85	*sav_7424*	*sig58*	GCCGCGACCGCACCGGC	putative RNA polymerase ECF-subfamily sigma factor
86	*sav_3304*	*sig59*	ACCGCAGGGCCGGGCCTGGC<br>GCCGCCCCTGGC	putative RNA polymerase ECF-subfamily sigma factor
87	*sav_3352*	*smrA*	GCCGCGAGCGCACCCCCGCC	putative two-component system response regulator
88	*sav_4268*	*ssfR*	ACCGCACCCACCGGATCAATCGGC<br>GCCGCTCTGCC	putative IclR-family transcriptional regulator
89	*sav_4997*	*wblB*	GCCGACGGAAACTGCCGCCTGCC<br>GCCGAAGATGGCTCACCGCC	putative WhiB-family transcriptional regulator; putative role in cell cycle control
90	*sav_3216*	*wblI*	ACCGCACCCTCCAGGCCCGCC<br>ACCGCTGGCCTTCGGGCGGCCCGCC	putative WhiB-family transcriptional regulator; putative role in cell cycle control (*whiD*)
91	*sav_5042*	*whiB*	ACCGATGGCTGTAGGC<br>ACCGATTTCGGC	putative WhiB-family transcriptional regulator, WhiB protein
92	*sav_2630*	*whiG*	ACCGACAGGTCACCCAGGC<br>GCCGCCCGGGGTCCCGCCCATTGCC	RNA polymerase alternative sigma factor
93	*sav_4998*	*whiK*	GCCGAAGATGGCTCACCGCC<br>GCCGACGGAAACTGCCGCCTGCC	putative two-component system response regulator, *bldM*
94	*sav_1693*	*whmD*	GCCGAGTCTTGCACCGCC<br>GCCGACTCCGCGCGCCGCC<br>GCCGCCCGGCACGATCTCGCC	putative WhiB-family transcriptional regulator, *wblH*
95	*sav_29*		ACCGCCTTCGCGCCCGCCAGCC<br>ACCGCGGGCGTGGAGGCGCTGCC<br>GCCGCCTGCGGCCGTCTGCC	putative PadR-like family transcriptional regulator
96	*sav_53*		ACCGCACGGTCCGCC	putative transcriptional regulatory protein
97	*sav_67*		GCCGAGTCCGGTGACGGACAGCC<br>GCCGCTGCGTCCGACGCCTCCGCC	putative regulatory protein

98	*sav_73*	GCCGCCGGAAGCCTGTGCCAGGC	putative two-component system response regulator
99	*sav_82*	ACCGCCGAGGCACTGCCCGGC	putative TetR-family transcriptional regulator
100	*sav_111*	GCCGCAGTCACCGCCCGCC<br>GCCGCTCCGCC<br>GCCGCCTCGTGCCGCGCCGCC<br>GCCGCTCCGCC<br>GCCGAGGCTCTGCGTGCCCCGGC<br>GCCGACTGTCACAGGC	putative LuxR-family transcriptional regulator
101	*sav_134*	GCCGCACGCTTTTGCC<br>GCCGCCGCCTGCC	putative two-component system sensor kinase
102	*sav_143*	GCCGCTCAGAGGCTCGGC	putative TetR-family transcriptional regulator
103	*sav_146*	GCCGACTTTCGGACGGTCCTGCC	putative TetR-family transcriptional regulator
104	*sav_151*	GCCGAGCGGCCGGC<br>GCCGCTCGGC	putative TetR-family transcriptional regulator
105	*sav_231*	GCCGAGAGGTCGCCGGC	putative MerR-family transcriptional regulator
106	*sav_234*	GCCGCCTGCC	putative regulatory protein
107	*sav_235*	GCCGCCTGCC	putative anti-sigma factor antagonist
108	*sav_292*	GCCGCCACTCCATGCCCAACCGGC	putative TetR-family transcriptional regulator
109	*sav_306*	GCCGCCGGGTGGATCCAGCC<br>GCCGACCGGC<br>ACCGAAGCCGGC<br>ACCGACAGACTGCC	putative transcriptional regulatory protein
110	*sav_323*	GCCGAAGAGCGGCGCTGTAACAGGC<br>GCCGCTCTTCGGCAGCCAGCC	putative MerR-family transcriptional regulator
111	*sav_373*	ACCGCACAGTCCGGC	putative LysR-family transcriptional regulator
112	*sav_431*	GCCGAGTCCCGGC<br>GCCGCGCCACCTCCATTCGGC<br>ACCGCCTCGGC<br>ACCGCGCCAGTGGTCCTGCC	putative TetR-family transcriptional regulator
113	*sav_436*	GCCGATGGCAACGTCGGC<br>ACCGCGGACCGGC<br>GCCGACGTTGCCATCGGC<br>GCCGCGCTGATCGGC	putative TetR-family transcriptional regulator
114	*sav_448*	ACCGCCTTGACCGCGTCGGC	putative TetR-family transcriptional regulator
115	*sav_472*	ACCGCAGAGATCGGC	putative regulatory protein

116	*sav_508*	GCCGACCCCCGGCCGTTCGCC	putative TetR-family transcriptional regulator
117	*sav_520*	GCCGCCGCCCTTGCC	putative transcriptional regulator
118	*sav_537*	GCCGCCTGCACCGCC<br>GCCGAGTGGTGGCAGCC	putative Sir2-family regulator protein
119	*sav_554*	GCCGCGACTCTGGC<br>GCCGCACAGCC<br>ACCGAAGTGGGATTCGGC<br>GCCGCCTGTCCAGCC<br>GCCGCACGGCTGAAGGGCCTGGC	putative LacI-family transcriptional regulator
120	*sav_576*	GCCGCCCACCAGCACACAGCC<br>GCCGAGGAGACCCTGGC	putative TetR-family transcriptional regulator
121	*sav_577*	ACCGCGTCCGGC	putative TetR-family transcriptional regulator
122	*sav_588*	ACCGAATCTTCCCGGC	putative AsnC-family transcriptional regulator
123	*sav_621*	GCCGCCCTTCGGC<br>ACCGCCGCCCACAAGGGAGACCGCC	putative MarR-family transcriptional regulator
124	*sav_636*	ACCGAACCGGC<br>ACCGACTGACAATTGGC<br>ACCGCGCTGCC<br>GCCGCTGGCGCCCCTTTGACCAGGC<br>GCCGCAGTCGCCGAGTCGCC	putative AraC-family transcriptional regulator
125	*sav_648*	GCCGACGTGTCAGGGCCGGC<br>ACCGCGTCGGCACCGGGGACCCGCC<br>ACCGCTGTGTTCCCGCC<br>GCCGACCAGCC<br>GCCGCCGTTGAGACGCCTGGC<br>GCCGACGTGTTCGCCGTCGCC<br>GCCGCGCGGCCGGC<br>GCCGCCAGCC	putative two-component system response regulator
126	*sav_669*	GCCGCCTCCCGGC<br>ACCGCCGTCCGCCGCAACAGCC<br>GCCGCCGACGAGCCACCCCTCCGGC	putative TetR-family transcriptional regulator
127	*sav_705*	GCCGCCGACGGCGATCCCGGC<br>ACCGCCATCTGCGCTGGC<br>GCCGCGGGCCGGC<br>GCCGCGTACAACAGCCGGGATCGCC	putative TetR-family transcriptional regulator
128	*sav_722*	GCCGACGCCAACGACATCGCC<br>GCCGCACCGCGACGGCGGCCAGGC<br>ACCGCTCACACCCTCGCC	putative AraC-family transcriptional regulator
129	*sav_729*	ACCGATCCGGC	putative LysR-family transcriptional regulator

130	*sav_742*	GCCGCCCGTTCATTCCAGCAGCC<br>GCCGAACGCAAAATCGCTCGGC<br>GCCGAGCGGACGCACTCGGC<br>GCCGCCTCGTACCCGGC<br>GCCGAGTGCGTCCGCTCGGCCGGC<br>GCCGACATCCGGC	putative AraC-family transcriptional regulator
131	*sav_771*	GCCGCCCTGGTGCCTGCC	putative LysR-family transcriptional regulator
132	*sav_775*	GCCGAACCCCGAGGCCGAAGCAGCC<br>GCCGCGGCCTGGGGCTGCTTCGGC	putative TetR-family transcriptional regulator
133	*sav_901*	ACCGAACCACCCCAGGC	putative transcriptional regulator
134	*sav_913*	ACCGCTGTTGGC	putative TetR-family transcriptional regulator
135	*sav_932*	GCCGCTCCTCCTGCAGCC<br>GCCGCTCCGCC<br>ACCGCTCGACCATATCCAGGC	putative two-component system response regulator
136	*sav_965*	GCCGCAGCCGGC<br>GCCGCCCGGTACGCCGGC	putative transcriptional regulator
137	*sav_972*	ACCGACTGCGACGAAGAACCGCC	putative LacI-family transcriptional regulator
138	*sav_1047*	GCCGCACTCCGCC<br>GCCGCCCGACGGGGCAGGC<br>GCCGCCAGCGCAACGCCAGGC	putative regulatory protein
139	*sav_1056*	GCCGCTCGGC	putative MarR-family transcriptional regulator
140	*sav_1085*	ACCGATCGGCATCGAACCGCC<br>ACCGACACGGAGATTGCC	putative two-component system sensor kinase
141	*sav_1091*	ACCGCGAAGCAGCC<br>GCCGATGCCCGGCGGGACGCCCGGC	putative anti-sigma factor antagonist
142	*sav_1099*	ACCGCTGTGGCCTGTGGCTCGCC<br>GCCGCCTCGACCAGGC	putative MarR-family transcriptional regulator
143	*sav_1100*	GCCGCCTCGACCAGGC<br>ACCGCTGTGGCCTGTGGCTCGCC	putative regulatory protein
144	*sav_1102*	ACCGCGACCAGTTGTCGTCGGC<br>GCCGCAGTCCGCCTCACCGTCGCC<br>ACCGCCCGGGGTGTCCCCGCCGGC<br>ACCGCCGCGCAGGC<br>GCCGAGCGCCCGGC	putative anti-sigma factor antagonist
145	*sav_1103*	GCCGAGGGTAGGC<br>GCCGCGTAGCCGGACAGCC	putative regulatory protein
146	*sav_1153*	ACCGATCCGGC<br>GCCGCGTCCCCGCC	putative regulatory protein
147	*sav_1163*	ACCGCAGATTCCGCC	putative LysR-family

			transcriptional regulator
148	*sav_1209*	GCCGCATGTCAGCCGTAGGC<br>ACCGCGGCTCTTTTTCCGGC<br>ACCGCCCGTCCGCC	putative LysR-family transcriptional regulator
149	*sav_1222*	GCCGACGTCGATCGGCGGCCGGC<br>GCCGCCGATCGACGTCGGC<br>GCCGCCCTTGATCCGCC<br>GCCGAGGGGAGCCCGGCGCCGGC	putative ROK-family transcriptional regulator
150	*sav_1236*	GCCGCGCTGCCCGCCCCGGTTGCC	putative regulatory protein
151	*sav_1271*	ACCGAACACGTCGTCCGCGTCGGC<br>ACCGCTCACTTCCTTGGC	putative DNA-binding protein
152	*sav_1278*	ACCGCCTCGGC<br>GCCGCCCGGC	putative CRP-like regulatory protein
153	*sav_1282*	GCCGCCGGTCAGGACCATCCGGC<br>ACCGCCCCGCCGACTGGCTGGC<br>ACCGAATCCGGTCAGGC	putative two-component system response regulator
154	*sav_1305*	GCCGAACTGCCCGGC<br>GCCGAATCCGACCCGCGTCGCC	putative GntR-family transcriptional regulator
155	*sav_1352*	ACCGCGTCTCGTCCCAGGC<br>GCCGCCGGGGGATCCAGCC<br>ACCGCGGTCGGGAGCGGTCGGC	putative LysR-family transcriptional regulator
156	*sav_1359*	GCCGCCTGCC	putative TetR-family transcriptional regulator
157	*sav_1392*	GCCGATTACACAGGCCGCC	putative transcriptional regulator
158	*sav_1406*	GCCGCCGCGTGCAGGGGCCGCC<br>GCCGACGGCACGAGCCTCGCC	putative GntR-family transcriptional regulator
159	*sav_1433*	ACCGCGGGCCGCGCCATCGCC	putative DeoR-family transcriptional regulator
160	*sav_1437*	GCCGACGTACGCAGCC<br>GCCGCCTGCTCAGCAGGC<br>GCCGCCCGCTGTTGATGACCCGGC<br>GCCGCCTGCTGAGCAGGCGGCCGGC	putative regulatory protein
161	*sav_1439*	ACCGCCGTCGGC	putative TetR-family transcriptional regulator
162	*sav_1442*	ACCGCGGGGCCCCCGGC	putative ROK-family transcriptional regulator
163	*sav_1465*	GCCGACGCCGCC	putative two-component system sensor kinase
164	*sav_1466*	ACCGCTTCGTCTCACTCTTCGCC	putative TetR-family transcriptional regulator
165	*sav_1517*	GCCGAAGGCGGAACCCGGCCCTGCC<br>GCCGCTGCTGCC	putative LysR-family transcriptional regulator
166	*sav_1524*	GCCGCGGCGGTGAGCCATGCCCGGC	putative TetR-family

		GCCGACCGTCAGGGCCGCC<br>ACCGCCTCTCCCCCGCC	transcriptional regulator
167	*sav_1541*	ACCGCCCCTTCGGC<br>ACCGATCGCCCACCGGC	putative TetR-family transcriptional regulator
168	*sav_1612*	GCCGCCGCCGTTCAGGC<br>GCCGCGGGATGTCCCGGC	putative two-component system response regulator
169	*sav_1619*	ACCGCGGCAGGAACCCCCGGC<br>ACCGCGCCGCC	putative GntR-family transcriptional regulator
170	*sav_1637*	GCCGCTGTGTAGGGTTGCC<br>GCCGCCCACCGTGACAGGC	putative TetR-family transcriptional regulator
171	*sav_1644*	GCCGACGTGAAGCGGTAGCC<br>ACCGCTTCACGTCGGC	putative regulatory protein
172	*sav_1669*	ACCGCGACCCGCC<br>GCCGCGCCCGGCCCACCGCGCCGCC<br>GCCGCCGATCTGCC<br>GCCGCCGAACAGCC	putative transcription accessory protein
173	*sav_1678*	ACCGCGTCGCCGCC	putative MerR-family transcriptional regulator
174	*sav_1707*	GCCGCGTCGCC	putative regulatory protein
175	*sav_1765*	ACCGACAGCAGGGCCGAGCCGCC	putative AraC-family transcriptional regulator
176	*sav_1778*	GCCGCCGGACGGGTCGCC	putative TetR-family transcriptional regulator
177	*sav_1786*	ACCGCGCGGCGCCGTCGCC<br>GCCGCCCCCACCGCC<br>GCCGCGGCGCCGGCTCGGC	putative MerR-family transcriptional regulator
178	*sav_1811*	GCCGCCCGTGTCGCCGAAATCAGCC<br>GCCGCTCATGGGGAAGACGACAGGC<br>ACCGCACTCCTTCTCCGGC<br>GCCGACCCCTGCTCAAAGCCTGGC<br>GCCGAACTCCGGACCGCC	putative regulatory protein
179	*sav_1829*	ACCGAGGGGCGCCAGGC<br>GCCGCGGAGTCGTCAGGCGGTCGCC<br>GCCGCCGGCTCGTACGCCGCC<br>GCCGCCGCCTGGC<br>GCCGAGGCTGCCCGCC<br>ACCGACGACTTTCGCC	putative ROK-family transcriptional regulator
180	*sav_1830*	GCCGCCACTGCC	putative GntR-family transcriptional regulator
181	*sav_1839*	GCCGCCCAGCGGGATCGGCTCGGC<br>ACCGAGGCCGCC	putative MarR-family transcriptional regulator
182	*sav_1990*	GCCGACGTCCGGC<br>GCCGCGGCCTCCCCGCAGGCAGCC<br>GCCGCGGCTTCGGACGGACAGGC	putative two-component system sensor kinase

183	*sav_1997*	ACCGACCGACAGGTAGGC	putative IclR-family transcriptional regulator
184	*sav_2033*	GCCGCCGGGCAGTCGCC	putative LuxR-family transcriptional regulator
185	*sav_2056*	GCCGATGCGGCGGACATCCGCC<br>GCCGCCATGCCTTCGGCGTCAGGC<br>GCCGCATCGGC<br>GCCGCGTCGGC<br>GCCGCTCTTGAGGCCCGCGGCAGGC<br>GCCGAAGGCATGGCGGCTGGC	putative TetR-family transcriptional regulator
186	*sav_2070*	ACCGAAACCATGAAGGAGTTGGC<br>GCCGCCGCACACGATCTGGC	putative regulatory protein
187	*sav_2076*	GCCGAACCGTGCTGCC<br>GCCGAGGGGCTCCGCC<br>ACCGCCCCCTCGCGCCTGCC<br>GCCGAGGAGGCCCCGATTGCC	putative regulatory protein
188	*sav_2081*	GCCGACACCGGCGTCCACACCGGC<br>GCCGACGTCGGCCTGGC<br>GCCGACGTCGGC	putative LuxR-family transcriptional regulator
189	*sav_2098*	GCCGCCTCGCTCGATCCGACCGGC<br>GCCGCGCGTCTGGGTATATTAGGC<br>ACCGCGAAGGCGGGCCCGCC	putative TetR-family transcriptional regulator
190	*sav_2114*	GCCGCCCGAACGGATATGTGTCGGC<br>GCCGCCGACCCGACCGTCGCC	putative LacI-family transcriptional regulator
191	*sav_2124*	ACCGCGGACGCCGCC<br>ACCGCAGGGCCGCTCGCC	putative regulatory protein
192	*sav_2248*	GCCGCGCCCCGATGGAAAACCGGC<br>GCCGCCACGATGAGGCCCCGCC	putative ROK-family transcriptional regulator
193	*sav_2257*	GCCGCATCCCCGTCGCC<br>GCCGCATCCCCGGC	putative two-component system response regulator
194	*sav_2301*	GCCGCCACCGAGCGCGGCCCGCC<br>GCCGCGCTCGGTGGCGGCCCGCC<br>GCCGACACGCCCACACCCCGGC<br>ACCGCGCGGAGGCCGGC	putative regulatory protein
195	*sav_2332*	GCCGCTCCGGC	putative ArsR-family transcriptional regulator
196	*sav_2371*	GCCGCTTGGCGGGAATATTGGC<br>GCCGCAACAGCTGCC<br>GCCGATAGGCTCTGCC	putative two-component system response regulator
197	*sav_2419*	GCCGATTGACGTGTGTTCAGGC	putative GntR-family transcriptional regulator
198	*sav_2454*	GCCGACCGCC	putative TetR-family transcriptional regulator
199	*sav_2499*	GCCGAGGCGGACCACCCGCCGCC	putative AraC-family

			transcriptional regulator
200	*sav_2569*	GCCGCCGGATCTTCCGGAGGTTGCC	putative LacI-family transcriptional regulator
201	*sav_2601*	GCCGCGCCGCC	putative PadR-family transcriptional regulator
202	*sav_2629*	GCCGCGGAGGGGAGCCACTCCGGC<br>GCCGCTCGAATCCTTGGC	putative TetR-family transcriptional regulator
203	*sav_2652*	ACCGACCTCCTCGGTCGCC<br>ACCGCTTCACCCACTTCCGGC<br>GCCGCGTTTACCGGC<br>GCCGACTTGCCTCCGGC	putative regulatory protein
204	*sav_2687*	ACCGCGCGTCCGCCGCC<br>GCCGACACAGGCCGGC	putative IclR-family transcriptional regulator
205	*sav_2727*	GCCGCCGTGAATGGACCGCC	putative TetR-family transcriptional regulator
206	*sav_2729*	ACCGACGTACTCGGGCCCCCCGGC<br>ACCGCCGCCCGTCCGCC<br>GCCGCTCCTCCGGAACCGGC	putative LuxR-family transcriptional regulator
207	*sav_2810*	GCCGACGAAACGAGCCGGC<br>GCCGCCGAATCAGGC	putative AsnC-family transcriptional regulator
208	*sav_2814*	GCCGCGAGGCAGGC	putative TetR-family transcriptional regulator
209	*sav_2834*	GCCGCCGGCCGCGGGCGCTCGGC<br>GCCGCGCAACTGCCGGC<br>ACCGCGATATGACCGGC<br>GCCGAAGCCCCTCAGTCTCGCC<br>GCCGAGCGCCCGCGGCCGGC<br>ACCGCGCTCGACGAGCCCGCC<br>GCCGCCCCGGCCGGC	putative MarR-family transcriptional regulator
210	*sav_2914*	ACCGCAGGTGAGCCCCGTATCGCC<br>GCCGCCCCCGGC	putative transcriptional regulator
211	*sav_2921*	GCCGCTCGGCTGACTTCCCAGCC<br>GCCGCCCGCC	putative two-component system response regulator
212	*sav_2955*	ACCGCCAGGCAGGC<br>GCCGCCGCGCCGCCAGGC<br>GCCGCGCCGCCAGGC<br>GCCGCCGGGCAGGC<br>ACCGCACCCCGAGGCGGGCCCCGCC	putative TetR-family transcriptional regulator
213	*sav_3017*	GCCGATGTGGGACAGGC<br>GCCGCCCGCC	putative two-component system sensor kinase
214	*sav_3049*	GCCGATCTTCGCCGGC<br>GCCGCTCGCC<br>GCCGAAGGGTGCGCCGGC<br>ACCGAAGGGCGTGCGCCGGC	putative TetR-family transcriptional regulator

215	*sav_3070*	GCCGCCACCCGGC	putative WhiB-family transcriptional regulator
216	*sav_3094*	GCCGCAAGGCACTCATCGGC<br>GCCGCGCCGCC	putative TetR-family transcriptional regulator
217	*sav_3140*	ACCGACAGCC	putative regulatory protein
218	*sav_3297*	ACCGCCACCTTCGTGAAGCCGCC	putative regulatory protein
219	*sav_3338*	ACCGCTCGCCGCGCCCGGC<br>GCCGCCGGGCCGCCGCTCCCAGGC<br>GCCGCGCGCCGCGCGTCGGC<br>GCCGCAGGGCAGCC	putative LuxR-family transcriptional regulator
220	*sav_3339*	ACCGAGCCGCC	putative DeoR-family transcriptional regulator
221	*sav_3424*	ACCGCTCCGTCCTGCC	putative regulatory protein
222	*sav_3471*	GCCGCCGCGGCCGCCCGGC<br>ACCGCTGATCGAACACTTCCCGCC	putative two-component system sensor kinase
223	*sav_3481*	GCCGCAGGCCGGACCATCCACAGCC<br>GCCGCGGCTCTTGTCATCGGC<br>GCCGCCGGCTGTGGATGGTCCGGC<br>GCCGCGGCGTGGTCGGC	putative two-component system response regulator
224	*sav_3489*	ACCGCAGTGCACGTATTGGC	putative anti-sigma factor antagonist
225	*sav_3537*	ACCGCTCACCGCCCGGC<br>GCCGAACCGTTTATCCACAGGC<br>GCCGACAGCTGTTCCTTTGCCGGC<br>GCCGCTTCACTGCC	putative MazG-family transcriptional regulator
226	*sav_3560*	GCCGCCCGTAGGC	putative two-component system sensor kinase
227	*sav_3572*	GCCGCAGCCGCACGGCCGCCGCC	putative two-component system response regulator
228	*sav_3631*	GCCGACCCCGCC<br>ACCGATGGCACCGGC<br>GCCGAAAGGTCCCTGGC<br>GCCGCTCCCGCC	putative two-component system sensor kinase
229	*sav_3632*	GCCGCTCCCGCC<br>GCCGAAAGGTCCCTGGC<br>ACCGATGGCACCGGC<br>GCCGACCCCGCC	putative regulatory protein
230	*sav_3667*	GCCGCCACCGCCGCC<br>GCCGACGCACTCGCC	putative LuxR-family transcriptional regulator
231	*sav_3693*	GCCGCGAGCCCGCAGCC<br>GCCGAGCTTCGGC<br>GCCGACGGGTCGCC<br>GCCGAAGCTCGGC<br>ACCGAAGCTCGGC	putative TetR-family transcriptional regulator

232	*sav_3697*	GCCGCGTGCAGCC	putative MarR-family transcriptional regulator
		GCCGCAGTCATGTCCGCCGCCGCC	
233	*sav_3701*	GCCGAGCCCCCCGTCCTCGCC<br>GCCGAGCCCTCCGCC<br>GCCGAGAGACGTACGCCGCC<br>GCCGCGCCCGCC	putative IclR-family transcriptional regulator
234	*sav_3721*	GCCGCTCGCC<br>GCCGAGCAGCC<br>ACCGCCCGTGAACAGGC	putative GntR-family transcriptional regulator
235	*sav_3837*	GCCGACTCCAGGC<br>ACCGCCCCGCC	putative regulatory protein
236	*sav_3843*	GCCGCGGATGGTAGGGGCTCGGC	putative anti-sigma factor antagonist
237	*sav_3850*	GCCGACTTCTTTTGTTCGTTCCGGC<br>GCCGCTGTCGTTGCC	putative transcriptional regulator
238	*sav_3977*	ACCGCATAGGATTGCC	putative AraC-family transcriptional regulator
239	*sav_4003*	GCCGCGCTTCGACACGAAGATCGCC	putative AraC-family transcriptional regulator
240	*sav_4015*	ACCGCCTCCGCCTCCGCC	putative MerR-family transcriptional regulator
241	*sav_4017*	ACCGCGCGGGCCGCC	putative TetR-family transcriptional regulator
242	*sav_4023*	GCCGACCGAGCTCCCGGC<br>ACCGCACAGCC	putative GntR-family transcriptional regulator
243	*sav_4042*	ACCGCAAGACGACCCTGGC	putative two-component system response regulator
244	*sav_4110*	GCCGCGCAGGGTTGGCCGAATTGCC	putative TetR-family transcriptional regulator
245	*sav_4111*	GCCGAGACGGCCCGCAGCC	putative transcriptional regulator
246	*sav_4158*	ACCGCGCCTCACAGCCACCGCC<br>ACCGCCCCACAACCGCGCCGCC<br>ACCGCGCCTCACAGCC<br>GCCGCGCCGCC	putative two-component system sensor kinase
247	*sav_4193*	ACCGCGCCCGCC	putative TetR-family transcriptional regulator
248	*sav_4198*	GCCGCACACGCCTTCCCAGCC	putative two-component system response regulator
249	*sav_4205*	GCCGCGAGCGCAACCACTGCC<br>GCCGATTGGC<br>ACCGCACCGTGCCCCGCC<br>ACCGCAGAGGCCATCTTGCC	putative two-component system sensor kinase/response regulator, bifunctional protein

250	*sav_4254*	GCCGCCGTCCGGC	putative MerR-family transcriptional regulator
251	*sav_4281*	ACCGCGTTTGCATATAGCC	putative MarR-family transcriptional regulator
252	*sav_4295*	ACCGAAGTTCACCCCAAATCAGGC<br>ACCGATTCGTAGGAAGCAAGTTGCC	putative PadR-family transcriptional regulator
253	*sav_4361*	ACCGATTGTTCGGTCGTCGGC<br>GCCGCCCGCCCGCC	putative AsnC-family transcriptional regulator
254	*sav_4380*	GCCGCCGCAGTGATCACTGCCGGC	putative GntR-family transcriptional regulator
255	*sav_4398*	ACCGCCCGCCAACCGCC	putative regulatory protein
256	*sav_4406*	GCCGACTCGCC<br>GCCGCCGCACAGGC	putative LysR-family transcriptional regulator
257	*sav_4412*	GCCGAGCCGGC<br>GCCGCGGGCACCCGGGCCGCC<br>GCCGCGGCCGGACTCCTCGCC<br>GCCGACTCGGC<br>GCCGAGTCGGCCGCCCGGC	putative transcriptional regulator
258	*sav_4416*	GCCGCGCGGACCGCACCGCC	putative two-component system response regulator
259	*sav_4448*	GCCGCGCACAGAACGCCAGATTGCC	putative MarR-family transcriptional regulator
260	*sav_4510*	GCCGACAGGC<br>GCCGAAATCGCCGGTTGACCGGC<br>ACCGCGCCCCGGC<br>ACCGCAAGGTCCCCGGAGCAGCC	putative LuxR-famiry transcriptional regulator
261	*sav_4511*	GCCGCTGCTCTTTCCGCC	putative LuxR-family transcriptional regulator
262	*sav_4536*	GCCGCCGGGCCGGTTGCC<br>GCCGCCTTCCGGAAGCTGCC<br>GCCGCTCCGGC	putative two-component system sensor kinase
263	*sav_4542*	GCCGCGCAGCTCGTCGAGGCAGGC<br>GCCGCGTCGGC<br>ACCGCTTCCGCGTCCCGTCCGGC	putative MarR-family transcriptional regulator
264	*sav_4550*	GCCGACGCCGACAGCC	putative GntR-family transcriptional regulator
265	*sav_4592*	ACCGACATCCGCC<br>GCCGCACTGCC	putative transcriptional regulator with cyclic nucleotide-binding domain
266	*sav_4647*	GCCGACGCCGACCCCGCC	putative regulatory protein
267	*sav_4648*	ACCGCGAAGTCTGCC	putative regulatory protein
268	*sav_4683*	GCCGCCGCCGATTGACAGGC	putative ArsR-family transcriptional regulator
269	*sav_4701*	GCCGCTGACCGTGCCGGCCTCTGCC	putative TetR-family

		GCCGACCGGC	transcriptional regulator
270	*sav_4762*	GCCGCGGCACGCCCTCCGCCCCGGC<br>GCCGCCTCGGC<br>GCCGCCGAGGCGGCGCCGCC<br>GCCGCCGAGGGGACTGCCGCC	putative AraC-family transcriptional regulator
271	*sav_4782*	GCCGCCCACCCGGC	putative TetR-family transcriptional regulator
272	*sav_4783*	ACCGCGCACACCCGGC	putative regulatory protein
273	*sav_4787*	ACCGCGTCCACCAACTCGGGCTGCC<br>ACCGCGCAGACCCGCACCTCTGCC<br>GCCGAAACCGGC	putative anti-sigma factor antagonist
274	*sav_4790*	GCCGAAGTCTCGGC<br>GCCGAGACTTCGGC	putative LysR-family transcriptional regulator
275	*sav_4816*	GCCGAACGGCGTGGCGCTGTCGCC<br>GCCGACGGACCGGC	putative TetR-family transcriptional regulator
276	*sav_4857*	ACCGCCGTTGGC<br>GCCGCTTCGCGGGGCGATCGCC	putative GntR-family transcriptional regulator
277	*sav_4878*	ACCGCCCCACACCGCC<br>ACCGCCCGGC<br>ACCGACCGACGCCCGCCCTGCC<br>ACCGATCAGCC<br>ACCGAAGATCCGTTATCGGC	putative two-component system response regulator
278	*sav_4879*	ACCGACACGGGCAGCC<br>GCCGACGCGCTCCATGGTCCGGC<br>ACCGCCCCACCCCGCCCGCTCCGGC<br>GCCGCGCGCCCGCC	putative two-component system sensor kinase
279	*sav_4880*	GCCGCGGCTCGCCGCCAGGC	putative two-component system sensor kinase
280	*sav_4978*	GCCGCCCCTGTAACCTTCCCGCC	putative LacI-family transcriptional regulator
281	*sav_4983*	ACCGCCGCAGGGGGTCCGCC<br>GCCGCCCCGTCCCCGGC	putative LacI-family transcriptional regulator
282	*sav_5014*	ACCGCCCTGCCTGCC<br>ACCGCCCGGC	putative regulatory protein
283	*sav_5030*	GCCGCCCGGC	putative transcriptional regulator
284	*sav_5032*	GCCGATACATCCGGC	putative transcriptional regulator
285	*sav_5033*	ACCGCGCAGGTCCGCC<br>GCCGCGCCCGTTCAGCC	putative transcriptional regulator
286	*sav_5034*	ACCGCGGGTCCGTCGGC<br>ACCGCGGGCCCGCC<br>GCCGACGGACCCGCGGTTCGCC<br>GCCGAACGGGTGAATCCCCGGC	putative transcriptional regulator

287	*sav_5068*	GCCGCACCGGC<br>ACCGATCCAGTTGTCAGCCGGC	putative two-component system response regulator
288	*sav_5084*	GCCGACAACAATGTTGGC<br>GCCGCAGTGTATCGGC	putative TetR-family transcriptional regulator
289	*sav_5220*	GCCGCCCCGGCCCGCCCTGCC	putative GntR-family transcriptional regulator
290	*sav_5311*	GCCGAACCGCCCCGTCCGACCGCC<br>ACCGCTCACGTACCGGC<br>ACCGCTCACGTACCGGC<br>ACCGCTTCGGC	putative acetyltransferase/MarR-family transcriptional regulator
291	*sav_5336*	GCCGCGCCGTATCCGGCCCGCCGGC<br>ACCGAGTGTGACCCGGGACCCGCC	putative MarR-family transcriptional regulator
292	*sav_5384*	GCCGCCTCTCGGC<br>GCCGAGCGCGTCTGCC<br>GCCGCGGGCTGCC	putative ROK-family transcriptional regulator
293	*sav_5522*	GCCGCCGCAGCC	putative cell-cycle regulator
294	*sav_5564*	ACCGAAGTGGGCCGCC	putative two-component system sensor kinase
295	*sav_5579*	ACCGACCGTCAATGGCGCCTCGCC	putative IclR-family transcriptional regulator
296	*sav_5623*	GCCGCCCGCC<br>ACCGCCGTCACCGCCGCC	putative two-component system sensor kinase
297	*sav_5644*	GCCGCCCCATGTCGGGTCGCC<br>ACCGCCCGATCCCGGCCGGC	putative MarR-family transcriptional regulator
298	*sav_5653*	GCCGAACCGCCTGGC	putative ROK-family transcriptional regulator
299	*sav_5700*	GCCGAGGCGGCCCCGGC<br>GCCGCCACCGGGCACCGTCCCGCC<br>GCCGCCTCGGC<br>GCCGAGAAGACTCGCCGCCGGC	putative transcriptional regulator with cyclic nucleotide-binding domain
300	*sav_5701*	GCCGCGGCCTCGGC	putative LacI-family transcriptional regulator
301	*sav_5719*	GCCGCGCCCCGACCCCGGTCCCGCC	putative two-component system sensor kinase
302	*sav_5724*	GCCGCCTGCC<br>GCCGCGGAAGCGCCCGGC	putative TetR-family transcriptional regulator
303	*sav_5740*	ACCGACTCCAGGAGCAGCC	putative regulatory protein
304	*sav_5741*	GCCGACAGCC<br>GCCGACAGCC<br>ACCGCAGGGCCATTGCCCGGC<br>ACCGCATACCTCTGACGGTCGGC	putative IclR-family transcriptional regulator
305	*sav_5746*	GCCGCTCTTCTGCC<br>GCCGCCCCGTTGTGCGACGCCCGCC	putative two-component system sensor kinase
306	*sav_5757*	ACCGAGCGCGGCGGTGCCGGC	putative transcriptional

		GCCGCGCTCGGTCTGGCGCTTCGGC	regulator
307	*sav_5792*	GCCGCATCGAGGGGTCGGGCTCGCC<br>GCCGCGGGCAGTCTCCGCC<br>GCCGCCCCTGTCCGCCACTTAGGC	putative MerR-family transcriptional regulator
308	*sav_5854*	ACCGCTCACACCGGCGCTACCTGCC	putative TetR-family transcriptional regulator
309	*sav_5869*	GCCGCCCCCGCCTCCCGGC	putative two-component system sensor kinase
310	*sav_5881*	GCCGCGGGATGTGGGCGGGCCGGC	putative AraC-family transcriptional regulator
311	*sav_5945*	GCCGCTGCCGGGCACCCGCC	putative TetR-family transcriptional regulator
312	*sav_5985*	ACCGATCGAATCCAGCC	putative TetR-family transcriptional regulator
313	*sav_5992*	ACCGAGCGGCCGCC	putative two-component system sensor kinase
314	*sav_6011*	GCCGATGTCCGTCAGCC<br>GCCGCGTGCCCGGATCGCC	putative regulatory protein
315	*sav_6021*	ACCGAGGGACACCTGCC	putative GntR-family transcriptional regulator
316	*sav_6038*	GCCGCGATTCCGGC<br>ACCGCCCGCATCAGCCCCGGC<br>ACCGCGGCCGGC<br>GCCGACGTCGCGCCTGCC	putative two-component system sensor kinase
317	*sav_6161*	GCCGCGCCGCGTCGGC	putative ArsR-family transcriptional regulator
318	*sav_6163*	GCCGCTGGCCGCATCGCCGCTCGCC<br>GCCGCCCGGC<br>GCCGCGTACGGTCCGGTCGGC<br>GCCGACTGGC	putative regulatory protein
319	*sav_6219*	ACCGCAGCTAGGTAGGC	putative two-component system response regulator
320	*sav_6252*	GCCGCGAACACTTGGC	putative regulatory protein
321	*sav_6288*	GCCGAATGAGATACGTTCCCGGC<br>GCCGACTCTGGCCGGC	putative LacI-family transcriptional regulator
322	*sav_6324*	ACCGCCCGGC	putative ArsR-family transcriptional regulator
323	*sav_6358*	ACCGAACGGGCCCGCCCGCC	putative DeoR-family transcriptional regulator
324	*sav_6476*	GCCGACCCCGCCGCC	putative two-component system sensor kinase
325	*sav_6536*	GCCGCTGTCCGCAAGCGCTGGC	putative DeoR-family transcriptional regulator
326	*sav_6598*	GCCGCCCCTGGCCCTGGC	putative ArsR-family

		GCCGAGTTCCCGCCGCCCGGC	transcriptional regulator
327	*sav_6701*	ACCGACCCGACCCGCC<br>ACCGACCGGC	putative TetR-family transcriptional regulator
328	*sav_6724*	GCCGAGAGCCATAAGGTATTGCC	putative GntR-family transcriptional regulator
329	*sav_6745*	GCCGACCCGCC<br>ACCGACGCCCCAGCC<br>ACCGCGCCGTCCGCCCGGCTGCC<br>GCCGACAGAGTAGTGCGTCCGCC	putative transcriptional regulator
330	*sav_6758*	GCCGACTGACGCCGGACAGGC<br>ACCGCACCGCCGCGGCGAACCGGC	putative MerR-family transcriptional regulator
331	*sav_6889*	ACCGAGCCGGTGTTACCCCAGGC<br>ACCGCAATGGTTGCC	putative sensor-like histidine kinase
332	*sav_6901*	GCCGCTGTGCCGCC	putative ROK-family transcriptional regulator
333	*sav_6917*	ACCGCGTCCGGTTCGCC<br>GCCGATGTCCCGCTGGC	putative TetR-family transcriptional regulator
334	*sav_6921*	GCCGCAGTGCCTTGTCGGCCCAGCC<br>GCCGACGGCCGGCTGCCGGCCGCC	putative AsnC-family transcriptional regulator
335	*sav_6954*	ACCGCACGTCCCTGACTGCC	putative IclR-family transcriptional regulator
336	*sav_6994*	GCCGCCCGCGGTCGCCGGC<br>GCCGCGTGCCCCCGCGTCTCGGC<br>ACCGCCCGCC<br>ACCGCCCTGGC<br>ACCGCCTGTCAGCC	putative PadR-family transcriptional regulator
337	*sav_7025*	ACCGATTGGTCCTGGC	putative transcriptional regulator
338	*sav_7044*	ACCGCGTCCACTCCCCGCCCGGC	putative ArsR-family transcriptional regulator
339	*sav_7061*	GCCGCTGTTCAGGCTTCCCGGC	putative AsnC-family transcriptional regulator
340	*sav_7070*	GCCGCGCCGAGTACGATCCGGC<br>GCCGCCGGGCCGCC	putative ROK-family transcriptional regulator
341	*sav_7097*	GCCGACTCCTTTCGCC<br>ACCGAGGCCGCC	putative regulatory protein
342	*sav_7178*	GCCGCATCGCCGCCGCATCGCC	putative transcriptional regulator
343	*sav_7235*	GCCGCCCCGCGCCCCCGCCCGGC<br>GCCGCTGAGCCGTCCCACCGCC	putative LysR-family transcriptional regulator
344	*sav_7331*	ACCGCACCCGAAGAACGCCCGGC	putative LuxR-family transcriptional regulator
345	*sav_7346*	ACCGAACGGTAACCGCCCGGC	putative LacI-family transcriptional regulator

346	*sav_7354*		GCCGATCCGGC	putative MarR-family transcriptional regulator
347	*sav_7422*		GCCGCGCCGGC	putative LacI-family transcriptional regulator
348	*sav_7452*		GCCGAGGGGAGCCGTTCGGC<br>GCCGATCAGTTCCGCC<br>GCCGAACGGCTCCCCTCGGC	putative anti-sigma factor antagonist
349	*sav_7510*		GCCGATGTGTTCGCC	putative TetR-family transcriptional regulator
350	*sav_7514*		ACCGCTGGCAAGATCTGCC	putative WhiB-family transcriptional regulator
**Secondary metabolism (84)**				
351	*sav_936*	*aveF*	GCCGCGCGGGACGTCTCCGCC<br>GCCGCCGCACCGGC<br>ACCGCCACGGCGGCCGCC<br>ACCGCCGCCATCATGACCGCC<br>GCCGACGCCATGGATCCGGC	C-5 ketoreductase
352	*sav_937*	*aveD*	ACCGCTAGGCAATGCTCGGC<br>GCCGCCCGGC<br>GCCGCCGAGCATTGCC	C5-O-methyltransferase
353	*sav_938*	*aveA1*	GCCGCCGAGCATTGCC<br>GCCGCCCGGC<br>ACCGCTAGGCAATGCTCGGC	type I polyketide synthase AVES 1
354	*sav_942*	*aveA3*	GCCGACTGGCCGACCGGC	type I polyketide synthase AVES 3
355	*sav_943*	*aveA4*	GCCGATCCGAGAGCGCAGGC<br>ACCGCCTGCGCTCTCGGATCGGC	type I polyketide synthase AVES 4
356	*sav_944*	*orf-1*	ACCGCCTGCGCTCTCGGATCGGC<br>GCCGATCCGAGAGCGCAGGC	reductase
357	*sav_2892*	*olmA4*	GCCGAATGCTAGGC<br>GCCGCGTCCCCTCAGCC	modular polyketide synthase
358	*sav_2899*	*olmA1*	ACCGCCCCTAGCC<br>ACCGCTATCGGC	modular polyketide synthase
359	*sav_2900*		GCCGAATCGGC<br>GCCGACGAAGTCGCGGGCGCCGGC<br>GCCGCCACACGGGTCACCACCGCC<br>GCCGCCCCTAGCC<br>GCCGATTCGGC	putative P450-like protein
360	*sav_2902*	*olmRI*	ACCGAGAAATGTCACAGCC<br>ACCGCAACCGCGCAGGC	LuxR-family transcriptional regulator
361	*sav_2903*	*olmC*	GCCGACGGGTCACAGCC<br>GCCGCCGCGACCCGGC	thioesterase
362	*sav_840*		ACCGCGTGCTCGGCGCCCTCGCC<br>GCCGAGCACGCGGTGCGGGCCGCC	putative thioesterase

363	*sav_849*		ACCGCTGCGTCCAGGGCGTCGCC<br>GCCGCCAGCGCAGCGGGTGCCGGC<br>GCCGCCACCGGC<br>ACCGCGTGGCCACCGCC<br>GCCGACCGGC	putative methyltransferase
364	*sav_851*		GCCGCCGACACCGGC<br>GCCGCCCAGGC	putative MbtH-like protein
365	*sav_856*		GCCGACGCACGACCGCC<br>ACCGCGGCGATCGCTCCCTTGGC<br>ACCGCCTACGTCCGGGCCGCCCGGC<br>ACCGCACGCCTCGCC	putative thioesterase
366	*sav_857*	*nrps7-9*	GCCGCCACCGTAGGC	putative non-ribosomal peptide synthetase
367	*sav_859*	*nrps7-10*	GCCGCGAGGTCGGC	putative non-ribosomal peptide synthetase
368	*sav_865*	*nrps7-12*	ACCGACACCGGTCGGC	putative non-ribosomal peptide synthetase
369	*sav_1019*	*crtU*	GCCGCACGTCGCGGCAGGGACCGGC<br>GCCGCGACGTGCGGCGGCCGGC	beta-carotene desaturase/methylase
370	*sav_1021*	*crtY*	GCCGCCGCACCGGGCCGGC<br>GCCGCCGGAGTCAGCC<br>GCCGCCCACCACCCCGTCCCGGC<br>GCCGCCCACCATCCCGTCGGC<br>GCCGCCCACCACCCCGTCGGC<br>GCCGAACGACCCGGCCGGC	lycopene cyclase
371	*sav_1022*	*crtE*	GCCGAACGACCCGGCCGGC<br>GCCGCCCACCACCCCGTCGGC<br>GCCGCCCACCATCCCGTCGGC<br>GCCGCCCACCACCCCGTCCCGGC<br>GCCGCCGGAGTCAGCC<br>GCCGCCGCACCGGGCCGGC	geranylgeranyl diphosphate synthase
372	*sav_1249*		GCCGCGGGCGTCCCGCC<br>GCCGCACCCGCCCCCGGC<br>GCCGCGATGCCGCC	putative modular polyketide synthase
373	*sav_1251*		GCCGAACCGCC	putative hydroxylase
374	*sav_3199*	*nrps1-3*	GCCGCCGACCCAGGCCGCCCCGCC<br>ACCGATGCCCGTGGCGGCCGGC	putative non-ribosomal peptide synthetase
375	*sav_3648*	*cysK2*	GCCGCCCCGGCCTGCC	putative cysteine synthase
376	*sav_3651*	*nrps2-4*	GCCGCATCCCCCGGCCGCC<br>ACCGCACAACGGCCGCC<br>GCCGCCCGGCCCGGC	putative non-ribosomal peptide synthetase/acyl-CoA dehydrogenase fusion protein
377	*sav_6633*	*nrps5*	GCCGCCGACCAGCACGTCAGCC	putative non-ribosomal peptide synthetase
378	*sav_3644*		ACCGATCACCGACCAAGGAGTTGCC	putative MbtH-like protein

379	*sav_7161*		ACCGCTGGTACGCGCGGTCGGC	putative regulatory protein
380	*sav_7165*	*nrps4*	GCCGATCGCC<br>GCCGCTCGTCGCCGTCCCGTCGGC<br>GCCGCGAGGACTGCC<br>GCCGCCGGACGCCTGCC	putative non-ribosomal peptide synthetase
381	*sav_1551*	*pks2-2*	GCCGCCGTCGTACCGGC<br>ACCGCTTCAGCCGGACCGGC<br>ACCGCCGCCGGC<br>GCCGACGCCGGTCCGGC	putative modular polyketide synthase
382	*sav_1552*	*pks2-3*	GCCGACCTGGCCCCCTTGCC	putative non-ribosomal peptide synthetase
383	*sav_1553*		GCCGACCTGGCCCCCTTGCC	putative methyltransferase
384	*sav_2276*	*fabH7*	ACCGCCTCCGGCACCGGCCGCC<br>ACCGCCCGGCCGCC	putative 3-oxoacyl-ACP synthase III
385	*sav_2281*	*pks3-2*	ACCGACCAGGC	putative modular polyketide synthase
386	*sav_2282*	*pks3-3*	GCCGCGGCCGGGGCCTGGC	putative acyl carrier protein
387	*sav_2366*		GCCGCAATGTCGCTGCCGGC<br>GCCGACGGCCAGCAGGCCGGC	putative hydroxylase
388	*sav_2368*	*pks5*	GCCGATCCCTTTGCCGGGCAGGC	putative modular polyketide synthase
389	*sav_2369*		GCCGATCCCTTTGCCGGGCAGGC	putative regulatory protein
390	*sav_2377*	*cyp10*	GCCGCATGCTGTCTCGGC<br>ACCGCTGCCGCGAGCATCGGC	putative cytochrome P450
391	*sav_2382*		GCCGCACCGGC<br>ACCGCCCGGC	putative O-methyltransferase
392	*sav_2386*		GCCGCCGCGCTCATCGGC	putative ClpX homolog
393	*sav_2387*	*pks9-7*	ACCGCGCCGCGCCGGCCAGCCGGC<br>GCCGCCATCGCACCCGGGTAGGC	putative 3-oxoacyl-ACP reductase
394	*sav_3657*	*pks8-2*	GCCGAGACCTTCGCC	putative 3-oxoacyl-ACP synthase I
395	*sav_3662*		GCCGCCACCCGGCCGCCCACCGCC	putative hydrolase
396	*sav_3666*	*pks8-9*	GCCGCGTATAGCAGGC	putative acyl carrier protein
397	*sav_7184*	*pks4*	ACCGACCGCAGCC<br>GCCGACCCTGGACGGGGCCGCC	putative modular polyketide synthase
398	*sav_7185*		GCCGCCGCTGCGGGTTGTCGGC	putative UDP-glucose:sterol glucosyltransferase
399	*sav_7360*	*pks1-1*	ACCGCGGTCGGC	putative acyl carrier protein
400	*sav_7362*	*pks1-3*	ACCGCCCTCGGGTCAGGC<br>GCCGCGCGCGTCCTCCGCC	putative modular polyketide synthase
401	*sav_1136*	*melC1*	GCCGCACCACTCGCC<br>GCCGCCCCGGGTGGCCCACCCGGC	tyrosinase co-factor protein
402	*sav_5361*	*melC1-2*	GCCGCCGACCTGCCGCC<br>ACCGAAGGAGGCCGGTCCCTCCGCC	putative tyrosinase co-factor protein

			ACCGCAGGCCCGGC<br>GCCGCTGTGTTCACCGCC<br>GCCGCGGACACATGGAGTCGGC	
403	*sav_2835*	*sppF*	GCCGATCGCC	putative polyketide hydroxylase
404	*sav_2836*	*sppG*	GCCGATCGCC	putative WhiE I homolog
405	*sav_2837*	*sppH*	GCCGACGGCGTAGTGCGCGTCCGGC	putative WhiE II homolog
406	*sav_2842*	*sppE*	GCCGCGGACCGCC	putative cyclase II
407	*sav_5274*	*sidF*	GCCGCGGGGCGCCCGCC<br>GCCGCACCTGTGAGCAGCC	iron(III)/siderophore uptake ABC transporter substrate-binding protein
408	*sav_5689*	*mcjC2*	ACCGACCGGGAGGACGCGACCGCC<br>GCCGATTCACTGCC<br>ACCGCCCCTCCTGGCCGGC	putative asparagine synthase
409	*sav_7584*	*mcjA1*	ACCGAGTGAGTCTCCTCACCGGC	putative microcin mature peptide
410	*sav_76*	*ams*	GCCGCGTTCTCCCGGC<br>GCCGAGAAGGCCGCAATTGCCGCC<br>GCCGCCGGGCGGGTCGGC<br>GCCGCCCGGCCGGCTGCGTCTGCC<br>GCCGACCCGCCCGGC<br>GCCGCGACGGCGCGTGCCGGC	avermitilol synthase (sesquiterpene cyclase)
411	*sav_1650*	*hopA*	GCCGCGAGCAGAGAGAAGCACTGGC	squalene-hopene cyclase
412	*sav_1652*	*hopC*	ACCGCGGCGGACCGGC<br>GCCGCTGGAATCACCTTGGC	squalene/phytoene dehydrogenase
413	*sav_1654*	*hopE*	GCCGAAGCGTTCCGGC<br>ACCGATTGGC	squalene/phytoene synthase
414	*sav_2163*	*geoA*	GCCGCAGTTCCGCGCCTGCCTGCC	germacradienol/geosmin synthase
415	*sav_2989*	*ptlR*	ACCGACAACGCCCCGGC	putative MarR-family transcriptional regulator
416	*sav_2991*	*ptlH*	GCCGATATCAGCCGCCGGC<br>GCCGCACACATGCGGAGTCCGGC<br>GCCGCGCCCATGTGTGATGACCGGC<br>GCCGACTGGC	1-deoxypentalenic acid 11-beta hydroxylase; Fe(II)/alpha-ketoglutarate dependent hydroxylase
417	*sav_2993*	*ptlF*	GCCGCTGCCCGAGCCGCGCCGGC<br>ACCGCTCGCC	1-deoxy-11beta-hydroxypentalenic acid dehydrogenase
418	*sav_2996*	*ptlC*	ACCGCCTCGGC<br>GCCGACCTGGC	hypothetical protein
419	*sav_2998*	*ptlA*	GCCGCCCGCC<br>ACCGCAGGCAGCGCCGCC<br>ACCGCCCTCCCCACCGCAGGC<br>GCCGCCTGCTCCCGGC<br>ACCGCTCAGGCCGCC	pentalenene synthase

			GCCGCGCCCGGCCCGGCCGGC<br>GCCGAGCCGGC	
420	*sav_3032*	*ezs*	ACCGCTTTTTCTGGC	epi-isozizaene synthase (sesquiterpene cyclase)
421	*sav_408*	*pteG*	GCCGCTCACGCAGTGCCCGGC<br>GCCGCGTCTTCCCCTGCCCGGC<br>ACCGAACCCCCGGGCCGGC<br>ACCGCATGGAGACCGCGCACAGGC	putative cholesterol oxidase
422	*sav_409*	*pteF*	GCCGCTCGGC<br>ACCGCGAACGCGTCAGCC<br>GCCGAACACCTGCCGCTTTCTTGCC<br>GCCGCTGGCGACCCCTTGCC	putative LuxR-family transcriptional regulator
423	*sav_410*	*pteR*	ACCGCGAACGCGTCAGCC<br>GCCGCTCGGCCCGCCACGCCGGC	DnrI/RedD/AfsR-family transcriptional regulator
424	*sav_412*	*pteD*	GCCGCCGTGGCCCTCCCGCC<br>GCCGCGACCCTCGCACCGACTGCC<br>GCCGCTTGCC<br>ACCGAGGCCGCC<br>ACCGAAACCTGTTTCCCGTAGCC<br>ACCGCGCGCCGGC	cytochrome P450 hydroxylase
425	*sav_419*	*pteA1*	GCCGCCTGGTCGAGCCGATCGCC<br>GCCGAGAATCACTACCGACCTCGCC<br>GCCGCTACACGGGCGATCGGC	modular polyketide synthase
426	*sav_7320*	*avsA*	GCCGCGTCGAGCGGATGCCCGCC<br>GCCGAGGCCACCGGC	putative siderophore synthetase component
427	*sav_7322*	*avsC*	GCCGCGACCGCC	putative siderophore synthetase component
428	*sav_1457*	*axe2*	ACCGATAATTTCCGGC	putative acetyl xylan esterase
429	*sav_2563*	*dxr*	ACCGACAGTCCAGCCCCCCTCGCC	putative 1-deoxy-D-xylulose 5-phosphate reductoisomerase
430	*sav_3210*	*ispH*	GCCGAACCGCCGGC<br>GCCGCGTTGTCACAGGC<br>ACCGACCCGGC<br>GCCGAAGATCGCC<br>GCCGCACCCGGC	putative 4-hydroxy-3-methylbut-2-enyl diphosphate reductase
431	*sav_3706*	*aco*	ACCGCCGAAGACAGGC	putative acyl-CoA oxidase
432	*sav_3905*	*mqnB*	ACCGCGGTCCCGGC	putative futalosine hydrolase (menaquinone biosynthetic protein)
433	*sav_6616*		GCCGCCCGCC<br>GCCGCCGACTGACAGGC	putative polyketide cyclase
434	*sav_7363*		GCCGCGCGGTACCCGGC<br>GCCGATTCCCGCGCGTCGGC<br>GCCGAGCCCGTGGTCCCCACCGCC	putative carboxylesterase

			GCCGCTGAGGTCCGGC<br>GCCGAGTACTGGC<br>GCCGACGCGCGGGAATCGGC	

Metabolism of amino acids and related molecules (100)

435	*sav_4967*	*alr*	GCCGCTCGCC	putative alanine racemase
436	*sav_7269*	*amdA1*	GCCGCCTGTCCCCAGCGCCTGCC<br>GCCGCTCTCGACGTGGCGCCGCC<br>ACCGCACCTCAGGC<br>ACCGCTCGCGGGCCGGACGGCAGGC	putative amidase
437	*sav_7345*	*amdA2*	ACCGAACGGTAACCGCCCGGC	putative amidase
438	*sav_1790*	*arcB1*	GCCGCGCGGCCGGCCGGC<br>GCCGCCGGCGGCCGGC	putative ornithine cyclodeaminase
439	*sav_6763*	*argC*	ACCGACGCTGCCCGCC<br>ACCGCAGGCTGCC<br>ACCGAGTGCCGGC	putative N-acetyl-gamma-glutamyl-phosphate reductase
440	*sav_2319*	*argF*	GCCGCCCCGGCCGGC	putative ornithine carbamoyltransferase
441	*sav_801*	*argG1*	GCCGCTGGAACACCGGC<br>ACCGCCCGCACCGACCTGCGCAGCC<br>GCCGATGCCGACCCCGCC<br>GCCGCAGGGTCGAAGAAGCTCGCC	putative argininosuccinate synthase
442	*sav_6778*	*argG2*	GCCGAACGCAGGC	putative argininosuccinate synthase
443	*sav_3042*	*aroA*	GCCGCTTTCCGCC	putative 3-phosphoshikimate 1-carboxyvinyltransferase
444	*sav_6854*	*aroC*	GCCGCCGCAGCCGCCGCGCAGGC<br>GCCGCAGCGGCCCGGTCTTCTGCC<br>GCCGCCGCGCCCCATCGCC	putative chorismate synthase
445	*sav_1777*	*aroE2*	GCCGCCGGACGGGTCGCC	putative shikimate 5-dehydrogenase
446	*sav_6210*	*aroH2*	GCCGCACCCCGGGATGTCCCGCC	putative chorismate mutase
447	*sav_5397*	*asd2*	GCCGCAGGCGCCGGAAACGTTCGGC<br>GCCGCCCGGCCGCC<br>GCCGAACGTTTCCGGC	putative aspartate-semialdehyde dehydrogenase
448	*sav_3128*	*aspB2*	GCCGCACGGGACCGAGGCGTTTGGC	putative aspartate aminotransferase
449	*sav_4907*	*aspC1*	ACCGCATCCGCC	putative aspartate aminotransferase
450	*sav_6399*	*aspC2*	GCCGCGGCGGCGGGATCGGC<br>GCCGATCCCGCCGCC	putative aspartate aminotransferase
451	*sav_5253*	*bglC2*	ACCGCCGCCCCGGC<br>GCCGCGGAGGTCTCGGC	putative beta-glucosidase
452	*sav_6404*	*cobC*	ACCGATCTCGCC	putative aminotransferase
453	*sav_1435*	*cysK1*	ACCGCGCGACGCGGCGTCGGC	cysteine synthase/cystathionine

				beta-synthase family
454	*sav_3510*	*cysM1*	GCCGCCGCCACCTCCTTGTAGCC<br>GCCGACCGCC	putative cystathionine beta-synthase
455	*sav_2516*	*dapA2*	GCCGCAATACGGACACTTCGCC	putative dihydrodipicolinate synthase
456	*sav_5580*	*dapA3*	ACCGACCGTCAATGGCGCCTCGCC	putative dihydrodipicolinate synthase
457	*sav_4517*	*dapC*	GCCGCTCCTCCGGC<br>GCCGCCGCTGTCGCCGCCCGCC<br>ACCGCAGCCCGGCCTTCTTGTTGCC	putative N-succinyldiaminopimelate aminotransferase
458	*sav_6339*	*dapD*	GCCGCCCGTGCTCGGC	putative 2,3,4,5-tetrahydropyridine-2-carboxylate N-succinyltransferase
459	*sav_3125*	*dapE*	GCCGAGCGGGAGTGGGGTCGGC	putative succinyl-diaminopimelate desuccinylase
460	*sav_2473*	*dapF1*	GCCGCGTGATCGGATCCGCC<br>ACCGACGGCCCGGC<br>GCCGCCTGCC<br>GCCGATCCGCCTGCC<br>ACCGACACCCCACGGCCGCC<br>ACCGCTCGCC	putative diaminopimelate epimerase
461	*sav_1690*	*ddh*	GCCGCAGGCCCCCCGTCGCAGGC	putative dimethylarginine dimethylaminohydrolase
462	*sav_7126*	*def2*	GCCGCGCCCTGTCCCGGC	putative polypeptide deformylase
463	*sav_7349*	*def3*	GCCGCTCGATCTGCCTGGC	putative polypeptide deformylase
464	*sav_6397*	*ectB*	ACCGAGGAGCGATTTCGCC	L-2,4-diaminobutyrate aminotransferase
465	*sav_6395*	*ectD*	GCCGCGGCGCCGCC	ectoine hydroxylase (Fe (II)/alpha-ketogultarate dependent hydroxylase)
466	*sav_7341*	*egtA*	ACCGAAGCCGGGTCCGGC	putative glutamate-cysteine ligase
467	*sav_7134*	*gabD2*	GCCGACCACCGGCCGACCACCGGC<br>GCCGACCACCGGC	putative succinate-semialdehyde dehydrogenase, NADP-dependent
468	*sav_7159*	*gabD3*	GCCGCCACCTACCGCC<br>ACCGACCGTCTCACCGGC<br>GCCGAAGCCGTCCGCC	putative succinate-semialdehyde dehydrogenase, NADP-dependent
469	*sav_2773*	*gcvT*	GCCGCATCTGTAACCGGC	putative glycine cleavage

				system protein T (aminomethyltransferase)
470	*sav_5075*	*gdhA1*	GCCGATGGCGTTGCC	putative NAD-specific glutamate dehydrogenase
471	*sav_4963*	*glmS1*	GCCGCTCCACGCTCACTCCCGGC<br>GCCGAGCCGTACCCCGGC<br>GCCGAGAGCCGCC	putative L-glutamine-D-fructose-6-phos phate amidotransferase
472	*sav_5954*	*glnA1*	GCCGACCTCGGC	putative glutamine synthetase
473	*sav_5997*	*glnA2*	ACCGCGGCCGGC<br>ACCGCGGCGTTCGGC	putative glutamine synthetase
474	*sav_6005*	*glnA3*	ACCGCACTGGC<br>GCCGAAGTTAACCGGC	putative glutamine synthetase
475	*sav_6725*	*glnA4*	GCCGAGAGCCATAAGGTATTGCC	putative glutamine synthetase
476	*sav_1314*	*glsA*	GCCGAGGGCCTGTTCGCC<br>GCCGACCGGC	putative glutaminase
477	*sav_6189*	*gltB*	GCCGCACTCAGCC<br>GCCGCAGCCCGGCGATGCCCTCGCC<br>GCCGATGCGTACGCCGCGCCAGCC<br>GCCGCGGCTGAGTGCGGCGCAGGC<br>GCCGAGGGACGACGTTGGC	putative glutamate synthase (NADPH) large subunit
478	*sav_6258*	*gltD2*	ACCGCGCCACGAGGCTCTCGGC<br>GCCGCCTGGC<br>ACCGACCGGGAACCGTGCCAGGC<br>GCCGCCCGGCCCGCCGCTCGGC	putative glutamate synthase small subunit
479	*sav_3419*	*glyA3*	GCCGCGTCGCCGCCCGTCCGGC<br>GCCGACGGGCCGTGACTGGC	putative serine hydroxymethyltransferase
480	*sav_6955*	*hcaC2*	ACCGCACGTCCCTGACTGCC	putative ferredoxin subunit of phenylpropionate dioxygenase
481	*sav_4261*	*hisC2*	ACCGCATGAGCCCTACTATCGGC<br>GCCGAGCAGGC<br>ACCGCCACCGGC<br>ACCGACTGGC	putative histidinol-phosphate aminotransferase
482	*sav_6153*	*hisD*	GCCGAGCAACCGAGCAGCC	putative histidinol dehydrgenase
483	*sav_6906*	*hisE*	GCCGAGCCGGC	putative phosphoribosyl-ATP pyrophosphatase
484	*sav_6170*	*hisI*	ACCGCCCGGCCGGTCGGC<br>GCCGCGCGGTCCGCC<br>GCCGACCGGCCGGGCGGTCGTAGGC<br>GCCGCGCCGGTCGGACATCCCCGGC	putative phosphoribosyl-AMP cyclohydrolase
485	*sav_3325*	*hutH1*	GCCGCCCGGTCCCGCC	putative histidine ammonia-lyase
486	*sav_3504*	*hutH2*	ACCGCCAGGC	putative histidine ammonia-lyase

487	*sav_2733*	*ilvB1*	ACCGCACCCGGC	acetolactate synthase large subunit
488	*sav_2731*	*ilvC*	GCCGACGACTGACGACCGCC	ketol-acid reductoisomerase
489	*sav_4716*	*ilvD1*	ACCGCCACCGACCGGC<br>ACCGCCACCGACGGTCCGCC<br>ACCGCCCCCGACCGGC	putative dihydroxy-acid dehydratase
490	*sav_2717*	*ilvE*	GCCGCGCCACTGCC<br>GCCGCACGCACACCGTTCACAGCC<br>GCCGCGCTCACGCCCGTACCGCC	putative branched-chain amino acid aminotransferase
491	*sav_5601*	*leuA2*	ACCGCCAGCAGCC	putative 2-isopropylmalate synthase
492	*sav_2686*	*leuC*	GCCGACACAGGCCGGC<br>ACCGCGCGTCCGCCGCC	putative 3-isopropylmalate dehydratase large subunit
493	*sav_4559*	*lysC*	GCCGCTCCAGGACGGCATCTGCC<br>GCCGAAATTCCCTGCC<br>ACCGCACAACTGCGGTCGGC	putative aspartate kinase
494	*sav_3382*	*mcmA2*	ACCGCCCCCTACTCCGCC<br>ACCGCACATCTGCC<br>GCCGACTCGTTGGTTACTCGCC	putative methylmalonyl-CoA mutase, alpha subunit
495	*sav_3305*	*metB*	GCCGCGCGCGGCACGTACAGCC<br>ACCGCCCGGC<br>GCCGCACCCGTAGGC<br>ACCGCCGACCGGGTTGGC<br>GCCGCAGGCCGGGCGGTTTTGCC	putative cystathionine gamma-synthase
496	*sav_2046*	*metE*	ACCGCGCCTTGCCGCGCGGGTCGGC<br>GCCGACCCGCC	putative 5-methyltetrahydropteroyltriglutamate-homocysteine methyltransferase
497	*sav_6667*	*metH*	ACCGCCCCGTTCGGC<br>GCCGAGCCATTGGC	putative 5-methyltetrahydrofolate: homocysteine S-methyltransferase
498	*sav_7062*	*mgl*	GCCGCTGTTCAGGCTTCCCGGC	putative methionine gamma lyase
499	*sav_2111*	*mmuM*	GCCGCGGCCGAGAGGAGGGATCGCC<br>GCCGCGGCCCAGCCGTACTGGC	putative homocysteine S-methyltransferase
500	*sav_3307*	*msrA*	GCCGCAGCACCACCACGTCGGC<br>GCCGCGTAGCC<br>ACCGCCCCGGCCTCAGGGTGTAGGC<br>ACCGACCGCC<br>GCCGCTGCTGCC<br>ACCGCGAGGAGCGCACCGACCTGCC	putative peptide methionine sulfoxide reductase
501	*sav_2330*	*narB*	GCCGACGACGGTACGAATTGCC	putative assimilatory nitrate reductase large subunit

502	*sav_2127*	*nirA*	GCCGAGGTCGACGTGCCGCC	putative ferredoxin-nitrite reductase
503	*sav_1257*	*paaK*	GCCGACCTCTTGCC<br>ACCGAATGATCGGTCGCC<br>ACCGATCATTCGGTAGCC	putative phenylacetate: CoA ligase
504	*sav_5473*	*proA*	ACCGCGAGACAGGC	putative gamma-glutamyl phosphate reductase
505	*sav_2823*	*pta*	GCCGCGAAAATCACCCGCC	putative phosphotransacetylase
506	*sav_3420*	*rocD1*	GCCGACGGGCCGTGACTGGC<br>GCCGCGTCGCCGCCCGTCCGGC	putative ornithine aminotransferase
507	*sav_2285*	*rocD3*	ACCGCGGGCCGTGAGCGGCCGCC	putative ornithine aminotransferase
508	*sav_1636*	*rocG*	GCCGACCGCC	putative NADP-specific glutamate dehydrogenase
509	*sav_5053*	*sahH*	GCCGCATCCGCC<br>GCCGCCCGGC<br>GCCGCGCCGCC<br>GCCGCCGCGCAGACCAGCC<br>GCCGCGCGGACGCCGGGCGGCTGCC<br>ACCGCCATCAGCAGCGACGTCTGGC	putative S-adenosyl-L-homocysteine hydrolase
510	*sav_4736*	*serB1*	GCCGCAGAGAGTTCACAGCC<br>ACCGAACAAACTGGC	putative 3-phosphoserine phosphatase
511	*sav_6470*	*serB2*	GCCGATCCGGC<br>GCCGCCGCTGGGGGCCGACCCGGC<br>GCCGAGGGCCGACCCGGCTGGC<br>GCCGCGCCCCCGGACCGGATCGGC<br>GCCGCGCCCGCGAGCCGGATCGGC<br>GCCGCGCCCGCGGACCGGGCCGGC<br>GCCGCGCCAGCCGGGTCGGC	putative 3-phosphoserine phosphatase
512	*sav_3883*	*serC*	ACCGAGCAGGTCGGC<br>GCCGCCCGCCGCCCCGGC	putative phosphoserine aminotransferase
513	*sav_1628*	*tdh*	GCCGAGGCGGGCCGGACAGCC	putative threonine 3-dehydrogenase
514	*sav_4526*	*tdo*	GCCGCTCAGCC	putative tryptophan 2,3-dioxygenase
515	*sav_2918*	*thrA*	GCCGCCGTACAGCTGCTGCC<br>ACCGAACGGGACTTTCCGGC	putative homoserine dehydrogenase
516	*sav_2916*	*thrB*	GCCGCCTGAACCGCTCGAACCGCC<br>ACCGCCCACCCCTACCGCC	putative homoserine kinase
517	*sav_6177*	*trpB*	GCCGCTGCCGCTGCCGGC<br>GCCGCCCGGC<br>GCCGAACCGGC<br>GCCGCTCTGCC<br>GCCGCCCGCTCCCGCCGTCGGC	putative tryptophan synthase beta subunit

518	*sav_7104*	*ureA*	GCCGCGGTGGCTGCGGCAGCC	putative urease gamma subunit
519	*sav_2716*	*ureAB*	ACCGACAGGGCCTCGCC<br>GCCGACCTCGCC	putative urease beta/gamma subunit
520	*sav_954*	*yerD1*	GCCGAAGTCACCCGGACCCGGC<br>GCCGCAGGCCACAGCGGCAGCC<br>ACCGCACTGGC<br>GCCGAAGGCCGGC<br>GCCGCCCTCCATGTCCGCC	putative glutamate synthase (ferredoxin)
521	*sav_1232*	*yerD2*	GCCGCCCCTCAGCCGGC	putative glutamate synthase (ferredoxin)
522	*sav_1061*		GCCGCGGCGCGTGGGAGAGGCCGGC	putative aminotransferase
523	*sav_1112*		ACCGCACGCCGGGCGCCCGGC	putative taurine catabolism dioxygenase
524	*sav_1520*		ACCGCACACGGCCGACCGCC<br>GCCGCTGGATGGGGCGACAGCC<br>GCCGCTCGGC	putative 1-aminocyclopropane-1-carboxylate deaminase
525	*sav_2578*		ACCGCAGCCGACGGCTCTTCCGCC<br>ACCGCTTCCGCTCAGCC	putative sugar hydrolase
526	*sav_2592*		GCCGACCCCCGCC	putative aldehyde dehydrogenase
527	*sav_2692*		GCCGCCCCCACCGCGTTAGCC	putative 2-keto-4-pentenoate hydratase/2-oxohepta-3-ene-1,7-dioic acid hydratase
528	*sav_3009*		GCCGCCCGGCCGCC	putative 1-aminocyclopropane-1-carboxylate deaminase
529	*sav_3281*		GCCGCGCGACAGCGAGGAGCCCGGC	putative D-amino acid deaminase
530	*sav_4551*		GCCGCGGACATCTACCCTCGGC	putative aminotransferase
531	*sav_6046*		ACCGACCAGCGAGGCCGCAGCC<br>ACCGCGCGCTCGGC	putative aminotransferase
532	*sav_6057*		GCCGACTGTCCGGC<br>GCCGAGCCCCGCC<br>ACCGCGCCCCGCGTCACCTCGCC	putative aminotransferase
533	*sav_6946*		ACCGCCCGCAGACCGGC	putative choline oxidase
534	*sav_7160*		ACCGCTGGTACGCGCGGTCGGC	putative aminotransferase
**Carbohydrate metabolism (114)**				
535	*sav_1115*	*abfB*	GCCGACAGGC<br>GCCGACCTGGCATTACAGGC	putative alpha L-arabinofuranosidase
536	*sav_2043*	*aceA*	GCCGCACCCCAGAGACAGGC<br>GCCGCACCGAAACTCTGACACTGGC	putative isocitrate lyase
537	*sav_2000*	*aceB1*	ACCGCTCCCGAGGCGCCCCCAGCC	putative malate synthase
538	*sav_2044*	*aceB2*	GCCGCGTCCACCGGC<br>GCCGCCCGCATCGCCCGGCCCGCC	putative malate synthase

539	*sav_5800*	*aceE1*	GCCGAAACGGTGACGATCCTGGC<br>GCCGACACCGCC	putative pyruvate dehydrogenase E1 component
540	*sav_6020*	*aceE2*	GCCGCCCCGCCGCC<br>GCCGCCCTGTCCTGCC	putative pyruvate dehydrogenase E1 component
541	*sav_2258*	*acnA*	ACCGCGGTCCGGGCATGACCAGCC<br>GCCGAAGGCGCCCCGCAGCCGCC<br>ACCGCGGTGACCTGCC<br>ACCGCAATCCTGCCGCC	putative aconitase
542	*sav_4599*	*acsA1*	GCCGCCCGGACCGCACTCGGCCGCC<br>ACCGCACCCAGCGTCCCGGC<br>GCCGCGCCTGGC	putative acetyl-CoA synthetase
543	*sav_6970*	*acsA2*	GCCGCGTGCCGCGCACACCCCAGCC	putative acetyl-CoA synthetase
544	*sav_1141*	*adhA1*	GCCGATGCCTGGC	putative alcohol dehydrogenase
545	*sav_1357*	*adhA2*	GCCGACCCGTCGCC	putative alcohol dehydrogenase
546	*sav_1393*	*adhA3*	GCCGATTACACAGGCCGCC	putative alcohol dehydrogenase
547	*sav_3314*	*adhA4*	ACCGCTGGACGTAGGC<br>GCCGAAGGCAGCCGGC	putative NADP-dependent alcohol dehydrogenase
548	*sav_3707*	*adhA5*	ACCGCTGCCTGCC<br>GCCGCCCGAGGTCGGCGTCGTCGGC<br>GCCGCGTGGCAGGCAGCGGTCAGCC<br>GCCGCCCAGCC	putative alcohol dehydrogenase
549	*sav_6953*	*adhA10*	ACCGAGCCATACAGGCCTGGC<br>GCCGCTGTGTGTCGGC<br>GCCGAAATCTCGCC	putative alcohol dehydrogenase
550	*sav_2186*	*agaA2*	GCCGAGGGTCTGGC<br>ACCGCGCCGACATCACGCCGCC<br>GCCGCCGCTCACTTGACTGCC	putative alpha-galactosidase, secreted
551	*sav_1077*	*agaA3*	GCCGCCGTCCGACCGCC<br>GCCGCCCCGCACCAACCGCC	putative alpha-galactosidase, secreted
552	*sav_1641*	*agaB2*	GCCGCGCAAGAGATTCCGCC	putative alpha-galactosidase, secreted
553	*sav_1187*	*aldH*	GCCGCATTGCC	putative aldehyde dehydrogenase
554	*sav_2152*	*amyA1*	GCCGATCCGTCCTGACCGGC<br>GCCGCCGGGGGCGTCCGGC<br>GCCGACGGTACGTCTCGCC<br>GCCGAAGCCGCCCGGC<br>GCCGCCAGGGCCCGCTCGCC	putative alpha-amylase
555	*sav_5756*	*bga7*	ACCGCCGCGCTCGGTCTGGC<br>ACCGAGCGCGGCGGTGCCGGC	putative beta-galactosidase
556	*sav_7479*	*bga8*	GCCGCGGGCCGGC<br>GCCGCGGTGGCGATCCGGC	putative beta-galactosidase, secreted
557	*sav_4362*	*bkdA*	GCCGCCCGCCCGCC<br>ACCGATTGTTCGGTCGTCGGC	putative 3-methyl-2-oxobutanoate

				dehydrogenase (lipoamide) E1-alpha chain
558	*sav_4363*	*bkdB*	GCCGATGACCTCCGTTGGC	putative 3-methyl-2-oxobutanoate dehydrogenase (lipoamide) E1-beta chain
559	*sav_1856*	*celA3*	GCCGCCCGGC<br>ACCGCCCGGC	putative secreted endo-1, 4-beta-glucanase
560	*sav_2568*	*celA4*	GCCGCCGGATCTTCCGGAGGTTGCC	putative secreted endo-1, 4-beta-glucanase
561	*sav_6896*	*celA5*	GCCGCCCGCAGGGCCGAGCGCAGGC	putative endo-1, 4-beta-glucanase, secreted
562	*sav_5568*	*celS1*	GCCGCCCGCTCCCGGC<br>GCCGCGGACCGGCGGCGGCCGCC<br>ACCGCCCTGCC<br>GCCGACCGCCGCC<br>GCCGCCACCTCGCCCACCGCCGGC<br>GCCGAGCGGGGCCGGGGGCCCGCC	putative cellulose-binding protein
563	*sav_2254*	*celS2*	GCCGCACCCGCC<br>GCCGCCGCTCCTGGGGCCGGC	putative cellulose-binding protein
564	*sav_2585*	*chiA1*	ACCGCCTCAGATCGCC	putative secreted chitinase A
565	*sav_1751*	*chiD*	GCCGATGCTGCCGCC<br>GCCGCTGGTCAGCAGCC	putative secreted chitinase I
566	*sav_5330*	*citA1*	GCCGCCAATAGGC<br>GCCGCCAACCTGCTCATCCTGCC	putative citrate synthase
567	*sav_5219*	*cslA*	ACCGACCTGCC	putative glycosyltransferase (cellulose synthase-like protein)
568	*sav_5217*	*cslZ*	ACCGACAAGGGTTGATTGTCGCC	putative endo-1, 4-beta-glucanase, secreted
569	*sav_2015*	*csn1*	GCCGCCCCCGGCCGCC	putative chitosanase, secreted
570	*sav_1307*	*dexA*	GCCGCGAAGATGCTCGCC	putative oligo-1, 6-glucosidase
571	*sav_3533*	*eno*	GCCGATCCCTCAGAGCCGGTTAGGC	putative enolase
572	*sav_3942*	*fruK*	GCCGACCGCCGTCTCCTTTGGC<br>ACCGCTGCCGACAGGC<br>GCCGACCGCC	putative tagatose-6-phosphate kinase
573	*sav_6421*	*fucA2*	GCCGCACACGTCGCC<br>ACCGATACATATCGCC<br>GCCGCGATCCTGCCAGGC	putative fuculose-1-phosphate aldolase
574	*sav_4575*	*galE2*	ACCGCGCTGCC<br>GCCGCACGCTAGGCCCCGGC	putative UDP-glucose 4-epimerase
575	*sav_1011*	*galE5*	ACCGCACCGCGTCCGGC	putative UDP-glucose 4-epimerase
576	*sav_1662*	*galE6*	ACCGCAGATCGCC	putative UDP-glucose

			ACCGCCTCCGCC GCCGACCGGC ACCGCCATCCTCACCGGTCAGCC GCCGACACGCCGCCCGGCCGCC GCCGCGGTTTGTGGCCCGGC	4-epimerase
577	*sav_3018*	*gamA*	ACCGCACCCGGC GCCGATGTGGGACAGGC	putative glucosamine-6-phosphate isomerase
578	*sav_6296*	*gap2*	ACCGAAACGAGTGGTCAGGC ACCGCCTCGCC	glyceraldehyde-3-phosphate dehydrogenase (sensitive to pentalenolactone)
579	*sav_1922*	*gdh*	GCCGCGCCGTCCCGGGCAACCCGGC	putative glucose 1-dehydrogenase
580	*sav_2024*	*gip*	GCCGCCGTTTTCCACCGGC GCCGAAGCCCGGC	putative hydroxypyruvate isomerase
581	*sav_2805*	*glgB*	GCCGACCCTGCC	putative 1, 4-alpha glucan branching enzyme
582	*sav_2800*	*glgP*	GCCGACACGCCACTGGGCAGCC ACCGCCACAAGGTCGCGCAGGC GCCGCGGTGAATTTCTGCC ACCGCACGAGAGGCTGCC GCCGATTCCGCCCGCC GCCGCGGGCCATCGGGTGCCCGCC	putative glycogen phosphorylase
583	*sav_1918*	*glxK*	ACCGCAGGACGCACTCGCC	putative glycerate kinase
584	*sav_4318*	*gnd2*	GCCGCCGGGCGCGACGGGGTCCGGC	putative 6-phosphogluconate dehydrogenase
585	*sav_7249*	*gnd3*	GCCGCTCCATCCCGCC GCCGATTGCCGCCTTGTCCTGGC	putative 6-phosphogluconate dehydrogenase
586	*sav_3979*	*gpmA1*	GCCGACAGGCGGACGCCCCTGCC	putative 2, 3-bisphosphoglycerate-depende nt phosphoglycerate mutase
587	*sav_7214*	*icdA*	GCCGACGGTCTCCCCGCCCGCCGGC GCCGACGGTCTCCCCCGCC GCCGCCCGTCTCCGCC GCCGATCGACGCCCCCGCC GCCGACCTCGCCGGGCCGGC GCCGCCCTGATCGGCTCGGC	isocitrate dehydrogenase
588	*sav_7150*	*iolC2*	ACCGCACCAGGC GCCGCGTCATCGGC	5-dehydro-2-deoxygluconokina se
589	*sav_1234*	*iolG1*	ACCGCTGCTCCGCCGCCCCGCC	putative myo-inositol 2-dehydrogenase
590	*sav_3280*	*kdgK*	GCCGCGCGACAGCGAGGAGCCCGGC	putative 2-oxo-3-deoxygluconate kinase
591	*sav_4877*	*korA*	ACCGCTCCCGGACAGCCGCC	putative 2-oxoglutarate

				ferredoxin oxidoreductase,<br>alpha subunit
592	*sav_1764*	*lamA1*	GCCGAGGTTCTGGTAGATGCTGGC<br>GCCGCTGGGCCGCCTGGGGCCGCC	putative endo-1,<br>3-beta-glucanase, secreted
593	*sav_6024*	*lpdA1*	GCCGACCGGTCACTGACGACCGGC<br>GCCGCTCTTCTTCGGC	putative dihydrolipoamide<br>dehydrogenase
594	*sav_3340*	*lpdA3*	GCCGCCGTTTCGCTGCC	putative dihydrolipoamide<br>dehydrogenase
595	*sav_7348*	*lpdB*	GCCGAAGGCCGGCACCGGACCGCC<br>GCCGAGCGCCAGGGTGCTCTCCGCC<br>GCCGCCCCGGCCGAGCGGCCGCC<br>GCCGAGAGACGGCTTTGCCGCC<br>ACCGCCCCCTCGGC<br>GCCGCGGCCGCC<br>GCCGACCGCC<br>GCCGCTCGGC<br>GCCGCCGGACGGCCCCGGGCTGCC<br>GCCGCGGCCCATTACCGGC	putative dihydrolipoamide<br>dehydrogenase
596	*sav_2981*	*maeB1*	ACCGAAGATCTTCCGGC<br>GCCGACGAGATGTCTCCTGGC<br>ACCGCGTAGGCCCAGCCGCC	putative malate dehydrogenase<br>(oxaloacetate-decarboxylating;<br>NADP-requiring)
597	*sav_5126*	*maeB2*	ACCGACCCGCCATGCTCGCC<br>GCCGAAGGCCGCCGGGCCGCC<br>ACCGCGGAGTACGCGGTAGCCGCC	putative malate dehydrogenase<br>(oxaloacetate-decarboxylating;<br>NADP-requiring)
598	*sav_1514*	*malS1*	ACCGATCGCC<br>ACCGCACTTCAGGC<br>ACCGAAGGTGACCGGGGACTCGCC	putative malate dehydrogenase<br>(oxaloacetate-decarboxylating;<br>NAD-requiring)
599	*sav_3870*	*malS2*	GCCGATACCGGCCGGTACCGGC<br>ACCGCCCGCCGCC<br>GCCGATATCAGTCGTTACCTGCC	putative malate dehydrogenase<br>(oxaloacetate-decarboxylating;<br>NAD-requiring)
600	*sav_5048*	*manB*	ACCGCGCCGGTCCCGGC<br>GCCGCTTCGGC<br>GCCGAGGCACCGCC<br>GCCGCGCCGGCCTACCGCGACCGGC<br>ACCGCCTCCTTGCC<br>GCCGAAACGGCCGCGCCGGC<br>GCCGCGTCCTTACCGGATCGGC<br>ACCGACAGTCGGCGGTGCCTCGGC	putative phosphomannomutase
601	*sav_3436*	*mdh*	GCCGCCGAGCCAGCC<br>GCCGCCCGCACCCTGGGCAGGC	putative malate/lactate<br>dehydrogenase
602	*sav_358*	*mpg1*	ACCGACCGACAACGGAGTCCGCC	putative mannose-1-phosphate<br>guanyltransferase
603	*sav_6977*	*mpg3*	GCCGCCCAATCAGGC	putative mannose-1-phosphate<br>guanyltransferase

604	*sav_1842*	*neuA1*	GCCGAGACCTGGC<br>ACCGCCGCCGCCTGGGGAACCGGC	putative neuraminidase, secreted
605	*sav_5934*	*neuA3*	GCCGCAACCGGC	putative neuraminidase
606	*sav_2822*	*pfkA1*	GCCGCGAAAATCACCCGCC	6-phosphofructokinase
607	*sav_6083*	*pfkA2*	GCCGCGGTTGCC	6-phosphofructokinase
608	*sav_803*	*pgmA*	GCCGCGGAACCGTCCCCCTCGCC<br>GCCGAGTTGAGAGAGGACCCGCC<br>GCCGACCTTCCGGCTGCC<br>GCCGACCACGGGGTGCCTCTCGCC<br>GCCGAACTGTCCGCC<br>ACCGCACGGCTGCCATGCCGCAGCC	putative phosphoglucomutase
609	*sav_3343*	*pmmB*	GCCGCCGCGGGACGCGTGCCGCC	putative phosphomannomutase
610	*sav_3566*	*ppc*	GCCGCCCGGC<br>GCCGCCGGGCGGCGCACTGCC	putative phosphoenolpyruvate carboxylase
611	*sav_2825*	*pykA1*	ACCGATAGGATCGCC	putative pyruvate kinase
612	*sav_5317*	*rbsK*	GCCGCTCGGC	putative ribokinase
613	*sav_5705*	*scrK1*	GCCGCGTCGCC<br>GCCGAGAGAGAGCAGCC	putative fructokinase
614	*sav_3395*	*sdhC1*	ACCGACATCGAAGCCGGCAGGC<br>ACCGATGTGATCGAACTGGC	putative succinate dehydrogenase cytochrome b-556 subunit (complex II)
615	*sav_1818*	*sucC1*	ACCGCTCCGGC<br>ACCGATGGCTTTCGGCCGCC	putative succinyl-CoA synthetase beta subunit
616	*sav_372*	*suhB2*	ACCGCACAGTCCGGC	putative myo-inositol 1-monophosphatase
617	*sav_6314*	*tal2*	ACCGCGACCGCC<br>GCCGCACGGCCGGCACGCGTCGGC<br>GCCGACGCGTGCCGGC	putative transaldolase
618	*sav_7177*	*thcA*	ACCGCGCCCCGCC<br>GCCGAGAGCTGCC	putative aldehyde dehydrogenase
619	*sav_1766*	*tkt1*	ACCGACAGCAGGGCCGAGCCGCC	putative transketolase
620	*sav_6315*	*tkt2*	GCCGACCTCCGGATCAGCC<br>GCCGATGTGCTTGGCGCCCACCGGC	putative transketolase
621	*sav_4462*	*wbpA*	GCCGCGAGCTGCC<br>ACCGCGTCACCGGC	UDP-glucose/GDP-mannose dehydrogenase
622	*sav_1126*	*xylB2*	GCCGCACGGCGAACCGGC	putative xylulose kinase
623	*sav_5283*	*xylB3*	GCCGACAACACCCGGC<br>ACCGCCGCCTCTGCC<br>GCCGCACCGCCGCTTCTGCC<br>GCCGCACCACCACCCCAGCC<br>GCCGCACCGCCACCCCAGCCCGCC<br>GCCGCGCCGCACCGCCCCCACCGGC<br>GCCGCCCCGCCGGGTGTTGTCGGC<br>GCCGCCCGCTTGCC	putative xylulose kinase

624	*sav_2096*	*xynA1*	GCCGCGCACAGGCCTGCC<br>GCCGCCGGGCGCGGGCAGGTTGCC	putative endo-1,4-beta xylanase, secreted
625	*sav_4984*	*xynA2*	GCCGCTTCCGTATTCGCC<br>ACCGAAACTTTCGGAGGCACCCGCC<br>ACCGATTGGC	putative endo-1,4-beta xylanase, secreted
626	*sav_6372*	*xynB3*	GCCGATTCCCGCCGATACTGTTGCC<br>ACCGCACTGCTCGGC<br>GCCGCTCTGGC	putative xylan beta-1, 4-xylosidase
627	*sav_6381*	*xynD*	GCCGCCCCGGC<br>GCCGCCACTGCCGGAAAGGGTTGCC	putative endo-1,4-beta-xylanase, secreted
628	*sav_828*		ACCGCCGAATCGGACAGCC<br>GCCGCGCCCGCGCCCACGTCTGCC<br>ACCGCCGTCCGCCTCGCC<br>GCCGATTCCAGCACACTCGCC<br>GCCGACGATCAGGCAGCC	putative rhamnosidase
629	*sav_1324*		GCCGCCGGGACCGGC	putative arabinogalactan endo-1, 4-beta-galactosidase, secreted
630	*sav_1405*		GCCGACGGCACGAGCCTCGCC<br>GCCGCACCTCACGCACCGGCCGCC<br>GCCGCGTGCAGGGGCCGCCCGGC	putative amidohydrolase
631	*sav_1459*		GCCGAGCCGGCACAGGC<br>GCCGCCTCAGCC	putative glycogen debranching enzyme
632	*sav_1623*		ACCGCACTCCGGC<br>GCCGAGGCGCCGGC	putative transketolase A subunit
633	*sav_1624*		GCCGCGCCCGGTCCGTCGGC<br>ACCGCACAGCACTCGCCGCCCGCC	putative transketolase B subunit
634	*sav_1980*		GCCGCGGCGTGAGGCAGAATCGGC<br>GCCGATTCTGCC	putative carbohydrate kinase
635	*sav_2009*		GCCGCAGGTCCGGC	putative mutase
636	*sav_2087*		GCCGCCGGTCGCAACCGGCCGCC<br>ACCGAGAGTTGCGTAACCGCC<br>ACCGAGCCGGGAGGGGGCCTGCC<br>ACCGCCCGGC	putative cellulose-binding protein
637	*sav_2108*		GCCGCTAGCGGTACGGTCCAGCC	putative rhamnogalacturonase B precursor, secreted
638	*sav_2109*		GCCGACCGCC<br>GCCGCATGAGCCGCACAAGCCGCC<br>GCCGCCCGGC<br>ACCGCGATTTTCTGCTATCGGC	putative beta-xylosidase, secreted
639	*sav_2574*		GCCGCACGATGTCACAGCC<br>GCCGCGTGGGCGCGACCAGCC	putative secreted endo-1, 4-beta-glucanase
640	*sav_2804*		ACCGACCCGCC	putative pep2 protein

641	*sav_3050*		ACCGAAGGGCGTGCGCCGGC<br>GCCGAAGGGTGCGCCGGC<br>GCCGCTCGCC<br>GCCGATCTTCGCCGGC	putative fructose-1,<br>6-bisphosphatase
642	*sav_3483*		ACCGAACGCCCCCCACCGTCCGCC	putative phosphoglycerate mutase
643	*sav_3908*		ACCGCCCAGGATGGGCACCGCC	putative hydrolase
644	*sav_3924*		GCCGCGGGTCGCC	putative sugar phosphate isomerase
645	*sav_4650*		ACCGACGTCGATCGGGTGTCCGCC	putative aldehyde dehydrogenase
646	*sav_4732*		GCCGCGAGAGCGCGTTCTGCCGGC<br>GCCGAACGGGAACTCGTCCGGC<br>ACCGATGTAGGC	putative NAD-dependent epimerase/dehydratase
647	*sav_5245*		GCCGAGATCGCCCGCCGGGTTGCC	putative mutase
648	*sav_6969*		GCCGCGTGCCGCGCACACCCCAGCC	putative glycosyl hydrolase
**Fatty acid and lipid metabolism (75)**				
649	*sav_3337*	*accA3*	ACCGCGGGACAAGAGGTCTGCC<br>GCCGAGTTTAGGGACTGCC	putative acyl-CoA carboxylase, alpha subunit
650	*sav_3331*	*accD4*	GCCGAAAGGTATCGCCGCC	putative acyl-CoA carboxylase, beta subunit
651	*sav_4964*	*acpS*	ACCGCGGCGCCCCCGCC	putative holo-ACP synthase
652	*sav_2890*	*ccrA1*	ACCGAGCGGTCCCCAGGATCCCGGC	crotonyl-CoA reductase
653	*sav_1911*	*ccrA2*	GCCGCGTGTCCCCACCGCC	putative crotonyl-CoA reductase
654	*sav_524*	*cdh*	ACCGAGGCTGATTTCCGGC<br>ACCGACTGCC<br>ACCGCATGCCCTCTTCGGC	putative CDP-diacylglycerol phosphotidylhydrolase
655	*sav_3528*	*cfa*	ACCGAACAGCC	putative cyclopropane fatty acid synthase
656	*sav_1064*	*clsA*	GCCGCCGACGCTTCCGCC<br>GCCGCCCCTCCACCACCGGTCGGC<br>ACCGCGCGAGCGCAAACCTGGC	putative phospholipase
657	*sav_6102*	*crtI2*	GCCGATAAAGTCCCTGGC	putative phytoene dehydrogenase
658	*sav_1285*	*dak1*	GCCGAACAACGAGTTCGGC<br>GCCGATACTGCC<br>GCCGAACTCGTTGTTCGGC	putative dihydroxyacetone kinase subunit I
659	*sav_2674*	*dak3*	ACCGCGGCCGGCCCGCGCCCTTGCC<br>GCCGCCGGGGTGGTCCGGC<br>GCCGCTGGCAAGGGCGCGGGCCGGC<br>GCCGCAAGAGCGCCGCGAGACCGCC	putative dihydroxyacetone kinase
660	*sav_635*	*echA2*	ACCGAGCTGGAATCGAACATCTGCC<br>GCCGAAGCTCATCGCC	putative enoyl-CoA hydratase

661	*sav_2316*	*echA7*	GCCGCGCCCGGCTGTGCCCGCC<br>ACCGACAGCCGCCCCCGCC<br>ACCGAGTCACGGCTTCTCGATAGCC<br>ACCGCGCGATAGCTGTGTCCCGGC	putative enoyl-CoA hydratase
662	*sav_3327*	*echA9*	GCCGATGCCGCGGGCGTCGGC<br>GCCGCCCCCTCCGGTGTCGCC<br>GCCGCCCGGATGCCGCC<br>GCCGACGCCCGCGGCATCGGC<br>ACCGACCGCGAGCCACCGGC<br>GCCGACCGCGAGCCACCGGC	putative enoyl-CoA hydratase
663	*sav_6428*	*echA14*	ACCGAAGCCGATGGACCGCGCCGGC	putative enoyl-CoA hydratase/isomerase
664	*sav_4544*	*eutB*	GCCGCCCGGC	putative ethanolamine ammonia-lyase large subunit
665	*sav_2944*	*fabB2*	GCCGCGCCGGTCGGC<br>GCCGACAGGCGACCGTGACCTGCC<br>ACCGACGTGACGACCGGAATCCGGC<br>GCCGACCGGC	putative 3-oxoacyl-ACP synthase II
666	*sav_6136*	*fabG6*	ACCGCCCGCTTCCAGCC	putative 3-oxoacyl-ACP reductase
667	*sav_6462*	*fabG7*	GCCGCCCCGGC	putative 3-oxoacyl-ACP reductase
668	*sav_1831*	*fabH2*	GCCGACAAGCCTGCCGCC<br>GCCGATCAGGGCCCGGC	putative 3-oxoacyl-ACP synthase III
669	*sav_827*	*fabH6*	GCCGACACCGGCCGCC<br>ACCGACGGCGACCCGGC	putative 3-oxoacyl-ACP synthase III
670	*sav_3512*	*fadA2*	GCCGCCCCCGGCGCCCGCTTCGGC<br>GCCGCCCCCGGC	putative 3-ketoacyl-CoA thiolase/acetyl-CoA acetyltransferase
671	*sav_1681*	*fadA4*	ACCGCGCCCGGCAGAGCCCGGC	putative 3-ketoacyl-CoA thiolase/acetyl-CoA acetyltransferase
672	*sav_1384*	*fadA5*	ACCGCGAGCATGCCCACCCCGGC<br>GCCGCGCCGCAGTTGGC<br>ACCGCCTACCGGCATCGAGCCCGGC<br>GCCGCCTGCCCCTGGAGACCGGC<br>GCCGCCATTGCC<br>GCCGCAGGGGTGACGGGAGTCGGC	putative 3-ketoacyl-CoA thiolase/acetyl-CoA acetyltransferase
673	*sav_7026*	*fadA6*	ACCGATTGGTCCTGGC	putative 3-ketoacyl-CoA thiolase/acetyl-CoA acetyltransferase
674	*sav_5842*	*fadA7*	GCCGCGCCCCTTGCC<br>GCCGCAAGGGTCTTGCAGCC	putative 5-ketoacyl-CoA thiolase/acetyl-CoA acetyltransferase

675	*sav_1909*	*fadC1*	ACCGACCGGC	putative 3-hydroxyacyl-CoA dehydrogenase
676	*sav_2870*	*fadC3*	ACCGCTAAGGTCCGCC	putative 3-hydroxyacyl-CoA dehydrogenase
677	*sav_2889*	*fadC4*	ACCGAGGAGTCCGCCGCC	putative 3-hydroxyacyl-CoA dehydrogenase
678	*sav_6748*	*fadC5*	ACCGAACAACGGGGGCCCTGCC	putative 3-hydroxyacyl-CoA dehydrogenase
679	*sav_716*	*fadC6*	GCCGCCGCAGCC	putative 3-hydroxyacyl-CoA dehydrogenase
680	*sav_377*	*fadD1*	ACCGCCTCCACGAACGTCGCC	putative acyl-CoA synthetase, long-chain fatty acid:CoA ligase
681	*sav_1104*	*fadD3*	GCCGCGTAGCCGGACAGCC<br>GCCGAGGGTAGGC	putative acyl-CoA synthetase, fatty acid: CoA ligase
682	*sav_1258*	*fadD5*	GCCGAACCGCC<br>GCCGAACCGCC<br>GCCGATGACTCCGGTCGCTCCGGC<br>GCCGCCCCCTTCCGCCACCGTAGCC	putative acyl-CoA synthetase, long-chain fatty acid: CoA ligase
683	*sav_1848*	*fadD8*	ACCGCTCATGCTCCCTCACTGCC	putative acyl-CoA synthetase, long-chain fatty acid:CoA ligase
684	*sav_4206*	*fadD9*	ACCGCGTAGGC<br>ACCGCAGGACGAACAGCC	putative acyl-CoA synthetase, long-chain fatty acid:CoA ligase
685	*sav_4818*	*fadD10*	GCCGCGGGCGACACCGGC<br>ACCGCCGGTGTCGCC	putative acyl-CoA synthetase, long-chain fatty acid:CoA ligase
686	*sav_5562*	*fadD12*	GCCGCAACCAGCCAGCC	putative acyl-CoA synthetase, long-chain fatty acid:CoA ligase
687	*sav_3806*	*fadD16*	GCCGACAGTGCTGGC	putative acyl-CoA synthetase, fatty acid: CoA ligase
688	*sav_3841*	*fadD17*	ACCGAGCCTTACGCTCTGCC	putative acyl-CoA synthetase, fatty acid: CoA ligase
689	*sav_6919*	*fadE2*	GCCGCTGGTCAGCAGCC<br>GCCGATGCTGCCGCC	putative acyl-CoA dehydrogenase
690	*sav_6542*	*fadE12*	ACCGCTGTCCCGCC	putative acyl-CoA dehydrogenase
691	*sav_1267*	*fadE14*	GCCGACGCCGCC	putative acyl-CoA dehydrogenase
692	*sav_1381*	*fadE15*	GCCGCATCGGC<br>GCCGATGCGGCGCCAGGGCCGCC<br>GCCGATGGCACCGCC	putative acyl-CoA dehydrogenase

693	*sav_4212*	*fadE22*	GCCGATGCGCTCCCATCGGC<br>GCCGATGGGAGCGCATCGGC<br>GCCGAAGCACCGCC	putative acyl-CoA dehydrogenase
694	*sav_2123*	*fadE23*	GCCGCGAGCCCTGGCCTACCGGC<br>ACCGCCTGGC	putative acyl-CoA dehydrogenase
695	*sav_1292*	*fadH*	GCCGATGCCGGGGCCGGCGGCCGGC<br>GCCGCCGTGGCCGCCCCTGCC<br>ACCGCGCACCAGACCGGC<br>GCCGCCGGCCCCGGCATCGGC	putative 2, 4-dienoyl-CoA reductase
696	*sav_2298*	*fadS2*	GCCGATCAGCC<br>GCCGCCCGCGTTCCGTCACCGCC<br>GCCGCGGGGGGCTTGCCGCC	putative fatty acid desaturase
697	*sav_3567*	*fadS3*	ACCGCATAGGC<br>GCCGCAGGGGGCAACCGCC	putative fatty acid desaturase
698	*sav_719*	*gcdH1*	GCCGCGCAGCGATGCTGTTGTCGCC	putative glutaryl-CoA dehydrogenase
699	*sav_6664*	*glpK1*	ACCGCGCCGTAGATACAGCC	putative glycerol kinase
700	*sav_4224*	*glpQ2*	ACCGAGAACAACAGCC<br>GCCGCGGACCCACAGCC	putative glycerophosphoryl diester phosphodiesterase
701	*sav_6784*	*glpQ3*	GCCGCGCCCCCGAACCCGGC	putative glycerophosphoryl diester phosphodiesterase
702	*sav_1492*	*glpQ5*	GCCGAGCCGCC	putative glycerophosphoryl diester phosphodiesterase
703	*sav_3460*	*icmB*	GCCGCGAAACTGCC<br>GCCGCACCACTCCGCCGCC	isobutyryl-CoA mutase, chain B
704	*sav_7089*	*lpsA1*	GCCGCATCGGTGAAATCGCC	putative secreted lipase
705	*sav_3807*	*ltp2*	GCCGACAGTGCTGGC	putative nonspecific lipid-transfer protein
706	*sav_1682*	*mcr*	ACCGAGGTGTGCGTCATCCTCGGC<br>ACCGCTCGCACCTCACGCTAGCC	putative fatty acid-CoA racemase
707	*sav_1604*	*pcaF1*	ACCGACGGCGGGCGGTGCACCTGCC<br>ACCGCCCGCC	putative beta-ketoadipyl-CoA thiolase
708	*sav_6826*	*pgsA2*	ACCGCGGGCGTCATCGGAGCCGCC	putative phosphatidylglycerophosphate synthase
709	*sav_6976*	*pgsA3*	GCCGAACTTCTTTTAGAGCTCCGGC	putative CDP-diacylglycerol-glycerol-3-phosphate 3-phosphatidyl-transferase
710	*sav_1658*	*pgsA5*	GCCGCTCCGCC	putative phosphatidylglycerophosphate synthase
711	*sav_2681*	*plsC2*	GCCGCACAAGTGTCGCCCCAGGC	putative 1-acylglycerol-3-phosphate

				O-acyltransferase
712	*sav_6080*	*plsC3*	ACCGACCAGGGTCGTCGGC	putative 1-acylglycerol-3-phosphate O-acyltransferase
713	*sav_6783*	*plsC4*	GCCGCGCCCCCGAACCCGGC	putative 1-acylglycerol-4-phosphate O-acyltransferase
714	*sav_7311*	*plsC7*	GCCGCGACCCCCGCTCCCCGGC<br>GCCGCCCGGC<br>ACCGACGACCGCGGCTGTGCCGGC<br>GCCGCGGTCGTCGGTCGGACCGGC<br>GCCGCGGCCGTCGGC<br>ACCGACACGGGACCAGGAACCGGC	putative 1-acylglycerol-3-phosphate O-acyltransferase
715	*sav_1916*	*psd*	GCCGCCGACTCGGCTTCTTGGC<br>GCCGAGTCGGC	putative phosphatidylserine decarboxylase
716	*sav_3237*	*uppS1*	ACCGACGTCAGGCCGCCCGTCGCC<br>ACCGCCATACGGACAGGC<br>ACCGCCATCCGGACAGGGATCGCC<br>ACCGCCGAAACGCCCGGC	putative undecaprenyl diphosphate synthase
717	*sav_5629*	*uppS2*	GCCGATGCGCGGGCGCCGTAGGC	putative undecaprenyl diphosphate synthase
718	*sav_1346*		GCCGACCCGGAGCACATCGCTCGCC<br>ACCGACCCCCGCGCCGGC<br>GCCGCGCACAAGGACCGGC<br>ACCGCGCCGGC	putative acyl-CoA synthetase
719	*sav_2155*		GCCGCGGAGGGAGCCGGACCGCC	putative 3-hydroxyacyl-CoA dehydrogenase
720	*sav_2388*		GCCGCCCTGGCCGCC<br>GCCGAATTGCC	putative phosphopantetheinyl transferase
721	*sav_3120*		GCCGCCGCACGGACTTCTGCC	putative enoyl-CoA hydratase/isomerase
722	*sav_3461*		GCCGCTACGAGGTAGCC<br>GCCGCACACGCACCGTCGGC<br>GCCGACGGGAGGTCGGC<br>GCCGACCTCCCGTCGGC	putative lipase
723	*sav_6182*		ACCGCGGCGCGGGCAGCC<br>ACCGACGGGCAGCC	putative acyl-CoA transferases/carnitine dehydratase
**Nucleotide metabolism (34)**				
724	*sav_2595*	*add2*	ACCGCGTCCGCC<br>ACCGATCAGCC<br>ACCGCAGCCCGGC<br>GCCGAACCCGTGGGCACCCCCGGC	putative adenosine deaminase
725	*sav_6768*	*apbE2*	GCCGACGGGCCGGC	putative thiamine biosynthesis

				lipoprotein precursor
726	*sav_4180*	*cdd3*	GCCGCGGCATGACGCCCTGCC<br>ACCGCCCGGCGGCCGCC<br>GCCGAGGGGACATGGCCCTCGCC<br>GCCGCGGCCGCGGGGTCCGGC<br>GCCGCTCGGC	putative cytidine/deoxycytidine deaminase
727	*sav_4806*	*cdd4*	GCCGCCGCCCGCCGCC	putative cytidine/deoxycytidine deaminase
728	*sav_1247*	*cpdA*	GCCGCCGGGCCTTTGCC<br>GCCGCGTCGGC	putative cyclic nucleotide phosphodiesterase
729	*sav_4464*	*dcd*	GCCGACAGGCTCGGCCCCGGCCGGC<br>GCCGAGCCTGTCGGC<br>GCCGCTGCTACCGCTGCTGCC	putative 2'-deoxycytidine 5'-triphosphate deaminase
730	*sav_3365*	*deoA*	GCCGCACAGGC	putative thymidine phosphorylase
731	*sav_3342*	*deoD*	GCCGCCTGACCGCC<br>GCCGCGCTCCGTGGGACTTCTCGCC	putative purine nucleoside phosphorylase
732	*sav_1949*	*dht*	ACCGACCGCC<br>GCCGCCCGTGCACCGGACCGGC	putative dihydropyrimidinase
733	*sav_5649*	*dus*	GCCGCGAGGCGCCCGGC	putative dihydrouridine synthase
734	*sav_3479*	*guaA*	ACCGCGCGCCCGCAGGCCCGCC	putative GMP synthase
735	*sav_5000*	*guaB1*	GCCGAGCGGCAGCC<br>GCCGCTCGGC<br>GCCGCCCCGGC<br>ACCGCCTCACTGCTCGCC	putative inosine-5'-monophosphate dehydrogenase
736	*sav_6823*	*hit*	GCCGCAGAGGTTCGCC	putative histidine triad (HIT) family protein
737	*sav_6267*	*indA*	GCCGCCCGCC	putative pigment biosynthetic protein
738	*sav_3679*	*mtaP*	GCCGAAACACCGAAGACCCCGCC	putative methylthioadenosine phosphorylase
739	*sav_3026*	*nrdL*	GCCGCCGGGAAATCCGGC<br>GCCGACCGGC<br>GCCGCCGGATTTCCCGGC	putative ribonucleoside-diphosphate reductase alpha chain
740	*sav_3530*	*ppx1*	GCCGCGCCCCCAGGC<br>GCCGATCGCCGCC	putative exopolyphosphatase
741	*sav_3562*	*prsA1*	GCCGCGCGAACTCGCTGCTCGCC	putative ribose-phosphate pyrophosphokinase
742	*sav_7506*	*pucA*	GCCGCGAGCGGCGCCGGATCGCC	putative xanthine dehydrogenase
743	*sav_1540*	*pucE*	ACCGATCGCCCACCGGC<br>ACCGCCCCTTCGGC	putative xanthine dehydrogenase
744	*sav_1996*	*pucH*	ACCGACCGACAGGTAGGC	putative allantoinase

745	*sav_5021*	*purK*	ACCGACAGGGGCTGGC	phosphoribosylaminoimidazole carboxylase ATPase subunit
746	*sav_3445*	*purN*	GCCGACCGGCAGCAACGATACCGGC	phosphoribosylglycinamide formyltransferase
747	*sav_4140*	*purS*	GCCGATACTTTCCCAATTACAGCC	putative phosphoribosylformylglycinamidine (FGAM) synthase, PurS component
748	*sav_3846*	*purU*	GCCGACTGCGTAGCC	putative formyltetrahydrofolate deformylase
749	*sav_4522*	*pyrE*	GCCGCGCGGCGCCGTCTACCCTGGC	putative orotate phosphoribosyltransferase
750	*sav_6504*	*pyrG*	GCCGCTCCCGGC	putative CTP synthetase
751	*sav_6840*	*relA*	GCCGCAGTGGCCCTCCCGGACAGGC	ppGpp synthetase
752	*sav_2472*	*rshA*	GCCGCATTCGGC<br>GCCGCAATGTCTTACGGCCCGGC<br>GCCGACCGTGGATTCGAACCGGC<br>ACCGCCTCTCGCC	putative RelA homolog
753	*sav_2517*	*thyX*	GCCGAATTCGCAGAAGTTCGGC<br>GCCGAACTTCTGCGAATTCGGC	putative thymidylate synthase
754	*sav_4622*	*tmk*	GCCGCCCCTCCGGGGAGGCCCCGCC	putative thymidylate kinase
755	*sav_1953*		GCCGCACGGCCGGC<br>GCCGCTCCTTTGCC	putative hydrolase
756	*sav_4899*		GCCGATCGCC	putative hydrolase
757	*sav_6707*		ACCGCGGAGCGCACCTTGGC	putative ribosomal pseudouridine synthase
**Membrane bioenergetics (35)**				
758	*sav_5039*	*cofE*	GCCGACGCACCCTCGTCGGC<br>GCCGCCGCGCGCACCGCGCCCGGC<br>GCCGACGAGGGTGCGTCGGC	putative F420-0:gamma-glutamyl ligase
759	*sav_3794*	*cofG*	GCCGATGACGATCCCCGGC	putative 7, 8-didemethyl-8-hydroxy-5-deazariboflavin synthase subunit 1
760	*sav_6316*	*ctaB*	ACCGAGCGGCCTCGGACGCCTCGGC<br>GCCGCTGGCCCCGGC	putative protoheme IX farnesyltransferase (complex IV)
761	*sav_6047*	*ctaC*	ACCGCGCGCTCGGC<br>ACCGACCAGCGAGGCCGCAGCC	putative cytochrome c oxidase subunit II (complex IV)
762	*sav_6537*	*ctaD2*	ACCGCTTCCGACGGCCGCC	putative cytochrome c oxidase subunit I (complex IV)
763	*sav_3899*	*ctpE*	GCCGCCCGCC	putative integral membrane ATPase
764	*sav_4260*	*cydA1*	ACCGACCCAGGCCCTGCC<br>ACCGCGACCCTCGCC	putative cytochrome bd-I oxidase subunit I (cytochrome

				bd complex)
765	*sav_1923*	*cydA2*	GCCGCTGGCCGCC<br>GCCGAAGACATCGCCCCACCGGC	putative cytochrome bd-I oxidase subunit I (cytochrome bd complex)
766	*sav_6113*	*cynT3*	GCCGCGCACGCGGGGAGATTCCGGC	putative carbonic anhydrase
767	*sav_1941*	*fdh*	GCCGCCGCTCCGGC	putative NAD-dependent formate dehydrogenase
768	*sav_582*	*fdxA*	ACCGAAGAGATCAGCCGCACCGGC	putative ferredoxin
769	*sav_3129*	*fdxD*	GCCGCGCTCCCGCGCGCCAGGC<br>GCCGCCTGGC	putative ferredoxin
770	*sav_4276*	*fhbA*	GCCGCATTGGC	putative flavohemoprotein
771	*sav_5953*	*fhbB*	GCCGACCTCGGC<br>GCCGAGGTCGGCCGGC	putative flavohemoprotein
772	*sav_1481*	*fixA*	GCCGCCCCGACCGGGATCGGC	putative electron transfer flavoprotein, beta subunit
773	*sav_1507*	*fprB*	GCCGAAGCAGGGAGTCCCGCC	putative NAD (P)H-ferredoxin reductase
774	*sav_6956*	*fprF*	GCCGCATGACGACGGCAACCGCC	putative NAD (P)H-ferredoxin reductase
775	*sav_7367*	*hydB*	GCCGCACCTGACCCGCC	putative cytochrome C3-like [NiFe] hydrogenase large subunit
776	*sav_7482*	*lldA*	GCCGCACACCCCGGC<br>GCCGCGGCTCGGC<br>GCCGAAATCCGTCCGTCGCC<br>GCCGAGCCGCGGCTGCTCCGCC	putative L-lactate 2-monooxygenase
777	*sav_1892*	*ndh1*	ACCGAGCCGGC<br>ACCGCCGTCGGC<br>GCCGCCCCTGCC<br>ACCGACCCGCC	putative NADH dehydrogenase (complex I)
778	*sav_3229*	*ndh2*	ACCGAATCGTCGCCCAGCAGGC<br>GCCGAGCTGCGTACAGGC	putative NADH dehydrogenase (complex I)
779	*sav_4109*	*ndh3*	GCCGCGCAGGGTTGGCCGAATTGCC	putative NADH dehydrogenase (complex I)
780	*sav_5661*	*nirB*	GCCGCTCCGCTCCCGCCGCCGCC<br>GCCGAGTTCGCC	putative nitrite reductase (NAD (P) H) large subunit
781	*sav_4837*	*nuoA1*	ACCGCTCATCCGGC	putative NADH dehydrogenase I chain A (complex I)
782	*sav_4881*	*nuoA2*	GCCGCGGCTCGCCGCCAGGC	putative NADH dehydrogenase I chain A (complex I)
783	*sav_4661*	*ppa*	ACCGCGACCGGC<br>ACCGCGACTGGCGCGGACCGCC	putative inorganic pyrophosphatase
784	*sav_4069*	*ppk*	GCCGCTCCCGGCCACGCGGCTCGGC<br>ACCGCACGGCTCGGCCCGGC	putative polyphosphate kinase

			GCCGAGCCGTGCGGTCGCC	
785	*sav_4037*	*sseA*	ACCGCCCCGGCCCCCTTGGC<br>GCCGCGTCGCCGCC<br>GCCGCGTCGTACGCCACCAGGC<br>GCCGCACCTGCC<br>GCCGCTTCGTGAGGTCCGCC	putative thiosulfate sulfurtransferase
786	*sav_1336*	*sseC*	GCCGAAACCATAACCCCAGGC<br>ACCGCATGTCTTCGGC	putative thiosulfate sulfurtransferase
787	*sav_1205*	*ssuD1*	GCCGCGCCTGATCAGCCTGGCCGGC	putative alkanesulfonate monooxygenase
788	*sav_7243*	*trxB2*	GCCGAACAAGCACGCGCGTAGGC<br>GCCGACGTGCCCGCAGGTCAGGC	putative thioredoxin reductase
789	*sav_561*		ACCGCCCCGGC	putative NADH dehydrogenase/NAD(P)H nitroreductase
790	*sav_589*		ACCGAATCTTCCCGGC	putative F420-dependent dehydrogenase
791	*sav_1208*		ACCGCCCGTCCGCC<br>ACCGCGGCTCTTTTTCCGGC<br>GCCGAATCCCTGACGCTGCC<br>GCCGACCCGCC<br>GCCGAGCGCATTCCAGCCGTAGGC<br>GCCGCATGTCAGCC	putative monooxygenase
792	*sav_3213*		GCCGCCCGCTCGCCTGTCGCC<br>ACCGCTGGCCCGGCTCACCCGCC<br>GCCGCACCTGGGCGACAGGC	putative NADH dehydrogenase/NAD(P)H nitroreductase
**Metabolism of cofactors and vitamins (66)**				
793	*sav_1833*	*apbA*	GCCGAGCCTTGGAGGCCGGTCCGGC	putative 2-dehydropantoate 2-reductase
794	*sav_6088*	*bfrA*	GCCGCATCCTTGCACCGGC	putative bacterioferritin
795	*sav_5421*	*bioY*	GCCGATCACCGTCAGCC	putative biotin synthase
796	*sav_3330*	*birA*	GCCGAAAGGTATCGCCGCC	putative biotin apo-protein ligase
797	*sav_4961*	*coaA*	ACCGCGGTCCATACCTGGC	putative pantothenate kinase
798	*sav_6417*	*cobD*	GCCGCCGTACTTGGCTCCGGC<br>ACCGCGCCGGACTCACACCGGC	putative cobalamin biosynthesis protein
799	*sav_6407*	*cobJ*	GCCGCCGTCGCGGAAATCAGGC	putative precorrin methylase
800	*sav_6794*	*cobL2*	GCCGCCGGAACCCGGCCCGGC	putative precorrin-6Y C5,15-methyltransferase
801	*sav_6026*	*cobS*	GCCGCGCTCACCCCCGGGGCCGCC	putative cobalamin (5'-phosphate) synthase
802	*sav_6795*	*cobT2*	ACCGCACTCCGGTCACCCGGC<br>ACCGACCGCC	putative nicotinate-nucleotide-dimethyl

				benzimidazole phosphoribosyltransferase
803	*sav_6032*	*cobU*	GCCGAAGCCGGATCTTGATCGGC<br>GCCGATCAAGATCCGGCTTCGGC	putative adenosylcobinamide kinase/adenosylcobinamide phosphate guanylyltransferase
804	*sav_2424*	*cobW*	GCCGAGCGGCTCTGATCGGC<br>GCCGCCCACCGCTCGCC<br>GCCGATCAGAGCCGCTCGGC<br>GCCGAATGGGGCCGGC	putative cobalamin synthesis protein
805	*sav_109*	*cyp1*	ACCGCGACCCGCC<br>GCCGACCGGCCAGCCGGC<br>GCCGCCTGCAACAACGGCCCCGCC<br>ACCGCTGCCCGGC<br>GCCGAGACGGACCTCGACGGCCGGC<br>ACCGCTCGCTGGTCGCCCTGCC	cytochrome P450 hydroxylase
806	*sav_575*	*cyp2*	GCCGCGGCCGCC<br>GCCGCCGGCTGGC<br>ACCGAATGGCGCCCTGCCCGCC<br>GCCGCCAGCCGGC<br>GCCGAACGCTCTCCCGGC	putative cytochrome P450/NADPH-ferrihemoprotein reductase
807	*sav_1171*	*cyp5*	GCCGCGCGGTCCCTTCGGC<br>GCCGATACCGGGATCGCC<br>GCCGCGATGCCCGAGCAGCC<br>ACCGCCCGCGCACCCGACGGCCGGC<br>ACCGCGGATCCCGCC	putative cytochrome P450
808	*sav_1611*	*cyp7*	GCCGCACTCGCC	putative cytochrome P450
809	*sav_2061*	*cyp9*	GCCGCGCCCTGCC	cytochrome P450 hydroxylase
810	*sav_3536*	*cyp16*	GCCGCTCGCC	putative cytochrome P450
811	*sav_3704*	*cyp17*	GCCGCCCCGGCCAGCC	cytochrome P450 hydroxylase
812	*sav_4539*	*cyp20*	ACCGAGCAGACACCCCTGGTCAGGC	putative cytochrome P450
813	*sav_5111*	*cyp21*	GCCGCGCGCCGCC	putative cytochrome P450
814	*sav_5841*	*cyp22*	GCCGCAAGGGTCTTGCAGCC<br>GCCGCGCCCCTTGCC	cytochrome P450 hydroxylase
815	*sav_6249*	*cyp23*	GCCGACCGCC	cytochrome P450 hydroxylase
816	*sav_6796*	*cysG*	ACCGCCCCGGC	putative uroporphyrin-III methyltransferase
817	*sav_6873*	*dfp*	ACCGCGCCCCCGGCACACCACAGGC	phosphopantothenoylcysteine decarboxylase
818	*sav_4670*	*folB*	ACCGCACTCGGTGGATGCCGGC<br>GCCGCGCCCGCC<br>ACCGAGTGCGGTCGGCGCACCAGCC	putative dihydroneopterin aldolase
819	*sav_5452*	*folC*	GCCGAGGTGGTGGGGCCCGGC<br>ACCGATCGCGACCCCGATCTCGCC<br>GCCGCACGGCTGCCCGAGACTGCC	putative folylpolyglutamate synthase

			GCCGCACGCGCCGGC<br>ACCGACACAGGC	
820	*sav_3442*	*folD1*	GCCGCGCTGCC	methylenetetrahydrofolate dehydrogenase and methenyltetrahydrofolate cyclohydrolase
821	*sav_543*	*folD2*	ACCGCTGACTGGC	methylenetetrahydrofolate dehydrogenase and methenyltetrahydrofolate cyclohydrolase
822	*sav_4667*	*folE*	GCCGCCCTCATCAGGC<br>GCCGCGCCCCCCAGGTTCTAGCC<br>GCCGCGCCCCCCAGGC	putative GTP cyclohydrolase I
823	*sav_3123*	*folP1*	GCCGACGCGCAGCGCGTCGGC<br>GCCGACGCGCTGCGCGTCGGC<br>GCCGACCCGATCCGAAAGGCAGGC	putative dihydropteroate synthase
824	*sav_4672*	*folP2*	GCCGCCCGCCGGACGGGCAGGC<br>GCCGCGAGGCGGCCGGC<br>GCCGACCACCGGGAGGCCTAGGC<br>ACCGCCCTGCC<br>ACCGCCCTGCC<br>GCCGCCGGGCGGGTGGGGGCTGCC<br>GCCGAATCGCC	putative dihydropteroate synthase
825	*sav_4864*	*grcC*	ACCGCCCGGC	putative polyprenyl diphosphate synthase
826	*sav_4742*	*hemB*	GCCGCGGGAGCACCTTTGCC	putative 5-aminolevulinc acid dehydratase
827	*sav_1038*	*hemC2*	GCCGCGGAGGACGGGAGTCGCC<br>GCCGCGTCCGAAGGGTGCGGCTGCC<br>GCCGATCGGC<br>GCCGATCGGC	putative porphobilinogen deaminase
828	*sav_2228*	*hemE*	ACCGACAGCCCCGCC	putative uroporphyrinogen decarboxylase
829	*sav_2407*	*hemH*	GCCGACAACGATCTCGTCACCCGCC	putative ferrochelatase
830	*sav_1663*	*idi*	GCCGCGCACCGGGAATCGCC	putative isopentenyl-diphosphate delta-isomerase
831	*sav_3969*	*ispD*	GCCGCCCGCTCAGCC<br>GCCGCAGCGCCCGGC	putative 2-C-methyl-D-erythritol 4-phosphate cytidylyltransferase
832	*sav_6010*	*lipB*	GCCGCGTGCCCGGATCGCC<br>GCCGATGTCCGTCAGCC	putative lipoate-protein ligase
833	*sav_4831*	*menG*	ACCGACCGGC	putative

				ubiquinone/menaquinone methyltransferase
834	*sav_6100*	*metF*	ACCGCCGGGCCCTGGC	putative 5, 10-methylenetetrahydrofolate reductase
835	*sav_3672*	*moaA1*	GCCGCGACCCGTGCCACCGGC	putative molybdopterin biosynthesis protein
836	*sav_3059*	*moaE*	GCCGCCCGTCACCCCGCGACAGCC<br>ACCGACGTCGGC	putative molybdoprotein biosynthesis protein
837	*sav_6041*	*nadA*	GCCGACGGTTGCAACCGGC	putative quinolinate synthetase
838	*sav_5478*	*nadD*	GCCGCCCGCAGGCGCCCGGC<br>GCCGCCCCCGGGGCCCGATACCGGC	putative nicotinate-nucleotide adenylyltransferase
839	*sav_5948*	*nadE*	GCCGACAGCGCGTCGCGCCCGCC<br>GCCGCACGGTGTCGGC<br>GCCGCGCCGACTGTACGGACTGCC<br>GCCGCGGGGGCGCCGCC<br>GCCGCTCGCCCGGGCACCGGC<br>GCCGCCCTCAGCCCCTCGGC	putative NH(3)-dependent NAD (+) synthetase
840	*sav_7245*	*panD*	GCCGCGAGATCCGCC<br>GCCGCCACCCACATCGGC<br>GCCGCGTCTGCGAATGCCTCGGC<br>GCCGCACCACCTCTCGGC<br>GCCGAGTTCCCCCCAGCCGCC	putative L-aspartate 1-decarboxylase
841	*sav_3834*	*pdxH2*	GCCGAACAGGTCGAGCAGCC<br>GCCGAGCGCGTGAACCGGC	putative pyridoxamine 5'-phosphate oxidase
842	*sav_6830*	*pdxS*	ACCGCCTCCTCATTGGC	putative pyridoxine biosynthesis protein (lyase)
843	*sav_6993*	*phzC*	ACCGCCTGTCAGCC<br>ACCGCCCTGGC<br>ACCGCCCGCC<br>GCCGCGTGCCCCCGCGTCTCGGC<br>GCCGCCGAGCCCCAGGAGCCGCC	putative phenazine biosynthesis protein
844	*sav_6084*	*phzE*	ACCGCCTGTGTAGGC<br>GCCGCACCGGACGACCGGC<br>GCCGAATCGGGTAAGGTGCCTGGC<br>ACCGCTCATCCGGC<br>GCCGCCCGCC	putative anthranilate/para-aminobenzoate synthases component
845	*sav_5157*	*pncA*	GCCGCCCGCGCACCGGC<br>GCCGAATGCCCCCTCCCGCC<br>GCCGCTTCCGAGGCCCCCGCCCGCC	putative nicotinamidase
846	*sav_404*	*pntA*	GCCGCGCCCGATGAACCGCCCGCC<br>ACCGATCATTATCGGC	putative pyridine nucleotide transhydrogenase, alpha subunit
847	*sav_545*	*ribA4*	ACCGAGCGAGATGGGCCGCCCCGGC	putative GTP cyclohydrolase II

848	*sav_6902*	*ribC*	GCCGCCGATCGGC<br>GCCGATCGGC<br>ACCGATCGCC<br>GCCGCGCACTGCC	putative riboflavin synthase alpha subunit
849	*sav_2546*	*ribF*	GCCGCCCGTCACAGCC<br>GCCGCGTCCGTTCGGC	putative riboflavin kinase
850	*sav_5037*	*rmlA*	GCCGAACGCGGTTCCGGCCGGC<br>GCCGCTCCGACGGCCGCC<br>ACCGCGTTCGGCCGGC	putative nucleotide phosphorylase
851	*sav_6099*	*thiE*	GCCGCGGTCCGTACACCGCC<br>ACCGACAGGCGGTCCGTAGGC<br>ACCGCGGCGGGGCATGGCCGGC	putative thiamine-phosphate pyrophosphorylase
852	*sav_3945*	*thiX2*	GCCGAAACGCTAGTCTCCGGC	putative flavin-dependent reductase
853	*sav_4811*	*ubiD*	GCCGCCCCACGGCGAACCGGC	putative 3-octaprenyl-4-hydroxybenzoate carboxy-lyase
854	*sav_4809*	*ubiX*	GCCGCCCCGCC	putative decarboxylase
855	*sav_1207*		ACCGCTCGGGGCGCCTGCC	putative NAD(P)H-dependent FMN reductase
856	*sav_2756*		GCCGCGGCCGAATCCCCGGCCGCC<br>GCCGCGGTCGACGGCGACGGCCGGC<br>GCCGATGACGGCGTGGGTTCCGGC	putative pyridoxal-phosphate-dependent aminotransferase
857	*sav_3828*		GCCGCGCCGTCGCTCCGCC<br>ACCGACTCCCGCC<br>GCCGACGGGGACTGCCGGCCTGGC	putative pimeloyl-CoA synthetase
858	*sav_3891*		ACCGCCGCCGCC<br>ACCGCTGTGCCGTTCCCCGCCTGCC<br>ACCGCCCCCGCCCGCC<br>ACCGCCGCCGCC	putative quinone oxidoreductase
**Metabolism of phosphates (3)**				
859	*sav_5583*	*phoH*	GCCGACACCGGC<br>ACCGCAGGCCTCAAGAGCCGGC	putative phosphate starvation-induced protein
860	*sav_5267*	*ppe2*	GCCGATTCGCAAGAATCCTGCC	putative phosphoesterase
861	*sav_2747*		GCCGCGTCGCGTGGGCACTGCCGGC	putative phosphodiesterase
**Protein synthesis, folding and modification (185)**				
862	*sav_6576*	*aprA*	GCCGCGATAACCGCC<br>ACCGCACTGCCCGCCCAGGC<br>GCCGAGAAGTCTTGGC	putative alkaline serine protease (secreted peptidase A)
863	*sav_4748*	*argS2*	ACCGCGTGGGATACTGGC	putative arginyl-tRNA synthetase
864	*sav_4395*	*aspS*	ACCGAAGCGGACAGGC	putative aspartyl-tRNA synthetase
865	*sav_4514*	*clpB1*	GCCGAACCTAGCC	putative ATP-dependent Clp

				protease
866	*sav_2600*	*clpC1*	GCCGCACCCCGGTCCCGCCTCCGCC	putative ATP-dependent Clp protease
867	*sav_4697*	*clpC2*	GCCGACACCGTCCTTGGC	putative ATP-dependent Clp protease
868	*sav_5447*	*clpP1*	ACCGAGGCCCGGC	putative ATP-dependent Clp protease proteolytic subunit 1
869	*sav_7101*	*clpP3*	GCCGCCGCCCCGCC	putative ATP-dependent Clp protease proteolytic subunit 2
870	*sav_1146*	*clpP4*	ACCGATCCTCGCC<br>GCCGCCGGATCCGCTTTCTCTGCC	putative ATP-dependent Clp protease proteolytic subunit 1
871	*sav_4637*	*clpX1*	GCCGCTCCTTGGC<br>GCCGCTGGACCTAGGC	putative ClpX protein
872	*sav_5449*	*clpX2*	ACCGCAGGTCGACACTTGCC	putative ATP-dependent Clp Protease ATP binding subunit
873	*sav_3967*	*cysS*	GCCGCCTGGGGGGCGGCCGGC<br>GCCGCCCCCCAGGCGGCAGCC	putative cysteinyl-tRNA synthetase
874	*sav_7237*	*dnaK2*	ACCGCTCGCCTGTAGGC	putative heat shock protein Hsp70
875	*sav_1111*	*dppA1*	ACCGCACGCCGGGCGCCCGGC	putative D-aminopeptidase (family M55 peptidases)
876	*sav_1900*	*dppA2*	GCCGCCTCCACACCCGGC	putative D-aminopeptidase
877	*sav_6658*	*eif2*	GCCGCGACAGAGGTCGCCGGC<br>GCCGACGTTCGCC	putative initiation factor eIF-28 alpha subunit
878	*sav_2086*	*engC*	GCCGCTGGTGACTTGGC<br>GCCGCGGGCAGGC<br>ACCGCGCTCACTGGC	putative ribosome-associated GTPase
879	*sav_2624*	*frr*	ACCGACAAGGACCGCTGCC	ribosome recycling factor
880	*sav_6825*	*fusA2*	ACCGCGGGCGTCATCGGAGCCGCC	translation elongation factor G
881	*sav_1799*	*fusA3*	ACCGCGCGCCGCC<br>GCCGCCCGTCGGC<br>GCCGAGCAGGCCCGTGTCGCC<br>GCCGCGACGTGTTGTCGGC<br>GCCGCGCAGCCGGC<br>GCCGACGGGCGGCCGGC	translation elongation factor G
882	*sav_2746*	*gatC*	GCCGCCCGTCCGCC<br>ACCGATCCGGC	putative aspartyl/glutamyl-tRNA(Asn/Gln) amidotransferase subunit C
883	*sav_3931*	*groEL2*	GCCGCTTGCACTCGGC<br>GCCGAGTGCAAGCGGCTCCAGGC<br>ACCGCGCTGGC	putative class I heat-shock protein
884	*sav_4991*	*groES1*	GCCGAGACCACGTCGTACGCCGCC<br>GCCGCCGCAATTGGC<br>GCCGCACTTTTCCGACGACCGGC	putative GroES

			GCCGCACCCACCCGACGACCGGC<br>GCCGATCCGTCCGGTCTCCGGC	
885	*sav_7373*	*hypA*	GCCGCGCTCGTCAGGCTGGC<br>GCCGCCGGGGTACGCCGTTCGCC	putative [NiFe] hydrogenase expression/formation protein
886	*sav_6130*	*ileS*	GCCGAGGCCCAGCAGCC<br>GCCGCGTCCGCC	putative isoleucyl-tRNA synthetase
887	*sav_4949*	*infA1*	ACCGAGAGCGCCCCTAGGC	putative translation initiation factor IF-1
888	*sav_504*	*infA2*	ACCGCTCCGGGTTCGCC<br>ACCGCGACCAGCC	putative translation initiation factor IF-1
889	*sav_6737*	*infC*	ACCGCCTGACCGGGTGACCCGCC	putative translation initiation factor IF-3
890	*sav_5488*	*leuS*	GCCGCGCTGATCAGCC	putative leucyl-tRNA synthetase
891	*sav_4749*	*lysS1*	ACCGCGTGGGATACTGGC	putative lysyl-tRNA synthetase
892	*sav_7296*	*lysS2*	GCCGCTTCCGATAGCCTCACAGCC<br>GCCGCGACCCCGCC	putative lysyl-tRNA synthetase
893	*sav_4948*	*map3*	GCCGCGGCCGTACTGTTGTGTAGGC<br>GCCGCGCACCTCGCCGCCGGC	putative: methionyl aminopeptidase, secreted
894	*sav_4403*	*metS*	GCCGAAACGCCGCC<br>GCCGACATGGGTACGCCGCC	putative methionyl-tRNA synthetase
895	*sav_6114*	*mraW*	GCCGCGCACGCGGGGAGATTCCGGC<br>ACCGCCGGGTCCCCGCC	S-adenosylmethionine-dependent methyltransferase
896	*sav_2513*	*osaC*	GCCGCGTCCTAGTACTCGGC<br>GCCGAGTACTAGGACGCGGCAGGC<br>GCCGCGCGTCTGCC<br>ACCGCCCAGGCCGGACGGACCGGC	putative osmotic regulatory protein (prpM2, magnesium/manganese-dependent protein phosphatase)
897	*sav_6025*	*pepA*	GCCGCCGCCACCCGCC<br>ACCGCCGCTCGGC<br>GCCGAACGGATGGGGTCGACCGGC	putative leucyl aminopeptidase (cytosol aminopeptidase)
898	*sav_3115*	*pepD1*	GCCGAAGTGGTGGAGCCGCC<br>GCCGACCCGTTCGGGGGCCCGCC<br>GCCGATGCCGGCGCCGGC<br>GCCGAGTCGGC<br>ACCGCGTCCGGCTCCGGC<br>ACCGCCCCACCGGCGCCGGC<br>GCCGACTCGGC<br>GCCGACTGAGCTCGAAGTCGCC	putative serine protease
899	*sav_4223*	*pepD3*	GCCGCGGACCCACAGCC<br>ACCGAGAACAACAGCC	putative serine protease, secreted
900	*sav_3769*	*pepN1*	ACCGCACCGAAGTCGGC	putative aminopeptidase N
901	*sav_5395*	*pepN2*	ACCGATCCTGCC	putative aminopeptidase N
902	*sav_6212*	*pepN3*	GCCGCCCCTGTGCCCTGTCGGC	putative aminopeptidase N
903	*sav_7064*	*phoD2*	GCCGACGCACACCGCC	putative alkaline phosphatase,

			GCCGACAAGCGCTTCGCGGCCGGC	secreted
			GCCGCGAGGCGCTGGTCGGC	
			GCCGCGAAGCGCTTGTCGGC	
			ACCGACCCCGGC	
904	*sav_875*	*pip*	GCCGATGCAGGCAGGC	putative proline iminopeptidase
			GCCGAATCGAGCGAATCGGC	
			GCCGCTCGGATCGGC	
			GCCGCCGTCCAGCC	
			GCCGATTCGCTCGATTCGGC	
905	*sav_639*	*pkn1*	GCCGCCCGCC	putative serine/threonine
			GCCGCCGCCCCGATGCCGGC	protein kinase
906	*sav_975*	*pkn2*	GCCGCGCAGGACCGGC	putative serine/threonine
			ACCGAACTGCC	protein kinase
907	*sav_2262*	*pkn5*	ACCGCCCCGCAATCGCCACAGCC	putative serine/threonine
				protein kinase
908	*sav_3540*	*pkn6*	GCCGCACCGTCGCCGGC	putative serine/threonine
			GCCGCCCTGCC	protein kinase
909	*sav_3816*	*pkn7*	ACCGCTCCGCGGGCCGCC	putative serine/threonine
			ACCGCACCGCC	protein kinase
910	*sav_4326*	*pkn10*	ACCGCCCGGC	putative serine/threonine
			GCCGCACACGGCATCCGGC	protein kinase
			GCCGAGGGCCGAGGTACGGCCGCC	
			ACCGACTGCC	
			GCCGACGCGCAAAAGGTGTCCGGC	
911	*sav_4371*	*pkn12*	ACCGCCGCCAGTCGCC	putative serine/threonine
			ACCGCCCGTACAGCC	protein kinase
912	*sav_4372*	*pkn13*	ACCGCCCGTACAGCC	putative serine/threonine
			ACCGCCGCCAGTCGCC	protein kinase
913	*sav_4554*	*pkn14*	ACCGCTCCGCCCACCCGCC	putative serine/threonine
			GCCGCGCCGCC	protein kinase
914	*sav_4619*	*pkn15*	GCCGACGCGCCCCGGC	putative serine/threonine
				protein kinase
915	*sav_4717*	*pkn16*	GCCGAGGCTAGCAAGCCGCC	putative serine/threonine
				protein kinase
916	*sav_5005*	*pkn18*	GCCGCTACACGGAGGGACCGCC	putative serine/threonine
			ACCGCCCGCCGCC	protein kinase
917	*sav_5006*	*pkn19*	ACCGAGGTGACGTTCCGCC	putative serine/threonine
				protein kinase
918	*sav_5007*	*pkn20*	GCCGATCAGCCGGATCAGCC	putative serine/threonine
			GCCGAATTCAGCC	protein kinase
919	*sav_5008*	*pkn21*	GCCGCCGGACATACGGGCTCGGC	putative serine/threonine
			GCCGAGCCCGTATGTCCGGC	protein kinase
920	*sav_5100*	*pkn23*	ACCGCACACGAGCACGTCGGC	putative serine/threonine
			GCCGACGACAGCC	protein kinase

			GCCGCAGAACCTGGC	
921	*sav_6799*	*pkn30*	ACCGCCGTCTCCACCCGGC	putative serine/threonine protein kinase
922	*sav_7509*	*pkn32*	GCCGATGTGTTCGCC	putative serine/threonine protein kinase
923	*sav_6842*	*ppiB*	GCCGACCGCC	putative peptidyl-prolyl cis-trans isomerase, secreted
924	*sav_2812*	*prcB2*	ACCGCCACCGCCACCGCCGCC<br>ACCGCCACCGGC	putative 20S proteasome beta-subunit
925	*sav_5102*	*prfB*	GCCGACGCACCGCC	putative peptide chain release factor 2
926	*sav_2558*	*proS1*	GCCGCCGTCGCC	putative prolyl-tRNA synthetase
927	*sav_2646*	*proS2*	GCCGAGAGTTGCC	putative prolyl-tRNA synthetase (eukaryote type)
928	*sav_4263*	*prpA2*	ACCGCGGGCCGCTGCGACCAGGC<br>ACCGCGGCTTTCGCC<br>ACCGCATCGGACTGCC	putative serine/threonine protein phosphatase
929	*sav_321*	*prpB1*	GCCGCGGTTACCAGCC<br>GCCGAGCCCGGTACCGGACAGGC<br>ACCGCCCGCCGTCCGGC	putative magnesium/magnanese-dependent protein phosphatase
930	*sav_956*	*prpB2*	GCCGATCACGACTGGC	putative magnesium/magnanese-dependent protein phosphatase
931	*sav_1334*	*prpB6*	ACCGCCTCCTCGGCCGCC<br>GCCGATGTCGGGCACGTACCGCC<br>ACCGCCTCGGC	putative magnesium/magnanese-dependent protein phosphatase
932	*sav_4229*	*prpB7*	GCCGCCTCCGCC	putative magnesium/magnanese-dependent protein phosphatase
933	*sav_4415*	*prpB9*	GCCGCATCCCGACTTGCCAGCC	putative magnesium/magnanese-dependent protein phosphatase
934	*sav_6744*	*prpB12*	GCCGACGCCCAGCAGTTCCCCGGC<br>GCCGCATGGGCGCGGCCGGC<br>GCCGATGGCCCCCCATCAGGC	putative magnesium/magnanese-dependent protein phosphatase
935	*sav_698*	*prpC1*	GCCGCGGGTTCGCC<br>ACCGCCCAGCGCCCTGCC	putative magnesium/magnanese-dependent protein phosphatase
936	*sav_924*	*prpC4*	GCCGCGCCTGGC<br>GCCGAAAGGACCTTCGCCCGGC<br>ACCGACTCCGGC<br>ACCGAAGAGTCGGC<br>GCCGCCCGCC	putative magnesium/magnanese-dependent protein phosphatase

937	*sav_1235*	*prpC5*	GCCGAGGTGTGCCGCC	putative magnesium/magnanese-dependent protein phosphatase
938	*sav_4151*	*prpC6*	GCCGCCGAAGTCGGTTCGGC<br>GCCGCCCGCC<br>GCCGAACCGACTTCGGC	putative magnesium/magnanese-dependent protein phosphatase
939	*sav_7504*	*prpC7*	ACCGAGCCTTCCAGCC<br>ACCGCGGGACCGGCGGACCGGC	putative magnesium/magnanese-dependent protein phosphatase
940	*sav_350*	*prpD*	GCCGCATACAGGC<br>GCCGACCGGCGGGCCGGGGCCGGC<br>GCCGCCTGCC<br>GCCGCCAGCCACCGCCGCCTGGC<br>GCCGAACGAACTAGCCGACCGGC<br>GCCGATCTGCGCCCAGCCGGC	putative magnesium/magnanese-dependent protein phosphatase
941	*sav_3223*	*prpE2*	GCCGATCCGGTGTAGCC	putative magnesium/magnanese-dependent protein phosphatase
942	*sav_4394*	*prpE3*	ACCGAAGCGGACAGGC	putative magnesium/magnanese-dependent protein phosphatase
943	*sav_5727*	*prpH*	GCCGCCGCAGGC<br>GCCGCGCAACCGGC<br>GCCGCTTGTTAGCC	putative magnesium/manganese-dependent protein phosphatase
944	*sav_4108*	*prpI3*	GCCGACGTCTGACGGATGTCGGC<br>GCCGACATCCGTCAGACGTCGGC	putative magnesium/magnanese-dependent protein phosphatase
945	*sav_587*	*prpJ1*	GCCGACCGGGCGGCCGACAGCCGCC<br>GCCGCCCGGTCGGC	putative magnesium/magnanese-dependent protein phosphatase
946	*sav_767*	*prpJ2*	ACCGACAACCACGACCAGCC	putative magnesium/magnanese-dependent protein phosphatase
947	*sav_786*	*prpJ3*	GCCGAGTCTGCCCCGCC<br>GCCGCGCTCAGATCACCCCGCC<br>ACCGACTGCACCGGC	putative magnesium/magnanese-dependent protein phosphatase
948	*sav_1054*	*prpJ4*	GCCGCACGGGTCGCGGATCGCC<br>GCCGCCAACCCCCGGC<br>GCCGCTCCTTCCGGC	putative magnesium/magnanese-dependent protein phosphatase
949	*sav_1391*	*prpJ5*	GCCGATCCGGCACGTCGGC<br>ACCGCGCCCGGCGGGCTGCCGGC<br>GCCGACGTGCCGGATCGGC	putative magnesium/magnanese-dependent protein phosphatase
950	*sav_7172*	*prpJ7*	GCCGCCGCGGCCCCCTGGCACCGCC<br>GCCGAACGAAGGCACGGCAGCC	putative magnesium/magnanese-depend

			GCCGCCCCCCGTCGCATGCTGCC<br>GCCGCGGCGGCGGGCTCCGGC<br>GCCGCCTGCCGCCGGCCGCCGGC<br>GCCGCTCGACGAGGTGCCGGC	ent protein phosphatase
951	*sav_7498*	*prpJ8*	GCCGCCCGCC<br>ACCGAGAACCCGGC<br>GCCGCGGGAATCGGCACCGGC	putative magnesium/magnanese-dependent protein phosphatase
952	*sav_5565*	*prpK*	ACCGAAGTGGGCCGCC	putative magnesium/magnanese-dependent protein phosphatase
953	*sav_1908*	*prpL2*	ACCGACCGGC	putative magnesium/magnanese-dependent protein phosphatase
954	*sav_7478*	*prpL4*	GCCGCGGTGGCGATCCGGC<br>GCCGCGGGCCGGC	putative magnesium/magnanese-dependent protein phosphatase
955	*sav_437*	*prpM1*	GCCGCAGATCCGGAGCCTGGC<br>ACCGCCCAGGC<br>GCCGCCCGGC<br>GCCGCACCATAGTCACCATCGCC	putative magnesium/magnanese-dependent protein phosphatase
956	*sav_3185*	*prpM3*	GCCGCCCCTCGGCATCCGCC	putative magnesium/magnanese-dependent protein phosphatase
957	*sav_7288*	*prpM4*	GCCGCAGTCGGC<br>GCCGCCGGGCCGGCCGCCCGGC<br>GCCGCGGTCGCGGTGGAGGCCGCC<br>ACCGCCGGGCGGCCGGCCCGGC	putative magnesium/magnanese-dependent protein phosphatase
958	*sav_7330*	*prpM5*	ACCGCACCCGAAGAACGCCCGGC	putative magnesium/magnanese-dependent protein phosphatase
959	*sav_994*	*ptpA1*	GCCGACCCGGC	putative conventional protein tyrosine phosphatase
960	*sav_2948*	*ptpA3*	GCCGAGCCGGGTGCCGACCTGCC<br>GCCGCTCGCC<br>GCCGACCGGC<br>GCCGAGATCGTTGGCAGGTCGGC	putative conventional protein tyrosine phosphatase
961	*sav_3669*	*rimJ*	GCCGACCTACCGCC	putative ribosomal-protein-alanine N-acetyltransferase
962	*sav_4911*	*rplA*	GCCGAGCAGGCCCTGCC	putative ribosomal protein L1
963	*sav_4941*	*rplF*	ACCGAGTCCTGCC<br>ACCGAGGTCCGCC	putative ribosomal protein L6
964	*sav_4912*	*rplJ*	GCCGACCGAGCGGATTTGCC<br>ACCGCTGGTCGTTGCC	putative ribosomal protein L10

			GCCGCGCGCTGTCCCCGCGCCGGC	
965	*sav_4910*	*rplK*	ACCGAGCAGGTCAGACCGGC	putative ribosomal protein L11
966	*sav_4913*	*rplL*	GCCGAACGTACGCCCGCC<br>GCCGAATAGTGGAAGGATCGCC	putative ribosomal protein L7/L12
967	*sav_4957*	*rplM*	ACCGAGGTCCGCC<br>GCCGCTGGTCATCGGC	putative ribosomal protein L13
968	*sav_2640*	*rplS*	GCCGCTGCTGTACGTCGTCCGGC	putative ribosomal protein L19
969	*sav_5467*	*rplU*	GCCGCCCCGTAACCTTGGC<br>GCCGCCCGTCCCGGC<br>ACCGCAAGCGGCCTCTCCCTGGC	putative ribosomal protein L21
970	*sav_3563*	*rplY*	ACCGAACAGTGCGGCCTCCCTCGCC	putative ribosomal protein L25
971	*sav_2675*	*rpmB*	GCCGCAAGAGCGCCGCGAGACCGCC<br>GCCGCTGGCAAGGGCGCGGGCCGGC<br>GCCGCCGGGGTGGTCCGGC<br>ACCGCGGCCGGCCCGCGCCCTTGCC	putative ribosomal protein L28
972	*sav_1572*	*rpmE1*	GCCGCCTCCGGGCTTTCCCCGCC	putative ribosomal protein L31
973	*sav_2913*	*rpmE2*	GCCGACGTACCAGTCTGCC	putative ribosomal protein L31
974	*sav_4900*	*rpmG*	ACCGCTACTGACAGTAGCC<br>GCCGATCGCC<br>ACCGACCCCACAATCGGC	putative ribosomal protein L33
975	*sav_4315*	*rpmH*	GCCGCCTGCC	putative ribosomal protein L34
976	*sav_6233*	*rpsA*	ACCGCATACCCGGC	putative ribosomal protein S1
977	*sav_2627*	*rpsB*	GCCGCTGTCCACAACCGGC<br>ACCGAGAGGCGCTGACTTCGGC	putative ribosomal protein S2
978	*sav_4940*	*rpsH*	ACCGCACCCGTCCCGCC	putative ribosomal protein S8
979	*sav_4925*	*rpsJ*	GCCGATGCTGTGCGCCTCCCGCC<br>GCCGACCAGGC<br>ACCGAGCAACTCCGACAGTCTGCC	putative ribosomal protein S10
980	*sav_4917*	*rpsL*	ACCGCGTGGGTCGGC	putative ribosomal protein S12
981	*sav_2524*	*rpsO*	GCCGAGCGCCTCCGTACAGCC<br>GCCGCAAGGATCGCC<br>GCCGCGCCCAGCGGATCCCCGCC	putative ribosomal protein S15
982	*sav_2644*	*rpsP*	GCCGCTGGGTACGGCTGCC	putative ribosomal protein S16
983	*sav_4244*	*serS1*	ACCGAGAATTCTTTGCCCGGC	putative seryl-tRNA synthetase
984	*sav_1000*	*sprC*	GCCGACAGGTCCGGGCCCTGCCGGC<br>GCCGATACGAGGAACAAGCAGCC<br>GCCGACACAAGGAGGCCGCCCGGC	putative streptogrisin C (secreted serine protease)
985	*sav_6553*	*sprD1*	GCCGCGCCGATTGCC<br>GCCGATTGCCGCGCCCATTGCC	putative streptogrisin D (secreted serine protease)
986	*sav_7497*	*sprD2*	GCCGCCGTCCGGC	putative streptogrisin D (secreted serine protease)
987	*sav_7486*	*sti2*	GCCGAACCGTGGAGATCCGGC<br>GCCGAACCGGCACCGGACCGGC	putative subtilisin inhibitor, secreted
988	*sav_3417*	*trpS1*	GCCGAGCGCCCCGCAGGC	putative tryptophanyl-tRNA

				synthetase
989	*sav_4725*	*trpS2*	ACCGACCTGGGTCGGC<br>GCCGCCGCCTCGGC<br>GCCGCCGTCAGGC<br>ACCGCCACCGCCGGCCGGC	putative tryptophanyl-tRNA synthetase
990	*sav_4920*	*tufA1*	ACCGACCGCCGGGGCTCACCCGGC<br>GCCGCCCGCC	putative elongation factor EF-Tu
991	*sav_7028*	*tufA2*	GCCGCGCGTACCCCTTACGTCGGC<br>GCCGCCTTTCCGGGCTGCC<br>GCCGAAGGCTGCC<br>GCCGCGATGATCGGGACGGCAGCC	putative elongation factor EF-Tu
992	*sav_1037*	*zmp1*	GCCGCCCGGCCCCGGCCCGGC<br>GCCGCCCGGCCTCGCC	putative griselysin (secreted neutral zinc metalloprotease)
993	*sav_1128*	*zmp2*	ACCGAAGTCGCAGCGCCTCCGGC	putative griselysin (secreted neutral zinc metalloprotease)
994	*sav_2795*	*zmp4*	GCCGAACGACGTGGGGTAGCC	putative griselysin (secreted neutral zinc metalloprotease)
995	*sav_5630*	*zmp5*	GCCGACTCCCCCGCCCGCCGTCGGC<br>GCCGCCCCACGCGGCCGCC<br>GCCGACGGCGGGCGGGGGAGTCGGC<br>ACCGCCGGTAGCGCGATCCGGC	putative griselysin (secreted neutral zinc metalloprotease)
996	*sav_5669*	*zmp6*	GCCGATTTCATCCGCC	putative griselysin (secreted neutral zinc metalloprotease)
997	*sav_734*		ACCGATGCTTCTCCAAAACTGCC<br>GCCGACGCAGCC	putative protease, secreted
998	*sav_897*		GCCGAGCGACCGGC<br>GCCGCTCGGC	putative secreted alpha-amylase inhibitor
999	*sav_1106*		ACCGCGCGGGGCAAGGGCAGGC<br>ACCGCGCCTGCCCTTGCC	putative subfamily M50B peptidases
1000	*sav_1117*		GCCGACAGGC	putative metallopeptidase, secreted
1001	*sav_1180*		GCCGAAATGAGTGTCGCC	putative family M6 peptidase
1002	*sav_1231*		GCCGCCCGGTCCGGC	putative secreted tripeptidyl-peptidase C
1003	*sav_1256*		ACCGATCATTCGGTAGCC<br>ACCGAATGATCGGTCGCC<br>GCCGACCTCTTGCC	putative family S33 peptidase
1004	*sav_1415*		GCCGAGGAGTCGGC<br>GCCGCCGGCTGCTCCACGCCCGCC<br>GCCGCGCGCAGCGGCGCACTGGC<br>GCCGACTCCTCGGC	putative protease
1005	*sav_1471*		GCCGCTTGCCGCACCTGTCTCAGGC<br>GCCGATCCTCCCACAGGGGCCGGC	putative peptidase
1006	*sav_1640*		GCCGAATCCTGCCGCCGCC	putative zoocin A (secreted

			peptidase)
1007	*sav_1674*	GCCGCGGGCATGTCCACAGCC	putative X-Pro dipeptidyl-peptidase, secreted
1008	*sav_1675*	GCCGCTCCTGTCACACGATCGCC	putative metallopeptidase, secreted
1009	*sav_1809*	GCCGCCTCGGC	putative dipeptidase
1010	*sav_1906*	ACCGCGGGTGTCACGGCCCTCGCC	putative secreted peptidase
1011	*sav_2034*	GCCGCGGTCCGGC<br>ACCGCCGCTCGCC<br>ACCGCGCAGTCCCGGC<br>ACCGAGGGTTTTCCCTGCC<br>GCCGCGGGACCGGC<br>GCCGAGGCCGTTTGCC	putative trypsin-like protease, secreted
1012	*sav_2157*	GCCGCGCCGGAGCGAGACCCCGCC<br>GCCGACCGCC	putative carboxypeptidase
1013	*sav_2161*	GCCGACCGGCCCTTTTCGCC<br>GCCGCCCCGACGTGTGTCGGCAGCC<br>ACCGCAATCGGC<br>GCCGAGACCGAACCGGGGCTCCGCC<br>GCCGCCCTGCC	putative peptidase
1014	*sav_2420*	GCCGCGGGCGCGGCGCACTGCC	putative zoocin A (secreted peptidase)
1015	*sav_2422*	ACCGCAGATTTCCTGCC	putative protease
1016	*sav_2443*	ACCGAGCATGTGGAGGCCGTCGGC	putative trypsin-like protease, secreted
1017	*sav_2467*	ACCGATAGCC<br>GCCGATGCCCGATGGTCCTCGCC<br>GCCGAGACCCAAGACCTCGCC<br>GCCGATGGCTGGC<br>GCCGAAGATCCTTGCC	putative 2,4-diaminobutyrate 4-transaminase
1018	*sav_2471*	GCCGCGCGCCGGAGCCGCC<br>GCCGCCCGGC	putative metallopeptidase, secreted
1019	*sav_2628*	ACCGAGAGGCGCTGACTTCGGC<br>GCCGCTGTCCACAACCGGC	putative peptidase, secreted
1020	*sav_2801*	GCCGCGGGCCATCGGGTGCCCGCC<br>GCCGATTCCGCCCGCC<br>ACCGCACGAGAGGCTGCC<br>GCCGCGGTGAATTTCTGCC<br>ACCGCCACAAGGTCGCGCAGGC<br>GCCGACACGCCACTGGGCAGCC	putative secreted peptidase A
1021	*sav_2939*	GCCGACAGGCACGCCCATTGCC<br>ACCGCGCGCTGTGGCGCTCGGC<br>GCCGCAGGATCCACATCCAGGC	putative snapalysin (secreted metalloprotease)
1022	*sav_2957*	GCCGAACCGCC	putative NLP/P60-family

			secreted protein (PgpA peptidase)
1023	*sav_3081*	ACCGCAGCCCGCC	putative tripeptidyl-peptidase C, secreted
1024	*sav_3266*	GCCGCCCCGCAGTCGGCCTTCGCC<br>ACCGATCGAAAACCAGCC<br>ACCGACATAGAGCCGTTGGC	putative carboxypeptidase T (secreted zinc-binding carboxypeptidase)
1025	*sav_3371*	GCCGCACCGTCGCC<br>GCCGCCGGGGCTGCCGGC	putative metal-dependent amidase/aminoacylase/carboxypeptidase
1026	*sav_3462*	ACCGCCGGGTACAGTCGCC	putative zoocin A (family M23 peptidase)
1027	*sav_3464*	GCCGCACGACCCCCACCGGC<br>GCCGCGCGGCCTCCGCC<br>ACCGACCTCGGGGTGAATCGGC<br>GCCGCCTTGAAGGGCTTCAACCGCC<br>GCCGCGCGGCCCGGCGATGTTCGCC	putative NLP/P60-family secreted protein (PgpA peptidase)
1028	*sav_3467*	GCCGCCCGGGCCAGGGGCGGCAGCC<br>ACCGCCGAAAGGTCCGGC<br>ACCGCTGACCGCTCGCGTCCCGGC<br>GCCGCCCCTGGC<br>GCCGCCGCCGGACCTTTCGGC	putative NLP/P60-family secreted protein (PgpA peptidase)
1029	*sav_3952*	GCCGCGGGACCCGCCGGC	putative metallopeptidase, secreted
1030	*sav_4407*	GCCGCCGCACAGGC<br>GCCGACTCGCC	putative snapalysin (secreted metalloprotease)
1031	*sav_4700*	GCCGCCAAGATCAGGGGACTTGGC	putative zoocin A
1032	*sav_5155*	ACCGACTCTATTCGCC<br>GCCGACGCGCCGGTTGGC	putative protease, secreted
1033	*sav_5300*	ACCGACATCCACCTCGGC	putative tripeptidyl aminopeptidase, secreted
1034	*sav_5600*	ACCGCCAGCAGCC	putative metalloprotease
1035	*sav_5709*	GCCGAAGTAGCGGCCTGGC<br>GCCGCGCACCGGCAGAGCCCGGC<br>GCCGCTACTTCGGC	putative protease
1036	*sav_5721*	GCCGAGGATCACACCGTCGGC<br>GCCGCCCTGGCGGAAAGCCGCC<br>GCCGACGGTGTGATCCTCGGC	putative SAM-P45 peptidase, secreted
1037	*sav_6208*	GCCGCCACTGGATTCCACTGTAGGC	putative trypsin-like protease
1038	*sav_6295*	GCCGACACCCACTTGGGTGGTCGGC	putative carboxypeptidase T (secreted zinc-binding carboxypeptidase)
1039	*sav_6452*	ACCGAGTTGAACCGCC	putative tripeptidyl-peptidase S, secreted

1040	*sav_6634*		GCCGCCCAAGGCGGGACCCGGC	putative peptidase
1041	*sav_6649*		ACCGCCGCTTCGGCTGCAACCGCC<br>GCCGAATGCTGCC	putative serine protease
1042	*sav_6673*		GCCGACGCCCCCGCAAACACCGCC<br>ACCGACCCGGC<br>GCCGCACCCCCACCACCCCGGC<br>GCCGACGAAGCCCCCACCCGGC<br>GCCGCCCGCCCCGGC<br>GCCGCTGCCGCTGCC<br>GCCGCCGGGCTCTTTGGGTCGCC	putative peptidase
1043	*sav_6678*		GCCGACTGTTTTTCAGGC<br>ACCGAGGGCGCCGCC	putative proteasome component
1044	*sav_6858*		GCCGACCGGATACCGCC	putative peptidase
1045	*sav_7099*		GCCGAGTCCTGCCGCCGCC<br>GCCGCTGTGGCGGGGGCTTGCC	putative NLP/P60-family secreted protein (PgpA peptidase)
1046	*sav_7250*		GCCGATTGCCGCCTTGTCCTGGC<br>GCCGCTCCATCCCGCC	putative zoocin A (secreted peptidase)
**DNA synthesis, repair, recombination, modification and packaging (83)**				
1047	*sav_7543*	*cas3*	ACCGCAGGTCGCCAGCCGCTGCC<br>ACCGACGTCGTGGGACTGGTTGGC	putative CRISPR-associated helicase Cas3 family protein protein (type I CRISPR)
1048	*sav_6985*	*dinB*	ACCGCCCGGTTGGC	DNA polymerase IV, DNA damage inducible protein
1049	*sav_4285*	*dinF*	GCCGACAGGCCTAACGTGGTCCGGC	DNA-damage-inducible protein F
1050	*sav_2464*	*dinG*	GCCGAATCCGCCCGCCGCC	putative ATP-dependent helicase
1051	*sav_4316*	*dnaA*	GCCGCCTGCC	putative replication initiator protein
1052	*sav_4284*	*dnaB*	GCCGACAGGCCTAACGTGGTCCGGC<br>GCCGCGCGGACCTCGCC	putative replicative DNA helicase
1053	*sav_5676*	*dnaG*	GCCGCGTCGCGGAGACCCCGGC<br>GCCGCGGTCCGCC<br>GCCGCTGCGCGGTGGGCCGCC<br>GCCGCACAGCC	putative DNA primase
1054	*sav_4317*	*dnaN1*	ACCGCACTGACAGTCGTTGCCGCC<br>GCCGCCTCCTGCTATTGCCGGC	putative DNA polymerase III beta subunit
1055	*sav_2147*	*dnaQ3*	ACCGCGCCCGCC<br>ACCGCGTTCCGCTTCGCC	putative DNA polymerase III epsilon subunit
1056	*sav_4150*	*dnaZX*	ACCGCCCGAACGGCTCGCC	putative DNA polymerase III gamma and tau subunit
1057	*sav_2933*	*dndA*	ACCGAATCAGCC<br>ACCGCTAGCGCCGCC	putative DNA modification (L-cysteine desulfurase:

				pyridoxal phosphate-dependent)
1058	*sav_2932*	*dndB*	ACCGCTAGCGCCGCC<br>ACCGAATCAGCC	putative DNA modification (ATPase)
1059	*sav_4321*	*gyrB*	GCCGCAGCCGCTCCGGCCACCCGCC<br>ACCGCACGCGGTCGGCCCCGGC<br>GCCGCCCTCAACCGGTCTCGGC<br>GCCGACAGTGATGGGGGTTTGCC<br>GCCGATCTCCCGGC	putative DNA gyrase subunit B
1060	*sav_3915*	*helD1*	GCCGACCCGCCCTCGCGGCAGCC<br>GCCGCGAGGGCGGGTCGGC	putative ATP-dependent DNA helicase
1061	*sav_1978*	*helZ3*	GCCGATGACCGGCGACCAGGC<br>GCCGATTGTCAGACCCCGCC	putative SNF2/RAD54 family helicase
1062	*sav_5558*	*holA*	GCCGCGTCGCC<br>GCCGCCTCGCCCGGC	putative DNA polymerase III delta subunit
1063	*sav_4623*	*holB*	GCCGACCCGGC<br>GCCGACCGCGCTCAGCC	putative DNA polymerase III delta primer subunit
1064	*sav_4128*	*hrpA*	GCCGCAACGGACCCCCGCC<br>ACCGACCCCACCCCAGCCGCCCGGC<br>GCCGCCACGCACAAAAGCGTTCGCC	putative ATP-dependent helicase
1065	*sav_5127*	*hup*	ACCGAGGACCCTGGCAGCATCGGC<br>GCCGATGCTGCC	putative DNA-binding protein Hu 1
1066	*sav_2684*	*hupB*	GCCGCGACTGCC<br>GCCGCAACGAACTCGGC	putative histone-like DNA-binding protein
1067	*sav_159*	*int1*	ACCGCCCCTCACCGCC<br>ACCGCCCCCGCC	putative tyrosine-family recombinase/integrase
1068	*sav_497*	*int3*	GCCGCCCGGC<br>GCCGAACGCCGCCGCGACCAGGC<br>GCCGACGTCGACCGGC	putative recombinase/integrase
1069	*sav_4468*	*int11*	GCCGCCTCAGGC	putative serine-family recombinase/integrase
1070	*sav_5548*	*int13*	ACCGACTGAATTGGAGTCGGC	putative serine-family recombinase/integrase
1071	*sav_5555*	*int14*	GCCGCTGACCTGCTGTTTTGCTGCC	putative serine-family recombinase/integrase
1072	*sav_5815*	*int15*	GCCGACCGCCCCAGCC<br>GCCGCCGGGCGCGGCCAGCC<br>GCCGCGCCCGGCGGCGCGCTGGC<br>GCCGCCTCTGCGGCGCGGCCTGCC	putative tyrosine-family recombinase/integrase
1073	*sav_2500*	*lhr*	GCCGAGGCGGACCACCCGCCGCC	ATP-dependent DNA helicase
1074	*sav_1697*	*ligC*	GCCGATGTTCGCC	putative DNA ligase
1075	*sav_2504*	*mrgA2*	ACCGCCTCGCCCCGCCCCGGC	putative starvation-induced DNA protecting protein
1076	*sav_2664*	*mutM1*	ACCGAGCGCCCGCC	formamidopyrimidine-DNA

				glycosylase
1077	*sav_4707*	*mutY*	GCCGCGACGGGCCCGCACACCGGC<br>ACCGCACAGCCCCGCACCCGCC<br>ACCGCCCGCCCCACCCCGGC	putative A/G-specific adenine glycosylase
1078	*sav_2501*	*nei1*	GCCGCTCGCGCCACGTCCGGC<br>GCCGCTCTCTGGC	putative endonuclease VIII and DNA N-glycosylase with an AP lyase activity
1079	*sav_5427*	*nei2*	ACCGAAGAATCAGCCCGGCCGGC	putative endonuclease VIII and DNA N-glycosylase with an AP lyase activity
1080	*sav_4593*	*nth*	GCCGCACTGCC<br>ACCGACATCCGCC	putative endonuclease III
1081	*sav_2053*	*ogt2*	GCCGACCGGC<br>ACCGAGGACGCCGGTCGGC<br>GCCGCCCTGTCTGGC	methylated-DNA-protein-cysteine S-methyltransferase
1082	*sav_2423*	*parC*	ACCGCAGATTTCCTGCC	putative subunit of toposiomerase IV
1083	*sav_2442*	*parE*	ACCGAAGCCCGGC<br>GCCGCGAGCGGGAACGTTTTCGGC<br>GCCGCCAACCGGCAGCAATCAGCC	putative subunit of toposiomerase IV
1084	*sav_1212*	*phrB*	GCCGCACAGGC<br>GCCGCCCTGTGCCTGTCGCC<br>GCCGCTGGATCCCGACCGGCTGCC<br>GCCGACGGATCCCGCC<br>GCCGCGCCTGTGCGGCTCTGCC	putative deoxyribodipyrimidine photolyase
1085	*sav_4636*	*polA2*	GCCGCCTGCC<br>ACCGCGGGGCAGGC	putative DNA polymerase I
1086	*sav_6875*	*priA*	GCCGCCGCGGCACCCGGC<br>GCCGCGTCGTCGGC<br>ACCGCTCGGC	putative primosomal protein
1087	*sav_4710*	*radA*	GCCGACAGGTGGCCCCTTCTGCC<br>ACCGCCCGCC<br>ACCGCGACCGGC<br>GCCGCCACCCCGCGATTCGGC<br>GCCGCACATCAAGAAGCGCAGCC	putative DNA repair protein
1088	*sav_2492*	*recA*	GCCGCACACAGACCCCGGC<br>GCCGCGACCGGCGGATCAGGC<br>GCCGCCGACGCCCCACCCGTCAGGC	putative recombination protein RecA
1089	*sav_2671*	*recG*	ACCGCCCGACCCCGCGCCGTCGCC	putative ATP-dependent DNA helicase
1090	*sav_5628*	*recO*	GCCGATGCGCGGGCGCCGTAGGC	putative DNA recombination and repair protein
1091	*sav_4851*	*recQ*	GCCGACGGGCCGGGTCGGC<br>GCCGACCGGC	putative ATP-dependent DNA helicase

			GCCGACCCGGCCCGTCGGCTGCC<br>GCCGCTCAGCCGGTCGGC<br>GCCGCGTACCCGGC<br>ACCGACCGGC	
1092	*sav_3982*	*repSA2*	GCCGCCCCGGC<br>GCCGCCCCGGAGGTCTGGC	putative replication initiation protein
1093	*sav_1310*	*rho2*	GCCGCGACCGGC<br>GCCGCACCCGAGGTGTTTCCGCC<br>GCCGCCTCCGGC<br>GCCGAGAGCCATCTCATTCCCGCC	putative transcription termination factor
1094	*sav_6835*	*ruvB*	ACCGCAAGGCACCGGC<br>GCCGCGGGCCGGACCCCCGGCCGGC	putative Holliday junction DNA helicase subunit A
1095	*sav_6833*	*ruvC*	ACCGAAAGGAAACTCCCGCC<br>ACCGACAACGACGGTGGGCCGGC	putative Holliday junction nuclease
1096	*sav_3542*	*savM1*	GCCGACGTGCCCTTGCCGCC	putative type II restriction-modification system DNA adenine-specific methylase
1097	*sav_3309*	*savR1*	GCCGCACCGCTCGGGCACTGCC	putative Mrr restriction system protein
1098	*sav_1059*	*savR2*	GCCGCGCTGGCGCCATAGGC	putative Mrr restriction system protein
1099	*sav_7055*	*sbcD*	ACCGCCCGCCCGAACGCCCGTCGGC<br>GCCGACAGGC<br>ACCGACCACGCTAACGCCTGTCGGC	putative ATP-dependent dsDNA exonuclease
1100	*sav_6510*	*scpA*	GCCGATCCCCCCGCGCCGCCGGC<br>GCCGAGCAACCCGCGCCGTCGGC<br>GCCGAGCAACCCGCGCCGTCGGC<br>GCCGAGCACCCCGCGTCGTCGGC<br>GCCGAGCACCCCGCGTCTCCCGCC<br>GCCGCCTCCGCAGGCTCCGCC<br>GCCGACGACGCGGGACGACCGGC<br>GCCGACGGCGCGGGTTGCTCGGC<br>GCCGACGGCGCGGGTTGCTCGGC<br>GCCGACGACGCGGGACGACCGGC<br>GCCGACGACGCGGGGTGCTCGGC	putative chromosome segregation and condensation protein A
1101	*sav_2658*	*smc*	GCCGCGAGCGCGTCGTTCTCGGC	putative chromosome segregation protein
1102	*sav_5365*	*ssb2*	GCCGCGAGTTCAGACTGCC	putative single-strand DNA-binding protein
1103	*sav_6461*	*tldD*	GCCGCCCCGGC	modulator of DNA gyrase
1104	*sav_4621*	*topA*	GCCGCCGGACGGCCGCC<br>GCCGCCCGGCGTCGCCACCAGCC	putative DNA topoisomerase I
1105	*sav_7011*	*ung2*	GCCGATTGTCAGTGGTCGCC	putative uracil-DNA

				glycosylase
1106	*sav_4057*	*ushA*	ACCGCCTTGGCACAGCC	putative 5'-nucleotidase, secreted
1107	*sav_6286*	*uvrA1*	ACCGAACCGGC	putative excinuclease ABC subunit A
1108	*sav_2097*	*uvrA2*	ACCGCGAAGGCGGGCCCGCC<br>GCCGCGCGTCTGGGTATATTAGGC<br>GCCGCCTCGCTCGATCCGACCGGC	putative excinuclease ABC subunit A
1109	*sav_6291*	*uvrC*	ACCGCATTGAAGCGTGTTGCC<br>GCCGACCCCGGCATCGGCCGCCGCC<br>GCCGCGCTGTCAGCCCCCGCC<br>GCCGATGCCGGGGTCGGC	putative excinuclease ABC subunit C
1110	*sav_3463*	*uvrD1*	GCCGATCTCTTCCGGTCAGTCGCC<br>GCCGCACTCCCCCAGGCCCCGCC<br>GCCGCGTTACGGCCGGC<br>ACCGACAACCGGTCGCCGCC	putative ATP-dependent DNA helicase
1111	*sav_3077*	*uvrD2*	ACCGCTCTGCACGCGCGGCAGCC	putative ATP-dependent DNA helicase
1112	*sav_3072*	*uvrD4*	ACCGAAACAGCGACGCGTCCGCC	putative ATP-dependent DNA helicase
1113	*sav_3*		GCCGCCCATGAACCACCTCTGGC<br>ACCGACTACCGGC	putative endonuclease VII
1114	*sav_39*		GCCGCAGGGAGCGGCCCCGTCGGC<br>ACCGAGGCTCGCC<br>GCCGCACGCCGCTGCCGGC	putative terminal protein Tpg homolog
1115	*sav_273*		ACCGCATCGCC<br>ACCGCCGACTCCACCGCC<br>GCCGAACGCTTCCTCGCC<br>ACCGCAGACTCGCC<br>ACCGCCCGCCACCCGCGCACCGGC<br>GCCGCGCTGGC	putative DNA invertase/recombinase
1116	*sav_279*		GCCGAGACCAGCC<br>GCCGCAGGCGTGCGGGCCCGGC<br>GCCGAAGAGGGGGTTGCC	putative endonuclease
1117	*sav_501*		GCCGACTGCC	putative nuclease
1118	*sav_1131*		GCCGATCAAGACGTCAGCC<br>GCCGAGGCCATACGCCCCGGCCGGC<br>GCCGCCCACCCCGTGACAGCC	putative alkylated DNA repair protein
1119	*sav_1443*		ACCGAGCCGCAGATCGGC<br>GCCGATCTGCGGCTCGGTTAGCC<br>ACCGCCTCCTCGCCCATCGGC	putative DNA repair protein
1120	*sav_1696*		GCCGATGTTCGCC	putative DNA primase, small subunit
1121	*sav_2447*		GCCGCGATCATAGTCGTCTGCC	putative DNA hydrolase

1122	*sav_2450*		GCCGCACCCCGCCACATGTCGCC	putative ATP-dependent DNA helicase
1123	*sav_2928*		GCCGCCCGCC	putative DNA repair helicase
1124	*sav_3589*		ACCGCAAGGCCGGC<br>ACCGCAAGGCCGGC<br>GCCGCGCCCGGC<br>ACCGCCTCCCGCCGGC	putative deoxyribonuclease
1125	*sav_5202*		GCCGCACGACCAACGGGTCGCC	putative ATP-dependent helicase
1126	*sav_5386*		ACCGCTTCGCC	putative endonuclease
1127	*sav_6513*		GCCGCGCCTTGACAGCC	putative DNA hydrolase
1128	*sav_6714*		ACCGACAGGCACCCCGCC	putative 5'-3' exonuclease
1129	*sav_7440*		GCCGAAGCGGTTCGAGGACCGCC<br>GCCGCCCCGGCCCGGC<br>ACCGCCGTCGCC<br>ACCGCTTCGGC<br>ACCGCCGCACCCTGCTCACCCGGC	putative DNA polymerase beta chain
**RNA synthesis and modification (30)**				
1130	*sav_4027*	*dtd*	ACCGAGTGCCCCCGATCGGC	putative D-tyrosyl-tRNA (Tyr) deacylase-like protein
1131	*sav_4310*	*gidB*	GCCGAAGTTTGTCAGCC	16s ribosomal RNA small subunit methyltransferase
1132	*sav_3301*	*greA*	GCCGCTGAATTGTTAGGC	putative transcription elongation factor
1133	*sav_5260*	*orn*	GCCGCACCCGGCCCGGC	putative 3'->5' exoribonuclease
1134	*sav_6925*	*rbpA*	ACCGCCCTTTGGC<br>GCCGCCCCTTGTTGTACCCACCGGC	RNA polymerase-binding protein
1135	*sav_5329*	*recD*	GCCGACGGCGGACCGCC	putative exodeoxyribonuclease
1136	*sav_3098*	*rhlE*	GCCGACGTCCCCGGC<br>GCCGAGGCCCTGGAAGCCGTCGGC<br>GCCGAGTGGCATGGGACCACTGCC<br>GCCGACGGCTTCCAGGGCCTCGGC	putative ATP-dependent RNA helicase
1137	*sav_2642*	*rimM*	GCCGCCTTCGGC	putative 16S rRNA processing protein
1138	*sav_2231*	*rnd*	ACCGCATCCGCCGATCACACCGCC	putative ribonuclease D
1139	*sav_1138*	*rnhA*	GCCGAAACCTCCGCC	putative ribonuclease H
1140	*sav_2453*	*rnhB*	GCCGCACACCAGCC	putative ribonuclease HII
1141	*sav_5880*	*rnr*	GCCGCCCCCACCCGCCCCCGCC<br>GCCGCCAAAATCCGCGTCACCGGC<br>GCCGCCCTCGGC<br>GCCGCCACCCCCGGC<br>GCCGCCACCCCCGGC<br>ACCGCCACCGCC<br>GCCGCCCTGGC	putative ribonuclease R

			ACCGCCTCCCCTCCCGGC<br>GCCGCCCCACCGGC<br>GCCGCCCGCC<br>GCCGCCCGCCGGGACCTCCGGC<br>GCCGCCCGCC	
1142	*sav_5710*	*rphA*	GCCGCAGACGGGTGAGGGGCCGCC	putative ribonuclease PH
1143	*sav_4953*	*rpoA*	ACCGCTCCGAAGAGCCGTACCGCC	RNA polymerase alpha subunit
1144	*sav_440*	*rpoA2*	ACCGCCAGGCAGGC<br>GCCGAACCGCC	putative RNA polymerase alpha subunit
1145	*sav_4914*	*rpoB*	GCCGCCGGTGACAGGC	RNA polymerase beta subunit
1146	*sav_1444*	*rsmC*	ACCGCCTCCTCGCCCATCGGC<br>GCCGATCTGCGGCTCGGTTAGCC<br>ACCGAGCCGCAGATCGGC	putative ribosomal RNA small subunit methyltransferase
1147	*sav_6878*	*sun*	GCCGCGCCACCCAACTCGGC<br>GCCGCCCAACTCGGC<br>GCCGCCCAGCTCGGC	RNA-binding Sun protein
1148	*sav_4115*	*trmB*	GCCGCGCCCTGCTCGGGGGTCGGC<br>ACCGCTTGAAGGTCGCC<br>ACCGCCTCCCGGATACCGCC	putative tRNA (guanine-N(7)-)-methyltransfer ase
1149	*sav_6740*	*tsnR2*	GCCGACGCCGGC	putative rRNA methyltransferase
1150	*sav_3211*	*xseA*	GCCGCACCCGGC<br>GCCGAAGATCGCC<br>ACCGACCCGGC<br>GCCGCGTTGTCACAGGC<br>GCCGAACCGCCGGC	putative exoribonuclease, large subunit
1151	*sav_75*		ACCGCCGGCGCCCCCGGC<br>ACCGCCGCAGCC	putative endoribonuclease L-PSP
1152	*sav_992*		ACCGCGGGCCGGC	putative membrane protein, ribonuclease BN-like family
1153	*sav_1216*		GCCGAGCGGGTGCATCCGATCGCC<br>GCCGCGGCAGAACCGGC<br>ACCGCGATCGTCAGGAGCCACAGCC<br>ACCGCCACCGCCACCGCCACTGCC	putative membrane protein, ribonuclease BN-like family
1154	*sav_2122*		ACCGCCTGGC<br>GCCGCGAGCCCTGGCCTACCGGC	putative integral membrane protein, ribonuclease BN-like family
1155	*sav_4123*		ACCGCCTCTATGCGGTCCGGC	ATP-dependent RNA helicase
1156	*sav_4155*		ACCGCACGCGGACGCTCCGCC<br>GCCGACGCCGCC	ATP-dependent RNA helicase
1157	*sav_4446*		ACCGCACCACATCCCCGGC<br>ACCGCCCGCC	ATP-dependent RNA helicase
1158	*sav_6882*		GCCGCCGATGCTGTCACAGCC	putative guanyl-specific ribonuclease Sa3 precursor

				(RNase Sa3)
1159	*sav_7054*		GCCGCGTCGGCGCGGTCGGC	putative ATP-dependent RNA helicase
**Detoxification and adaptation to atypical conditions (52)**				
1160	*sav_825*	*aph1*	GCCGAGAGGCTCGCAGCC<br>GCCGACGCCCCTTGGC<br>ACCGCGTTTCCGCC	putative aminoglycoside phosphotransferase
1161	*sav_5999*	*arsC*	ACCGCTCGGC	putative arsenate reductase
1162	*sav_1302*	*axe1*	GCCGCGACCCGGC<br>ACCGATCGCCGGCCAACCGCC<br>GCCGACGTCGCGGGAACCCGGC<br>GCCGAGGGGGCCGCCAGGCCTTGCC	putative acetyl xylan esterase
1163	*sav_1261*	*blaA1*	GCCGCAGAATGCCGCAGCC	putative beta-lactamase, secreted
1164	*sav_5764*	*blaA4*	GCCGCTCCTCTGCCGGCAGCC	putative beta-lactamase
1165	*sav_5791*	*blaA5*	GCCGCCCCTGTCCGCCACTTAGGC<br>GCCGCGGGCAGTCTCCGCC<br>GCCGCATCGAGGGGTCGGGCTCGCC	putative beta-lactamase
1166	*sav_660*	*blaB1*	GCCGACCCGCC<br>GCCGCCCTTCGTGACCGTTGCC	putative metallo-beta-lactamase
1167	*sav_5568*	*blaB3*	ACCGCCCTTGCC<br>GCCGCCCCGCCCGGC	putative metallo-beta-lactamase
1168	*sav_4241*	*copC*	GCCGCTTCGCGAGGCAATTCCGGC	putative copper resistance protein, secreted
1169	*sav_1322*	*crcB2*	GCCGCGATTCTTGCC<br>ACCGCGGCACCGCGGCCGCC	putative camphor resistance protein CrcB
1170	*sav_3907*	*cspB1*	GCCGCGCGCCCGGC<br>GCCGCCGGACCATTAGCC<br>ACCGAATTCCGCC	putative cold shock protein
1171	*sav_3932*	*cspB2*	GCCGCGGTCGACGCGTTCCGGC	putative cold shock protein
1172	*sav_826*	*cspD1*	ACCGACGGCGACCCGGC<br>GCCGCGTAGCCAGGC<br>ACCGATCCGCAGTCGCCTGGC	putative cold shock protein
1173	*sav_893*	*cspD2*	GCCGACAGGCGGTAGCC<br>GCCGAGACGGCAGTCCTGCGCCGCC<br>GCCGCACCCCGCTGGC<br>GCCGCGCACCGGCTACCGCC	putative cold shock protein
1174	*sav_4154*	*cspD3*	ACCGCGTTCCCGGC<br>GCCGCGTCGCCGGCACGCCCTGCC<br>ACCGCGGCCACAGATATGGGTCGGC	putative cold shock protein
1175	*sav_4447*	*cspD4*	GCCGCACTAGATATCTGGCCCCGGC	putative cold shock protein
1176	*sav_4776*	*cspD5*	GCCGCGGTGCCACCGCCGCC<br>GCCGCCCCGTTCCCCTCGCC<br>ACCGCGGCGGGAACGAGGCCGGC	putative cold shock protein

			GCCGCGATGATTGCCCTGCC	
1177	*sav_1622*	*gbsA1*	GCCGCACGTCCCGCTCCCGCC<br>GCCGCTCCGGCCCGGC<br>GCCGACCTGCCCTCGGGTTCAGGC<br>GCCGAAGCCGTCCTACCGCC	putative glycine betaine aldehyde dehydrogenase
1178	*sav_3434*	*gbsA2*	GCCGCGACACGGACGAGTCGCC<br>GCCGCTCGGC<br>GCCGCACACTTTCGGC	putative glycine betaine aldehyde dehydrogenase
1179	*sav_2672*	*htpG*	ACCGCCCGACCCCGCGCCGTCGCC	putative heat shock protein, Hsp90-family
1180	*sav_6003*	*htpX2*	GCCGCAGCGGAAAGTCCGGC<br>GCCGACAGCC<br>ACCGCCATTAAAGGCTAGCCGCC<br>GCCGACAGCC<br>GCCGCAATCCGGGGGTACACCGGC	putative heat shock protein, protease
1181	*sav_3961*	*hur*	ACCGCCCACCCGGGACCTCGCC	putative hydroxyurea phosphotransferase
1182	*sav_3224*	*katA2*	GCCGACGTCCCGTCGGC<br>GCCGACGGGACGTCGGC	putative catalase
1183	*sav_348*	*katB*	GCCGCGCGGCGCCCGCC<br>GCCGCGCAGCCGGAAATGAATCGCC<br>GCCGCACTCTCTTCGGC<br>GCCGCCTTCTCATATTTCGCC<br>ACCGCGACCGTGGCCGGC	putative catalase
1184	*sav_3587*	*ksgA*	GCCGCCGCTGCC<br>ACCGAGCCGCCGTGAGGCTGGC<br>GCCGCACCGCGGCACGCGCCCCGGC<br>ACCGCGGCACGCGCCCCGGC<br>ACCGCGGCACGCGCCCCGTCCGCC<br>ACCGCAGCCCCGCC<br>GCCGCCGCCCAGGC<br>GCCGAGGCCGCCGCC	putative dimethyladenosine transferase
1185	*sav_299*	*lipW*	GCCGACTCCTGCGCGACAGCC<br>GCCGAAGTGGCCGACCAGGTCGCC<br>ACCGCGGCCGACCGGC	putative secreted esterase/lipase
1186	*sav_3790*	*mdmB*	ACCGCCGGTGCTCGCC	putative macrolide O-acyltransferase
1187	*sav_1605*	*mrgA1*	ACCGCCCGCC<br>ACCGACGGCGGGCGGTGCACCTGCC	putative starvation-induced DNA protecting protein
1188	*sav_4058*	*mshD*	ACCGCCTTGGCACAGCC	putative N-cysteinyl-1-D-myo-inosityl-2-amino-2-deoxy-alpha-D-glucopyranoside acetyltransferase
1189	*sav_1531*	*nos*	ACCGCGGAGCCGCC	putative nitric oxide synthase

1190	*sav_3610*	*ohrB1*	GCCGCGGGTTACCCGCGGTTGCC<br>ACCGCGAACGGGCGCCTACCGGC<br>GCCGCTGTCGACACCCGCC	putative organic hydroperoxide resistance protein
1191	*sav_1862*	*osmC*	GCCGAGTGCGACTCCGCC<br>GCCGAACCGGC	putative osmotically inducible protein
1192	*sav_4171*	*pacB2*	ACCGATGATCGCGCCTACCGGC<br>GCCGCAATCGAAGTTAGGTTAGCC<br>ACCGCGGGGAACCGGC	putative penicillin acylase
1193	*sav_6608*	*pacB3*	ACCGCCATAAGCCACCCGCC<br>GCCGCTGTCAGCCGCCTGTCGGC<br>GCCGCCGACAGGC	putative penicillin acylase, secreted
1194	*sav_7231*	*pfpI2*	ACCGAGGCCCGGGTCGCC	putative intracellular protease/amidase
1195	*sav_2523*	*pnp*	ACCGAGGACCGGC	putative guanosine pentaphosphate synthetase/polyribonucleotide nucleotidyltransferase
1196	*sav_1427*	*terA*	GCCGAGTTCCTGGCGGGTTACCGGC<br>ACCGACAGACTGCC<br>ACCGCCGGTAACCCGCC<br>ACCGAGACCCACCGGAAGATCGCC<br>GCCGCCGCGCCAGGC	putative tellurium resistance protein
1197	*sav_896*	*terD1*	GCCGCCAGGACGGATCGGC<br>ACCGCCCGCTTCGCCTACAGGC<br>GCCGATCCGTCCTGGC<br>GCCGCCACACTGGCCGTCGGC	putative tellurium resistance protein
1198	*sav_5804*	*terD4*	GCCGAAGACCGCCCGGC	putative tellurium resistance protein
1199	*sav_6274*	*terD6*	ACCGCCCCGTCCGACCGCC<br>GCCGAACCCCGGGCCGCC	putative tellurium resistance protein
1200	*sav_7507*	*tetM*	GCCGCGAGCGGCGCCGGATCGCC	putative tetracycline resistance protein
1201	*sav_737*		GCCGCAACCCACGACCGGC<br>GCCGACCATCGCGCCGCC	putative 2-haloalkanoic acid dehalogenase
1202	*sav_1149*		GCCGAAGGGGAAGGGATCGGC<br>GCCGATCCCTTCCCCTTCGGC<br>GCCGAGGCGATGGGCCGGC	putative secreted esterase
1203	*sav_1260*		GCCGCAGAATGCCGCAGCC	putative dioxygenase
1204	*sav_1275*		GCCGAAGTCGAAGACGATCGCC	putative stress-inducible protein
1205	*sav_1595*		GCCGCGTGCTCGGC<br>GCCGCCGACGCGGCCGACCTGCC<br>GCCGCCGACACCGGGGAGCCCGGC<br>GCCGCGTCGGC	putative 3-ketosteroid-delta-1-dehydrogenase

1206	*sav_2113*		GCCGCCGACCCGACCGTCGCC<br>GCCGCCCGAACGGATATGTGTCGGC	putative carboxylaseterase, secreted
1207	*sav_2605*		GCCGCGCGGCTCAGACCTTCAGCC<br>ACCGCGTCGACAGCCCCATAGCC	putative dioxygenase
1208	*sav_5004*		ACCGCCCGCCGCC<br>GCCGCTACACGGAGGGACCGCC	putative beta-lactamase, secreted
1209	*sav_5965*		GCCGCAGTGCAGGC<br>GCCGCTGCGCGGGCTCCGCC	putative hydrolase
1210	*sav_6193*		GCCGCCGACCCCCACCGGC<br>GCCGAGGGTATGTCACAGCTGCC	putative toxic cation resistance protein
1211	*sav_6951*		GCCGCTTCCCCCAACCCCTGCC	putative sarcosine dehydrogenase
**Cell wall and cell envelope (20)**				
1212	*sav_3413*	*dacB*	GCCGACGCTCCCCACCCCGGC	putative D-alanyl-D-alanine carboxypeptidase
1213	*sav_3781*	*dacC*	ACCGAACCGTGAATCCTCGCC	putative D-alanyl-D-alanine carboxypeptidase
1214	*sav_4379*	*dacD*	GCCGCCGCAGTGATCACTGCCGGC	putative D-alanyl-D-alanine carboxypeptidase
1215	*sav_4662*	*dacE*	ACCGCGACTGGCGCGGACCGCC<br>ACCGCGACCGGC	putative D-alanyl-D-alanine carboxypeptidase
1216	*sav_3561*	*glmU*	GCCGAGCATCGCTCCCCCGCC	UDP-N-acetylglucosamine pyrophosphorylase
1217	*sav_5128*	*murA1*	GCCGAGGCGCTCGGC<br>GCCGAGCGCCTCGGC	UDP-N-acetylglucosamine transferase
1218	*sav_2260*	*murA2*	GCCGCCCAACTGGCCTGCC	UDP-N-acetylglucosamine transferase
1219	*sav_5214*	*nagD*	GCCGCCGACCGCCGCC	putative N-acetyl-glucosamine catabolism protein
1220	*sav_3393*	*nagZ2*	GCCGAGGACCCCCGCTGCC	beta-N-acetylhexosaminidase, secreted
1221	*sav_5268*	*nagZ4*	ACCGCCTGCTTGGC<br>GCCGCGCCTGCACCCCTGCC<br>ACCGCGCCGCTCCCGCC<br>GCCGCAGGAACCAGCCAAGCAGGC	beta-N-acetylhexosaminidase, secreted
1222	*sav_5302*	*nagZ5*	ACCGACTTCCCGGC<br>GCCGCACGCGGCCGGC<br>GCCGCCCCGGCTCGGC<br>ACCGCACCGCGGAGACTCATCCGGC	beta-N-acetylhexosaminidase, secreted
1223	*sav_4294*	*pbp5*	ACCGATTCGTAGGAAGCAAGTTGCC<br>ACCGAAGTTCACCCCAAATCAGGC	putative penicillin-binding protein
1224	*sav_5179*	*pbp9*	GCCGCAGCCGCAGCAGAGCAGCC<br>ACCGCTCCGGACACCGCC<br>GCCGAAACGGCCCAAGCGCACCGGC	putative penicillin-binding protein

			GCCGCCAGGTCGGGATCAGCCGGC	
1225	*sav_5458*	*pbp10*	ACCGCCCGTGTCCACAGGTTCGCC	putative penicillin-binding protein
1226	*sav_7219*	*pbp12*	GCCGCGAGCAGGGTCAGGC<br>ACCGATTCGGC<br>ACCGCGAGCCCCCCTGCC	putative penicillin-binding protein
1227	*sav_2311*	*tagE*	GCCGCCGCGGCCATAGGC	UDP-glucose:polyglycerol phosphate glucosyltransferase
1228	*sav_444*	*wapA*	GCCGCTGGGTGGACCTGGC	putative cell wall-associated protein
1229	*sav_4989*		GCCGAGGTGTCGGCCCGGC<br>ACCGACTGGC<br>GCCGACACCTCGGC	putative polysaccharide deacetylase, secreted
1230	*sav_6420*		GCCGCGATCCTGCCAGGC<br>ACCGATACATATCGCC<br>GCCGCACACGTCGCC	putative lysozyme precursor, secreted
1231	*sav_7297*		GCCGACTCGGC<br>GCCGACCGCACCGGC<br>GCCGAGTCGGC	putative oligosaccharide deacetylase, secreted
**Cell division and differentiation (25)**				
1232	*sav_4026*	*amfC*	ACCGAGTGCCCCCGATCGGC	putative AmfC protein
1233	*sav_2529*	*bldB*	ACCGAGGGATCAGCC	putative BldB protein
1234	*sav_3007*	*eshA*	ACCGCGCGAAGGAACAGGC	putative nucleotide-binding protein
1235	*sav_5104*	*ftsE*	GCCGCTGCCCTGTCTGCC<br>GCCGAAATCCGCGGAACCTGGC	putative cell division ATP-binding protein
1236	*sav_4666*	*ftsH*	GCCGCAGGGGACTGCC<br>GCCGCCCCGTCCCTCCTGCC	putative cell division protein FtsH
1237	*sav_2510*	*ftsK*	GCCGAAGCCCGTCGCC	putative DNA translocase FtsK
1238	*sav_6123*	*ftsQ*	GCCGCCGCCGCGGCGACCGCC<br>GCCGCGGCAGATGTACTGGC<br>GCCGCGGCTTTGGCGGTCGCC<br>GCCGCGTGGTCCGCC	putative cell division septal protein FtsQ
1239	*sav_6121*	*ftsW1*	GCCGCCTCGCC<br>ACCGCTCAGGC	putative cell division membrane protein FtsW
1240	*sav_2654*	*ftsY*	GCCGAGGGACATAGGC	putative signal recognition particle receptor
1241	*sav_6124*	*ftsZ*	ACCGCCGATAGGC	putative cell division GTPase FtsZ
1242	*sav_3255*	*minD1*	GCCGCGATGCGCTCAGCC	putative septum site determining protein
1243	*sav_4605*	*minD2*	GCCGATGCGAACCGGC	putative septum site determining protein
1244	*sav_5455*	*mreB1*	GCCGCCCCAGGTCGGC	putative rod shape-determining

				protein
1245	*sav_5720*	*mreB2*	GCCGACGGTGTGATCCTCGGC<br>GCCGATCCACCCGTCCGGC<br>ACCGCCGCCCGGC<br>GCCGAGGATCACACCGTCGGC<br>GCCGCCGGACGGGTGGATCGGC	putative rod shape-determining protein
1246	*sav_4309*	*parA1*	GCCGAACCCTGGCGACACTCCGGC	putative partitioning or sporulation protein
1247	*sav_6508*	*parA2*	GCCGACACATCGGGTCGGGTCCGGC<br>GCCGACGATTGGC<br>GCCGACAGCC	putative partitioning or sporulation protein
1248	*sav_6650*	*parJ*	GCCGAGTGCACACCGGC<br>GCCGCCTGCC	putative protein interacts with ParA
1249	*sav_4113*	*sap*	GCCGAGCACGCTCCGTACAGGC<br>GCCGCCCGCGGGGATGTAGGC<br>GCCGCGCTGGC	putative sporulation associated protein, secreted
1250	*sav_5140*	*sdrA*	GCCGATCCGGCCCGCGCCGCC<br>GCCGAGCCGCCTGACCCCCCTCGCC<br>GCCGCCCGCCCGTCCCCCCACCGCC	putative protein involving differentiation
1251	*sav_5142*	*sdrC*	GCCGAACCTCGCCCTCGCCGCC<br>GCCGCGTACGCCGGGCATCGGC<br>ACCGAGCCGCCGCC<br>ACCGACTGCGCTCTTCTTCGGC<br>GCCGATGCCCGGC<br>GCCGCAGCGCGTCCGGC	putative secreted protein
1252	*sav_1431*	*spoVK*	GCCGAACTGTTCGGC	putative sporulation protein K-like protein
1253	*sav_6810*	*ssgC*	GCCGAGCCCCCGCTCGGC	putative cell division protein (probably reverse function of SsgA)
1254	*sav_580*	*ssgZ*	ACCGCCCAGGC	putative SsgA homolog (the deletion was not affected on the sporulation)
1255	*sav_5446*	*tig*	GCCGCACTGCC<br>GCCGACTCGGC<br>GCCGCATTTCGCGGGCTCCGGC<br>GCCGAGTCGGC	putative cell division trigger factor
1256	*sav_2528*		ACCGCGGACGTCCGCC<br>GCCGCGACCCCGCGGGGTCGGC<br>GCCGCTCACCTCGAAGTGACCGGC<br>GCCGCGGATCGGC<br>ACCGAGTTGTTCCGTCCGCCGCC	putative FtsK/SpoIIIE family protein
**Transport and binding proteins (213)**				
1257	*sav_7503*	*amfT*	ACCGCGGGACCGGCGGACCGGC	putative membrane translocator

			ACCGAGCCTTCCAGCC	
1258	*sav_1943*	*amiS*	GCCGCATCCCCGGCAGCTCACCGGC	putative acetamide transporter protein
1259	*sav_2651*	*amtB1*	GCCGCGGCCGCCATCCCGAACCGCC<br>GCCGCGGCCGGTTTTCTGCC<br>ACCGCTCAGGTTTCGCC<br>GCCGCGGCTGCC	putative ammonium transporter
1260	*sav_1677*	*ansP2*	ACCGCGTCGCCGCC	putative L-asparagine permease
1261	*sav_2982*	*aotJ*	GCCGACGAGATGTCTCCTGGC<br>ACCGAAGATCTTCCGGC<br>GCCGATGGCCGGCGTTGCTGCC	putative arginine/ornithine ABC transporter substrate-binding protein
1262	*sav_7263*	*araN*	GCCGCGCACCCGCC<br>GCCGCCCCACCCGCC	putative L-arabinose ABC transporter substrate-binding protein
1263	*sav_7477*	*arsB*	GCCGACCGGCCCCGGC	putative membrane efflux protein
1264	*sav_2887*	*atpB*	GCCGCGCCGACACATTGAGGTTGCC	F-type proton-transporting ATPase A chain
1265	*sav_3176*	*bldKA2*	ACCGATAGGC<br>GCCGCCCTCGCC<br>GCCGAGCCGGTGGCCGCCACAGGC	putative peptide ABC transporter permease protein
1266	*sav_3152*	*bldKC1*	GCCGCCCCACTCCTTCGGCCCGCC	putative peptide ABC transporter permease protein
1267	*sav_3174*	*bldKC2*	GCCGCCCGCGTCGCTGAGCAGGC	putative peptide ABC transporter permease protein
1268	*sav_5256*	*cebE*	GCCGAAAAGGCCTCATCGGC<br>GCCGACGGGCCTGAATACCGCC<br>GCCGATAACTGAACGCATCTCGCC<br>GCCGCACCGGGGCCGGTCGCCGGC<br>GCCGATGAGGCCTTTTCGGC<br>GCCGACCCCCCGGC	putative cellobiose ABC transporter substrate-binding protein
1269	*sav_6277*	*chaA*	ACCGACCCGAAGAAGTGCCCGGC<br>GCCGACTGCCCGGCCGCC	putative ionic transporter integral membrane protein
1270	*sav_2036*	*corA4*	GCCGAGGCCGTTTGCC	putative metal-transport protein
1271	*sav_877*	*cpt*	GCCGCACGGGCTGCCGGAGCAGCC<br>GCCGCGCTCCCGTGAACTCAGGC	putative chloramphenicol 3-O phosphotransferase
1272	*sav_3022*	*dasA1*	GCCGACCTGTCCGATCGGC<br>GCCGCTTCGGC	putative multiple sugar ABC transporter substrate-binding protein
1273	*sav_5131*	*dasA2*	ACCGCTCCCCCGCGCGCCCTGCC	putative multiple sugar ABC transporter substrate-binding protein
1274	*sav_7329*	*dasA3*	GCCGACGTCTCGCGCTCCGCC	putative multiple sugar ABC transporter substrate-binding

				protein
1275	*sav_5139*	*ebrB*	GCCGCACGGCCGTCGCC	putative multidrug resistance efflux protein
1276	*sav_6334*	*emrE*	GCCGCACACGTCCTGCC	putative SMR-type multi-drug efflux transporter
1277	*sav_601*	*fecD1*	GCCGCCTGCGGCTGCCGCTCGCC<br>GCCGACGACGGCGAGCGGCAGCC	putative ABC transporter iron (III)/siderophore transport system ATP-binding protein
1278	*sav_6491*	*fepD*	GCCGCCTGCCGGC	putative ABC transporter iron (III)/siderophore permease protein
1279	*sav_5704*	*frcB*	GCCGAGAGAGAGCAGCC<br>GCCGCGTCGCC	putative fructose ABC transporter substrate-binding protein
1280	*sav_5703*	*frcC*	GCCGCCCCCTAGGC<br>ACCGCCCCCTTGTCCCGGC<br>GCCGACCGCC	putative fructose ABC transporter permease protein
1281	*sav_3688*	*fruA*	ACCGCACCACCCCCCTCGGC	putative fructose-specific permease
1282	*sav_6964*	*glpF2*	ACCGAGGCCGGC<br>GCCGCATTCGGGCGTCTCCAGGC<br>GCCGCAAAAGAGGACACGCTCGGC	putative glycerol uptake facilitator protein
1283	*sav_7200*	*glpF3*	ACCGCGCCCCTCGGC<br>GCCGCTTCGAGCCCTTGGC<br>GCCGCGTTCGCC<br>GCCGAGGGGCGCGGTGGCCCCGCC<br>GCCGCCCGCC<br>GCCGCGCATCGTCGGC<br>ACCGCATTTGCC	putative glycerol uptake facilitator protein
1284	*sav_6400*	*gltI2*	GCCGATCCCGCCGCC<br>GCCGCGGCGGCGGGATCGGC	putative polar amino acid ABC transporter substrate-binding protein
1285	*sav_7208*	*gltK3*	ACCGCACAGCGGCGCCCCCGCC<br>ACCGCCCCGGGCTCTGCCCGCC<br>GCCGCCGCGGTGTGCTCTCCGCC<br>GCCGCCGTGTGCCGTCGCGCTCGCC	putative polar amino acid ABC transporter substrate-binding protein
1286	*sav_1963*	*gluA1*	GCCGCAAACTATCCAGCC	putative glutamate ABC transporter ATP-binding protein
1287	*sav_3277*	*gntP*	GCCGCGATCCCCCCTGCC	putative low-affinity gluconate transporter
1288	*sav_6283*	*gtlL2*	GCCGCCGGCCTTTCGACCCCCGCC	putative polar amino acid ABC transporter substrate-binding protein

1289	*sav_7151*	*iolTA*	GCCGCGTCATCGGC<br>ACCGCACCAGGC	myo-inositol ABC transporter substrate-binding protein
1290	*sav_919*	*kdpA*	GCCGACCTGGC<br>ACCGCCGAAAACGTCGTCGGC<br>ACCGCCTCTCTGGC<br>GCCGACGACGTTTTCGGC	putative potassium-transporting ATPase subunit A
1291	*sav_2636*	*lepB1*	GCCGATCTTCCGCGGCCCGGC<br>GCCGCGGAAGATCGGC	putative signal peptidase I
1292	*sav_6164*	*livF2*	GCCGACCCTGCC<br>GCCGCGCCGGGCGGACGGCCCCGGC<br>GCCGCTCCAAGAGGCAGGGTCGGC<br>ACCGAGCAGGC<br>GCCGCGTCCCGCGCGGAGCCCGGC<br>GCCGCGTCTCGGGACCGGC	putative branched-chain amino acid ABC transporter ATP-binding protein
1293	*sav_6223*	*livH3*	GCCGCGCGGCTGGC	putative branched-chain amino acid ABC transporter permease protein
1294	*sav_6224*	*livK3*	GCCGCCTTGCC<br>ACCGCGCCAGGC<br>ACCGACCGCC<br>ACCGCGCCGCC	putative branched-chain amino acid ABC transporter substrate-binding protein
1295	*sav_5047*	*lldP*	GCCGACTACTCCAGGCCGCC	putative L-lactate permease
1296	*sav_2577*	*lplA*	ACCGCTTCCGCTCAGCC<br>ACCGCAGCCGACGGCTCTTCCGCC	putative multiple sugar ABC transporter substrate-binding protein
1297	*sav_1456*	*lplB*	ACCGATAATTTCCGGC	putative multiple sugar ABC transport permease protein
1298	*sav_5977*	*malE*	GCCGACAGTTACCCCGCC<br>GCCGCTCCTGCCGGCTTGCC	putative maltose-binding protein
1299	*sav_5979*	*malG*	GCCGCAGGAGCCGCC	putative maltose permease
1300	*sav_836*	*matE*	GCCGAGATCAGAGGTAGATCGGC<br>GCCGATCTACCTCTGATCTCGGC<br>GCCGACTGCATCGACAAACCGGC	putative efflux family protein
1301	*sav_5902*	*mlaF*	ACCGCCGGAAAAGGCACTGCC<br>GCCGCCGAGCTGCCGGAAACCCGGC	putative ABC transporter ATP-binding protein
1302	*sav_4979*	*msmE*	ACCGATAATTATCGGC	putative multiple sugar ABC transporter substrate-binding protein
1303	*sav_1425*	*murI*	GCCGCAGTCTGCC	putative glutamate racemase
1304	*sav_5119*	*narK*	ACCGCTGGAGCTCCGGC	putative nitrate extrusion protein
1305	*sav_2251*	*ngcE*	GCCGACTGTGTGTTGCC	putative ABC transporter substrate-binding protein

1306	*sav_4598*	*nhaA*	ACCGACCCGGAGGTTCCCGCC<br>GCCGACCAGACTTCCCGGC	putative sodium/proton antiporter
1307	*sav_560*	*nicT1*	ACCGACTTCGTCCGGC<br>ACCGACGAGAGCCGCGGCCGGC<br>GCCGATTCCGACGAGCAGGC<br>GCCGCGCAGCAGCC	putative high-affinity nickel-transport protein
1308	*sav_2333*	*nicT2*	GCCGCTCCGGC	putative high-affinity nickel-transport protein
1309	*sav_5941*	*oleC4*	GCCGCAGGAACGCGTGCTCCTCGGC<br>GCCGCCGCACCCCTCAGGGGCCGGC<br>GCCGCCCCCGGTACGCCGGC<br>GCCGACTGGCGCCGACAGCCCGCC<br>ACCGACAAACCACCGACAGGC<br>GCCGCCAAGGTGTCAGCC	putative ABC transporter ATP-binding protein
1310	*sav_2768*	*oppA1*	ACCGCTCCCACGACTGCCGTAGCC	putative peptide ABC transporter substrate-binding protein
1311	*sav_3048*	*oppA2*	ACCGCGCATTCACCCCGCC	putative peptide ABC transporter substrate-binding protein
1312	*sav_5619*	*oppA8*	ACCGAACTTAGGTTAGGC<br>GCCGAACTCCTGCTGCC<br>GCCGCCCGCC<br>GCCGCATCCCCCCGCCGGC	putative peptide ABC transporter substrate-binding protein
1313	*sav_2767*	*oppB1*	ACCGCTCCCACGACTGCCGTAGCC	putative peptide ABC transporter permease protein
1314	*sav_3047*	*oppB2*	GCCGCCGCTCGGC	putative peptide ABC transporter permease protein
1315	*sav_5620*	*oppB8*	GCCGCCCCGCC<br>GCCGCCGCTCGCC	putative peptide ABC transporter permease protein
1316	*sav_2769*	*oppC1*	GCCGCGCCCCTGGC<br>GCCGCATCATCTGCGGTCGCC<br>GCCGCAGACAGCGGCGGGCGTAGGC<br>ACCGCCGCCAGGGGCGCGGCAGCC	putative peptide ABC transporter permease protein
1317	*sav_6715*	*opuAA*	ACCGACAGGCACCCCGCC	putative glycine betaine ABC transport ATP-binding protein
1318	*sav_3505*	*opuBA1*	ACCGCCAGGC	putative ABC transporter ATP-binding protein
1319	*sav_2178*	*opuBC1*	ACCGACCGAGAACCGGC<br>ACCGATTGGC	putative ABC transporter substrate-binding protein
1320	*sav_6386*	*pbuG*	ACCGCCGGGTACGAGGAGTCTCGCC	putative hypoxanthine/guanine permease
1321	*sav_3897*	*pbuG1*	ACCGCCGGAGCCGCC	putative hypoxanthine/guanine permease

1322	*sav_2016*	*pbuX2*	GCCGAGGGGGACGGCCCTCGGC<br>GCCGAGGGCCGTCCCCCTCGGC	putative xanthine/uracil permease
1323	*sav_2591*	*potD2*	ACCGCCGGGTGGCACCACCGGC	putative polyamine ABC transporter substrate-binding protein
1324	*sav_1125*	*potD3*	GCCGCACCCAGCATCAAGGCCGCC	putative polyamine ABC transporter substrate-binding protein
1325	*sav_4075*	*pstB*	GCCGCGTCAGCGGCCCGCC	putative phosphate ABC transporter ATP-binding protein
1326	*sav_4073*	*pstC*	GCCGCACCGTCCGGTGCACCGCC	putative phosphate ABC transporter permease protein
1327	*sav_4072*	*pstS*	GCCGCCACCAGTCGGC<br>ACCGCGTAAGGCCGAAATTAGGC	putative phosphate ABC transporter substrate-binding protein
1328	*sav_6974*	*ptsP*	GCCGCCGTGATGATTGTCTGCC	putative phosphoenolpyruvate-protein phosphotransferase
1329	*sav_7417*	*rhaP*	GCCGACAGCCGCCTGGAGGGCCGCC	putative simple sugar ABC transporter permease protein
1330	*sav_5071*	*secA1*	GCCGCTGACAGCC	putative preprotein translocase SecA subunit
1331	*sav_4908*	*secE*	ACCGCATCCGCC	putative preprotein translocase SecE subunit
1332	*sav_1560*	*ssuA2*	GCCGCACACCCCACCCCTTCAGCC	putative ABC transporter substrate-binding protein
1333	*sav_3044*	*sugE*	ACCGCTTTTTCTGGC	putative SMR-type multi-drug efflux transporter
1334	*sav_6692*	*tatA*	ACCGATCGGACAGCACCCCCGCC<br>ACCGAATCCATAGAAGCCGGC	putative sec-independent protein translocase protein TatA
1335	*sav_2391*	*trkA*	GCCGCCGCCGGACTCGTCGGC<br>GCCGACGAGTCCGGC	putative potassium transporter
1336	*sav_2247*	*xylF*	GCCGAGTTGACTTCCAGCC	putative simple sugar ABC transporter substrate-binding protein
1337	*sav_2246*	*xylG*	ACCGCGTACGGGAATTCGCC<br>GCCGCCTGACCCGGC	putative simple sugar ABC transporter ATP-binding protein
1338	*sav_6836*	*yajC*	GCCGATGCCGCC<br>GCCGCCCACAACGGCGGCATCGGC	putative preprotein translocase YajC subunit
1339	*sav_62*		ACCGCCGCCCACCCTCCGCC	putative transmembrane transport protein

1340	*sav_92*	GCCGACGGGGCGAGCCCGGC<br>GCCGCCACCACCGCC<br>GCCGCGGTGGCCGCC<br>GCCGCGGCGCTGAGGACAGTTCGGC<br>ACCGCCGTGCTCGCC<br>GCCGACGCCGGC<br>GCCGATCAGCCCGCC<br>ACCGCCGCGCCGCC	putative lipoprotein
1341	*sav_133*	GCCGCTCGCC<br>ACCGCTGCCGCCGTCGGTATCCGGC	putative transporter
1342	*sav_145*	GCCGACTTTCGGACGGTCCTGCC	putative membrane transport protein
1343	*sav_245*	GCCGCGGGGCTGGC<br>GCCGCGAGGGCCCGCCTGCC	putative LysE-family efflux protein
1344	*sav_252*	ACCGCGGCACGACCCTGGCCCCGGC<br>ACCGCCGTACCCGCGCTGGC<br>GCCGACCAGGGCAACGACTTCGCC	putative ABC transporter ATP-binding protein
1345	*sav_257*	GCCGCCCTAGTCACCGTGACCGCC<br>GCCGATCTGCCCCAGGC<br>GCCGCCTGGGCGGCACCTGGC<br>GCCGCGCACGCCTGGGTCGCC<br>GCCGCCCAGGC	putative ABC transporter ATP-binding protein
1346	*sav_552*	GCCGCTCGGC<br>GCCGAAGGAGTACCGGC<br>GCCGAGCGGCAGGC	putative transmembrane efflux protein
1347	*sav_695*	ACCGCCGGCGGTGGTGTCGCC	putative ABC transporter ATP-binding protein
1348	*sav_794*	ACCGCTACGGCTAACCCCAGGC<br>GCCGCTGAACGATTCCCGGC	putative amino acid transporter
1349	*sav_874*	GCCGCGGGGCCCGCC<br>GCCGAGGCAGCGGAACAGCC	putative solute-binding dependent transport lipoprotein
1350	*sav_891*	ACCGATCGCC<br>ACCGCTCCTCGTTCGGC	putative quaternary amine transporter
1351	*sav_933*	ACCGCTCGACCATATCCAGGC<br>GCCGCTCCGCC<br>GCCGCTCCTCCTGCAGCC	putative ABC transporter ATP-binding protein
1352	*sav_970*	ACCGACATCCACACGTCCGCC<br>GCCGCACCGGC<br>GCCGCTGCCTGCC	putative simple sugar ABC transporter ATP-binding protein
1353	*sav_1078*	GCCGCGCCCGGCCTCCCGCC<br>GCCGCAGCAGCC<br>GCCGCGGCGCCAGACAGGC	putative multiple sugar ABC transporter permease protein
1354	*sav_1133*	GCCGCCGGCGGCCGGC	putative membranse transport protein

1355	*sav_1159*	GCCGATCAGGC	putative ABC transporter ATP-binding protein
1356	*sav_1162*	ACCGCAGATTCCGCC	putative ion channel subunit
1357	*sav_1221*	GCCGAGGGGAGCCCGGCGCCGGC<br>GCCGCCCTTGATCCGCCGGCCGCC<br>GCCGACGTCGATCGGCGGCCGGC	putative amino acid permease
1358	*sav_1337*	ACCGCATGTCTTCGGC<br>GCCGAAACCATAACCCCAGGC	putative ABC transporter substrate-binding protein
1359	*sav_1342*	ACCGCAGACTGCATGTGGATCTGGC	putative transmembrane efflux protein
1360	*sav_1418*	ACCGCCATTCCCGCATCGCTCGGC	putative transporter
1361	*sav_1558*	GCCGCATGTTGCGTTCAGCC<br>GCCGCGTGTCGCC<br>ACCGATATTTCGGC	putative ABC transporter permease protein
1362	*sav_1569*	GCCGAACCTTTGCC<br>GCCGACAGGGCCAGTCCAGCC	putative ABC transporter permease protein
1363	*sav_1614*	GCCGCACGAGCCGCGACGGCCCGCC	putative benzoate transporter protein
1364	*sav_1727*	ACCGCTCCTGCC<br>ACCGCTCCTGCC<br>ACCGACGACATCGGC	putative multiple sugar ABC transporter substrate-binding protein
1365	*sav_1737*	ACCGCTCACCCCTGCC	putative multiple sugar ABC transporter substrate-binding protein
1366	*sav_1779*	ACCGCGGCGCCGCGCCCGGC<br>GCCGCGCCGGGTGCGTGCCCGGC	putative 3-(2-hydroxyphenyl) propionic acid transporter
1367	*sav_1878*	GCCGCGGACACGAAACGAGTTTGCC<br>GCCGCCTCCGCCCGGGCCGGC	putative ABC transporter ATP-binding protein
1368	*sav_1947*	GCCGACAGTTGACGTTTACCGGC	putative cytosine/uracil/thiamine/allantoin permease
1369	*sav_2066*	GCCGCAACGGCGGTTGACCTCAGCC	putative xanthine/uracil permease
1370	*sav_2092*	ACCGAAGGTTAGCGTCCGGC<br>GCCGCCGCACGACCGGACCGCC	putative multiple sugar ABC transporter substrate-binding protein
1371	*sav_2134*	GCCGACCTCCCGGC	putative ABC transporter substrate-binding protein
1372	*sav_2145*	GCCGACCGGC	putative ABC transporter permease protein
1373	*sav_2185*	GCCGAAAAACGGCGTCTTCTGCC<br>GCCGAATACCCTTCGGGTTTGGC	putative ABC transporter ATP-binding protein
1374	*sav_2187*	GCCGCCGCTCACTTGACTGCC<br>ACCGCGCCGACATCACGCCGCC	putative ABC transporter substrate-binding protein

		GCCGAGGGTCTGGC	
1375	*sav_2293*	GCCGCGGGTGCCGCGAACAGCC	putative amino acid permease
1376	*sav_2297*	ACCGCACCACCGTCACCACAGCC<br>ACCGCCCGGGGCTCCGGC	putative sugar transporter
1377	*sav_2328*	ACCGCTGACGGCCGTCCGCC	putative bldA-regulated nucleotide binding protein
1378	*sav_2365*	GCCGACGGCCAGCAGGCCGGC<br>GCCGAGTGGGTCGCC<br>GCCGCAATGTCGCTGCCGGC	putative ABC transporter ATP-binding protein
1379	*sav_2390*	GCCGACGAGTCCGGC<br>GCCGCCGCCGGACTCGTCGGC	putative amino acid permease
1380	*sav_2455*	GCCGACCGCC	putative transmembrane efflux protein
1381	*sav_2466*	ACCGATCCCGCCTCACGCTCCGGC	putative siderophore biosynthesis protein/iron transport protein
1382	*sav_2606*	GCCGCGGCCGGC<br>ACCGCAGAACCCCACCTCTCGCC	putative sulfate transporter
1383	*sav_2609*	GCCGACGACGTCAGCCGGCCGGC<br>ACCGCCTGACCGCCGGACCGGC<br>GCCGACGGCACCGGC	putative ABC transporter substrate-binding protein
1384	*sav_2613*	GCCGCGTGCTGTCCGGC<br>ACCGATCGGCCCTGCC<br>GCCGATCGGTCACTTGGC	putative ABC transporter ATP-binding protein
1385	*sav_2655*	GCCGAGCTTGCGCGTTCCGCC	putative allantoin permease-family protein
1386	*sav_2728*	GCCGCCGTGAATGGACCGCC	putative transmembrane efflux protein
1387	*sav_2790*	GCCGACGGGTGAACGGCGTCGGC<br>GCCGACGCCGTTCACCCGTCGGC	putative ABC transporter ATP-binding protein
1388	*sav_2813*	GCCGCGAGGCAGGC	putative transmembrane efflux protein
1389	*sav_2818*	GCCGCGTCGTCGTGCTTCCCGGC<br>GCCGCGTACCCCGCGGGGTCGGC	putative multiple sugar ABC transporter substrate-binding protein
1390	*sav_2860*	ACCGCGCTCATCGAGCGCACCGGC	putative ABC transporter ATP-binding protein
1391	*sav_3135*	ACCGACGACAGGTGGCACTGCC	putative ABC transporter ATP-binding protein
1392	*sav_3253*	ACCGAAGCCCCCAGCCCCCGCC<br>ACCGAAGCCCCCAGCC	putative type II secretion system protein E
1393	*sav_3321*	ACCGCCCCCTGAGGGCGGTGCCGCC<br>ACCGCCCTCAGGGGGCGGTGCCGCC	putative ABC transporter permease protein
1394	*sav_3369*	ACCGCGGTCGAGCTGGC	putative simple sugar ABC

			transporter ATP-binding protein
1395	*sav_3551*	ACCGCAGGTCGTCCGCTGACCGGC<br>ACCGACATAGGAGAGTTGCC<br>ACCGCATAAAGCCAGGC	putative ABC transporter ATP-binding protein
1396	*sav_3552*	ACCGCATAAAGCCAGGC<br>ACCGACATAGGAGAGTTGCC<br>ACCGCAGGTCGTCCGCTGACCGGC	putative integral membrane transport protein
1397	*sav_3584*	GCCGCCCGGC	putative ABC transporter ATP-binding protein
1398	*sav_3634*	GCCGACGCAGCGCCTTATCCGGC	putative ABC transporter ATP-binding protein
1399	*sav_3677*	GCCGCCCGCCTGTCGGGCAGGC	putative ABC transporter permease protein
1400	*sav_3698*	GCCGATGACCAGCC<br>GCCGAACTCATCGGC	putative transmembrane efflux protein
1401	*sav_3787*	GCCGCCCTGCC	putative sodium-coupled permease
1402	*sav_3852*	GCCGCAGGATGTCCCGGC	putative ABC transporter ATP-binding protein
1403	*sav_3872*	GCCGCCTCCCGTAATTCCGGC<br>GCCGCGGCTCGCC	putative ABC transporter ATP-binding protein
1404	*sav_3887*	GCCGACACGGCACAACCCGGC	putative transmembrane efflux protein
1405	*sav_3894*	ACCGCCCCACCTGCAACTGCC	putative integral membrane efflux protein
1406	*sav_3978*	GCCGCAGACCGCTCGCGCGCCCGGC	putative integral membrane transport protein
1407	*sav_4052*	GCCGCCCGCCGCCCGCCGCCCGCC	putative ABC transporter permease protein
1408	*sav_4053*	GCCGAGGCGGGGCCGCC	putative ABC transporter ATP-binding protein
1409	*sav_4059*	ACCGCGAGCGCCCGCC	putative ABC transporter permease protein
1410	*sav_4122*	ACCGATTGGC	putative amino acid permease
1411	*sav_4142*	ACCGATCGGC	putative ABC transporter ATP-binding protein
1412	*sav_4194*	ACCGCCTCGACCGCC	putative transmembrane efflux protein
1413	*sav_4203*	ACCGCGCTCTGCC	putative peptidoglycan-binding protein, secreted
1414	*sav_4250*	GCCGCTGCCCGCC	putative ABC transporter ATP-binding protein
1415	*sav_4280*	ACCGCGTTTGCATATAGCC	putative transmembrane efflux

			protein
1416	*sav_4439*	GCCGCAGGAGCCCCCTGTCGGC	putative permease
1417	*sav_4488*	GCCGCGTCGGCCGGC<br>GCCGCGTTGGCTTTCAGCC<br>GCCGCCGCCCGTGTCGTGTCCGGC	putative simple sugar ABC transporter substrate-binding protein
1418	*sav_4541*	ACCGCTTCCGCGTCCCGTCCGGC<br>GCCGCGTCGGC<br>GCCGCGCAGCTCGTCGAGGCAGGC	putative ABC transporter ATP-binding protein
1419	*sav_4543*	GCCGCCCGGC	putative amino acid permease
1420	*sav_4586*	ACCGACTGTAGCC<br>GCCGCGCACAGACTGGTTAGGC	putative ion-transporting ATPase
1421	*sav_4702*	GCCGACCGGC<br>GCCGCTGACCGTGCCGGCCTCTGCC	putative transmembrane efflux protein
1422	*sav_4721*	GCCGAGCCCTCCCCCGGTTCGGC	putative transmembrane sulfate transport protein
1423	*sav_4722*	GCCGAACCGGGGGAGGGCTCGGC	putative ABC transporter ATP-binding protein
1424	*sav_4769*	GCCGCTCGGCATGCCGGC<br>ACCGCCTCGATGCCCCGCTGCC<br>GCCGAAAGAGGTCTGGTGCCGGC	putative amino acid transporter
1425	*sav_4774*	ACCGCCGACTGCC<br>ACCGACAGGTCGCTACCGACAGCC<br>GCCGATTCGACCGCC	putative amino acid permease
1426	*sav_4867*	GCCGCGTCGGGCTCGGC	putative ABC transporter ATP-binding protein
1427	*sav_4894*	GCCGCGTTCATGTGCCGCC<br>GCCGACGGCTGCC	putative amino acid transporter
1428	*sav_4904*	ACCGCTTCCGCAGCC	putative transmembrane efflux protein
1429	*sav_5097*	ACCGCCGCCGGTCTCCTCCGCCGCC	putative multiple sugar ABC transporter permease protein
1430	*sav_5098*	GCCGCCCCCGTCGCC	putative multiple sugar ABC transporter substrate-binding protein
1431	*sav_5161*	ACCGAGCCGGCGGATCGCCGGC	putative proline permease
1432	*sav_5299*	ACCGCCGGCCCCGGC<br>GCCGCCGGGTCCGCC<br>GCCGCCGCCGGGGCCGGC	putative ABC transporter ATP-binding protein
1433	*sav_5344*	ACCGCCCGAACGACGTCGCC<br>GCCGCCACCGACAAGCGATGCAGCC	putative ABC transporter ATP-binding protein
1434	*sav_5367*	GCCGAGCGCCCTCGCCCGGC<br>GCCGACCGCC	putative ABC transporter ATP-binding protein
1435	*sav_5578*	GCCGCCCACCAGCC<br>ACCGCGTGCTTGATACAAATTGCC	putative transmembrane efflux protein

1436	*sav_5587*	ACCGCCAGGCACCGCC	putative transmembrane efflux protein
1437	*sav_5646*	ACCGCCGTCGCCCGCACCGCC<br>GCCGCCCGCC	putative transmembrane efflux protein
1438	*sav_5648*	GCCGCGAGGCGCCCGGC	putative efflux membrane protein
1439	*sav_5839*	GCCGCTTCCAGCC<br>ACCGCCCGCC<br>ACCGCGCTCTGCGTTCGCC<br>ACCGACCCCTGTGAGGGACGCAGGC<br>GCCGCTTCGCCAGGC	putative integral membrane transport protein
1440	*sav_5867*	GCCGCAGCCGTACATCGGC	putative transmembrane efflux protein
1441	*sav_5961*	ACCGCCGTCGCC	putative ABC transporter ATP-binding protein
1442	*sav_6040*	GCCGCGCCCGCC	putative cation/multidrug efflux protein
1443	*sav_6065*	GCCGATCCGGC	putative transporter
1444	*sav_6072*	GCCGCGCCCCCGGCCCCCGCC	putative ion transporting ATPase
1445	*sav_6162*	ACCGCTTCCCGTGACCGCC<br>ACCGCCGGTCCACGCCGCCGCC	putative integral membrane efflux protein
1446	*sav_6276*	GCCGACGCCCCGGGATCTGGC	putative integral membrane export protein
1447	*sav_6300*	GCCGCGAAGCGAGTCGGC	putative electron transport protein
1448	*sav_6303*	GCCGAGCGCACACCGGC<br>GCCGCGGCACCGGC<br>ACCGACCGGGCACCGGC<br>GCCGCCTCAGCCTCTCGGC	putative ABC transporter permease protein
1449	*sav_6322*	ACCGAGGCCGTCGGC	putative ABC transporter ATP-binding protein
1450	*sav_6353*	ACCGCGGCGCGGGTTTCCGCC	putative transport associated protein
1451	*sav_6458*	GCCGCCTCTGCGCCGCC<br>ACCGCGTACGGCTCTGCC	putative transglycosylase associated protein
1452	*sav_6480*	ACCGACCGATGGATGGGACCGCC	putative ABC transporter ATP-binding protein
1453	*sav_6527*	ACCGCCCTCTCTAGATTGCTCCGCC<br>ACCGCTCGGGTACGTCCGCCGGC	putative peptidoglycan-binding protein, secreted
1454	*sav_6528*	GCCGACCGGGTGGGACCCCGGC	putative peptidoglycan-binding protein
1455	*sav_6530*	GCCGACGGGGCCGCC	putative ABC transporter ATP-binding protein

1456	*sav_6592*		ACCGAACGGCCCGGCAGGGGCTGGC	putative integral membrane transport protein
1457	*sav_6623*		GCCGCCGGCCGTCGGC<br>GCCGAGACATCAGCC<br>GCCGACGGCCGGC<br>GCCGATGTCTCGGC	putative amino acid permease
1458	*sav_6657*		GCCGACGTTCGCC<br>GCCGCGACAGAGGTCGCCGGC	putative simple sugar ABC transporter substrate-binding protein
1459	*sav_6711*		GCCGCACCTCGGTCGCC	putative ABC transporter ATP-binding protein
1460	*sav_6812*		GCCGCCGCTCCCGCC	putative multiple sugar ABC transporter substrate-binding protein
1461	*sav_6886*		GCCGCCGGTCCCCGCCGCC<br>ACCGAGCGGGGTCCTCGCC	putative amino acid permease
1462	*sav_6900*		GCCGCTGTGCCGCC	putative integral membrane transport protein
1463	*sav_7032*		ACCGCATGTTACCCACGAGTAGCC	putative transmembrane efflux protein
1464	*sav_7043*		ACCGCGTCCACTCCCCGCCCGGC	putative cation efflux system protein
1465	*sav_7052*		ACCGCACGCGCGCGTGCGGCAGCC<br>GCCGCCGCGCCGTCCGGTCCGCC<br>ACCGCATACGCTGGC<br>GCCGCCCGCACCCCCGCC	putative amino acid transporter
1466	*sav_7234*		GCCGCTGAGCCGTCCCACCGCC<br>GCCGCCCCGCGCCCCCGCCCGGC	putative multidrug efflux protein
1467	*sav_7281*		GCCGCTCTCGCC	putative sugar ABC transporter permease protein
1468	*sav_7294*		GCCGATTGCC<br>GCCGCGTCCGGC	putative amino acid transporter protein
1469	*sav_7464*		GCCGCCTTCCCCCGTCGGC	putative transport protein
**Gas vesicle (6)**				
1470	*sav_598*	*gvpA1*	GCCGCGCCGGC<br>GCCGCCGGCGCGGCCCCGTCCGGC	putative gas vesicle synthesis protein
1471	*sav_1889*	*gvpA2*	ACCGCGTCCCCGCC	putative gas vesicle synthesis protein
1472	*sav_1887*	*gvpG2*	GCCGCCGGTGCCCGGC	putative gas vesicle synthesis protein
1473	*sav_599*	*gvpO1*	ACCGCGGCGTGCCGACTCTCGCC<br>GCCGCGGCACGGCACTGGC<br>ACCGCCGCCTCCCCTTCGCC<br>GCCGATCCATCCCGCC	putative gas vesicle synthesis protein

			GCCGCGGTGAACTGCGCTGACAGGC<br>GCCGCGGCGGGCGAGAGTCGGC	
1474	*sav_1890*	*gvpO2*	ACCGCGCAATGCGCGGAACCGGC	putative gas vesicle synthesis protein
1475	*sav_2356*	*gvpO3*	GCCGCCTGGCTGCC<br>GCCGCGCCCGCAGCC	putative gas vesicle synthesis protein
**Mobile and extrachromosomal element functions (56)**				
1476	*sav_43*		GCCGCCCTCGCCTGGC<br>GCCGAGCCCCCTGTTCGCC<br>ACCGCTGCACCTTCTGTCCCGGC<br>ACCGCGCGGGGACCAGGC	putative IS5 family IS4811-like transposase
1477	*sav_46*		GCCGATTCGCC	putative IS5 family IS1421-like transposase
1478	*sav_47*		GCCGATTCGCC	putative IS605 family IS1136A-like transposase
1479	*sav_61*		GCCGCCCACCCTCCGCCCCAGCC	putative IS605 family IS1136A0-like transposase
1480	*sav_66*		GCCGCCGACCACGACCGGC<br>GCCGACGCTGAACTACACCGGC<br>GCCGAACCCTCCTCGGC<br>GCCGAGGAGGGTTCGGCCGGC	putative IS605 family IS1136A0-like transposase
1481	*sav_78*		GCCGCGTCGGGCGGGACTTCGGC<br>GCCGCCACGGCGCCAGCC<br>ACCGCGAGCGTGATCAGCTCGGC<br>GCCGAAGTCCCGCC<br>GCCGCTTGTTGTAGCC	putative IS982 family ISCef3-like transposase
1482	*sav_103*		GCCGCGGGATCGGGGATCGGC<br>ACCGCCGGCCCGGACCCGCCGGC<br>GCCGCCGTGACCGGC<br>GCCGAAAACTGCGTGGCCGGC	putative IS256 family IS1245-like transposase
1483	*sav_118*		ACCGCCGGGCCACCTCTCCGTCGCC<br>GCCGATCCCGCTCCTCGGC<br>GCCGAGGAGCGGGATCGGC	putative IS5 family IS1647-like transposase
1484	*sav_207*		GCCGCGGACACACCGCGGATCCGGC<br>GCCGCCAACGCTCAGCGCGCAGCC<br>GCCGATGCCGCGTAGGC	putative Tn3 family transposase
1485	*sav_227*		GCCGCCTTTCGGCTCCAGCC<br>GCCGACCAGGAGTCCCCACCTGGC	putative IS701 family ISMmq4-like transposase
1486	*sav_229*		GCCGACCTACGTCGGC<br>GCCGCGACTTTCGGCACATCCGCC<br>GCCGCCTGTCCGGC<br>ACCGAAACACTCCCGCCCCGGC<br>GCCGAAAGAACACCCGGC<br>ACCGCGCAAAACACCATCGGC	putative transposase

		GCCGCAGTTGGGTGTAGGC<br>GCCGACGTAGGTCGGCTGCC	
1487	*sav_254*	GCCGAGGCGGTCGCCCCGGGCAGCC<br>GCCGCCGTATCGCC<br>ACCGCCTCGGCTCGGGTGCAGGC<br>GCCGCTGGTCCACTGGC	putative IS5 family ISFa13B-like transposase
1488	*sav_256*	ACCGACGGGGTAGGC<br>GCCGCCCTGCCCGCC<br>GCCGACCACTCCGCAGTCCTGCC	putative ISAs1 family IS1629-like transposase
1489	*sav_260*	ACCGAGTGAGTCTCCTCACCGGC	putative transposase
1490	*sav_274*	GCCGCCGCCGAGGCGTTCGTCGGC<br>GCCGACGAACGCCTCGGC<br>ACCGCCGGCGTCGCC	putative IS630 family transposase
1491	*sav_291*	GCCGAACAGCC<br>GCCGCAGCCAGGC<br>ACCGCATCTACGACGAAGCCCGCC<br>GCCGCATCTCTGCC<br>ACCGCCTGGCTGCGGCAGCC<br>ACCGCCCTGGC	putative IS30 family ISLxx5-like transposase
1492	*sav_300*	GCCGCATAGGTGCTGCC<br>ACCGACGCCCTGCCGGCAGCC	putative IS256 family IS1164-like transposase
1493	*sav_303*	GCCGCTCTTCGGGGCTGGC<br>ACCGCGGACGGACACCGGACCGGC<br>GCCGACTTCCGGAACCAACCGGC	putative IS110 family IS1547-like transposase
1494	*sav_309*	ACCGAACATCGCGTTCGTCGCC<br>GCCGAACTTGGC<br>GCCGCGGTACGCGCCACCGCC<br>GCCGAGGTCCGACACCGGC<br>GCCGATGGTAGGGGGTAGTGCCGGC	putative IS3 family ISVisp1-like transposase
1495	*sav_310*	GCCGCGACTGGC	putative IS5 family IS1373-like transposase
1496	*sav_312*	GCCGCGTTGGTCAGGTAGTCCGCC<br>ACCGAATTCCCTTGCC	putative IS4 family ISFsp5-like transposase
1497	*sav_364*	GCCGAGGCAGACAAGGGTGACCGGC	putative IS110 family ISLxx2-like transposase
1498	*sav_365*	GCCGAGGCAGACAAGGGTGACCGGC	putative IS4 family ISFsp6-like transposase
1499	*sav_375*	GCCGCTTTCGGC	putative IS605 family IS606-like transposase
1500	*sav_378*	GCCGCGTTGCC<br>GCCGCCCCGGCCCCGCAGTCAGGC	putative IS5 family ISFa13A-like transposase
1501	*sav_420*	GCCGAGAATCACTACCGACCTCGCC<br>GCCGACCCAGCC	putative Tn3 family ISXc5-like transposase
1502	*sav_423*	GCCGCCGACGAGCCCGCC	putative IS30 family

		GCCGAAGAACAGGC	ISCmi3-like transposase
1503	*sav_441*	GCCGCTGTTCCTGCCGGC	putative ISL3 family IS466A-like transposase
1504	*sav_446*	ACCGCGTAGGTTCGCC	putative IS21 family IS1474-like transposase
1505	*sav_447*	ACCGCGTAGGTTCGCC	putative IS630 family ISMma10-like transposase
1506	*sav_470*	ACCGCGTTTTCTCACAGGC	putative IS5 family IS493-like transposase
1507	*sav_473*	GCCGCGAATGAACCTGGC	putative ISL3 family IS469-like transposase
1508	*sav_509*	GCCGCACCGCC	putative IS5 family ISSco3-like transposase
1509	*sav_523*	GCCGCCGTGCACGACGAGCTCGCC<br>GCCGACGGCAGCTGGC<br>GCCGCCGCACGCAGTCCGGC<br>GCCGCCGGGGGCCCAGTGCCGCC<br>ACCGCTCAACGAGCTGCCCGGC	putative Tn3 family ISSod9-like transposase
1510	*sav_525*	GCCGAGCAGGC<br>ACCGCCAGCAAACGCACAGCC	putative IS630 family ISXo7-like transposase
1511	*sav_634*	GCCGCTTCGGGCGGTGCCGCC<br>GCCGATGCAGCCGCC<br>ACCGCGGGGCAGCC<br>GCCGCGTGCAGGGCGGCACCGCC	putative IS110 family ISLxx2-like transposase
1512	*sav_682*	GCCGATCGGC<br>GCCGCGCTTGCCGCCGATCGGC<br>GCCGCGGTCGGC	putative IS5 family ISBce20-like transposase
1513	*sav_713*	ACCGAGGCCGGC	putative IS5 family IS112-like transposase
1514	*sav_720*	GCCGCGCAGCGATGCTGTTGTCGCC	putative IS5 family IS1648-like transposase
1515	*sav_745*	GCCGAGGGGGCCCGCC<br>GCCGCATGGCGGGCCCCCTCGGC	putative IS630 family IS885-like transposase
1516	*sav_884*	GCCGCCCACAAAAGGCTCGCC	putative IS481 family ISAni1-like transposase
1517	*sav_1198*	GCCGCGCGGTTCCGACCCGCC<br>GCCGCGCGGTTGTGGCCGCC<br>GCCGCTCCGACCCGCC<br>GCCGCGCGATTCCGACCGCC<br>ACCGCCGCGCGGTTGTGGCCGCC<br>GCCGCGCCGTTCCGGCCCGCC<br>GCCGCGCGGTTGTGGCCGGCCGGC<br>ACCGCATAGGTTCGCCGGGTCGGC<br>ACCGCGAGCCGGCCGGC	putative IS607 family ISTvo1-like transposase

		ACCGCCGACCCGGC	
1518	*sav_1204*	ACCGCATAGGTTCGCCGGGTCGGC<br>ACCGCCGACCCGGC	putative IS630 family ISPsyl-like transposase
1519	*sav_1969*	GCCGCATGGATCACCGCC<br>GCCGCTGAAACGTCGCCGCC<br>GCCGAGCTCACCAGCCTGCTCGCC	putative IS110 family ISLxx2-like transposase
1520	*sav_2607*	GCCGCGGCCGGC<br>GCCGCCCACATCGAAACCGCC<br>ACCGAACGGCCCGGC	putative ISNCY family ISMk1-like transposase
1521	*sav_3722*	GCCGCATCTCGTGAACTTTGGC<br>GCCGCGTGAGGGCTGGC	putative IS701 family ISAzvi8-like transposase
1522	*sav_3741*	GCCGACCGCC<br>ACCGCTTGCCTCGGCTTGGC<br>GCCGCGCTCTCAGCAGTCAGCC	putative IS481 family ISMav2-like transposase
1523	*sav_4216*	ACCGACCGGC<br>GCCGCCGACTCCACCGCC<br>GCCGCAGCCCTCATCTGCC<br>GCCGCGCTGCTCGCGCTGCC<br>GCCGAGCCGCTTTGGCCCCGGC	putative IS5 family IS1647-like transposase
1524	*sav_5685*	GCCGCACCGCC	putative IS4 family ISMg3-like transposase
1525	*sav_7512*	ACCGCCCACCGGACCGGTGTCAGCC	putative IS110 family ISLxx2-like transposase
1526	*sav_7513*	ACCGCAAACCACCAGCC	putative IS630 family ISPsy1-like transposase
1527	*sav_7545*	ACCGAGCAGCAGGACCAGCC<br>ACCGACGAAGACGCTTGTCGGC	putative ISL3 family ISFsp1-like transposase
1528	*sav_7547*	GCCGCCCCTGGTCCACTTTTCAGGC	putative IS21 family ISMt3-like transposase
1529	*sav_7548*	GCCGACAGACGCTGGC	putative ISL3 family ISFsp1-like transposase
1530	*sav_7558*	GCCGCCCTCAGCC	putative IS5 family IS493-like transposase
1531	*sav_7570*	ACCGCGCAAAGCAGTACCTGGC<br>ACCGAGCACCGCCTCCAGCC	putative IS630 family IS885-like transposase
**Unknown or unclassified genes (1747)**			
Not listed in this table			
**Total genes: 3278**			

Underlining: conserved 5-nt inverted repeats.

Red-font numbers: SAV742 targets confirmed by EMSAs.

Shaded numbers: putative targets confirmed by EMSAs as not bound by SAV742.

Table S2. Strains and plasmids used in this study

Strain or plasmid	Description	Source or reference
Strains		
S. avermitilis		
ATCC31267	Wild-type strain (WT)	Laboratory stock
D742	*sav_742* deletion mutant	This study
C742	*sav_742* complemented strain	This study
O742	*sav_742* overexpression strain	This study
WT/pKC1139	WT strain carrying empty vector pKC1139	This study
WT/pSET152	WT strain carrying empty vector pSET152	This study
E. coli		
JM109	General cloning host for plasmid manipulation	Laboratory stock
ET12567	Methylation-deficient strain	[1]
BL21 (DE3)	Host for protein overexpression	Novagen
Plasmids		
pKC1139	Multiple-copy, temperature-sensitive *E. coli-Streptomyces* shuttle vector	[2]
pSET152	Integrative *E. coli-Streptomyces* shuttle vector	[2]
pET-28a (+)	Vector for His_6-tagged protein overexpression in *E. coli*	Novagen
pJL117	pIJ2925 derivative carrying the *Streptomyces* strong constitutive promoter *ermE*p*	[3]
pJL117-742	pJL117 carrying *sav_742* ORF	This study
pKCD742	*sav_742* deletion vector based on pKC1139	This study
pSET152-742	*sav_742* complemented vector based on pSET152	This study
pKC1139-erm-742	*sav_742* overexpression vector based on pKC1139	This study
pET-742	*sav_742* overexpression vector based on pET-28a (+)	This study

Table S3. Primers used in this study.

Primer purpose and primer	DNA sequence* (5'-3')	Use
Gene disruption, complementation and overexpression		
SD4A	CCCAAGCTTCAGATCGGCGAACACCTC (*Hin*dIII)	Deletion of *sav_742* gene
SD4B	CTAGTCTAGATTCCCACCAATAGTCGAACC (*Xba*I)	
SD5A	CTAGTCTAGAGCTGTACGTGCCGGAGTAG (*Xba*I)	
SD5B	CCGGAATTCGAGCTGATCTGCCTGGACTT (*Eco*RI)	
SD24A	ATCGGAACCGACGACAAC	Confirmation of *sav_742* deletion in D742
SD24B	GCGGTTGTGATGTGATGAGT	
SD35A	CCCGAGACGTACCACCTGA	
SD35B	AGGTAGCTGAGGGATATGTGA	
SD22D	TGCTCTAGACACTCGTTCGCAACACAC (*Xba*I)	Complementation of D742
SD22B	CCCAAGCTTTGACCTGTTCACCCAGCGAT (*Hin*dIII)	
SD22C	TGCTCTAGATCCGCGTGGAAGGATCTC (*Xba*I)	Overexpression of *sav_742* in *S. avermitilis*
SD22B	CCCAAGCTTTGACCTGTTCACCCAGCGAT (*Hin*dIII)	
SD22A	CGCGGATCCATGAACGGAATGATCGGGAGC (*Bam*HI)	Overexpression of His_6-SAV742 protein in *E. coli*
SD22B	CCCAAGCTTTGACCTGTTCACCCAGCGAT (*Hin*dIII)	
5'-RACE		
Oligo (dT) anchor primer	GACCACGCGTATCGATGCGCACTTTTTTTTTTTTTTTv	
Anchor primer	GACCACGCGTATCGATGTCGAC	
SD165A	CTTCCAGAACGGCGAGCCTG	Identification of TSS of *sav_742*
SD165B	TCGGACGGCTGGAGAGTG	
SD165D	CAGAGCACCGAGGCCCTC	
DNase I footprinting assay		
FAM-SD169A	GTGTCCCGCCTGCTCTCC	*sav_742* promoter region
SD169C	GCCGATCAGTTCCCACCAATAG	
EMSA		
SD73A	TGCTCACCACCAACTGACC	Probe *sav_742p*
SD73B	CCCGATCATTCCGTTCATC	
SD144A	GGCATCGCCTTCTTCAAGTA	Probe I
SD144B	CGCAGATCGTTCTTCCAGTC	
SD171A	CGGCCGAACGCAAAATCGCTCGGCCGGCCGAGCGGACGCACTCGGCTTGC	Probe P1
SD171B	GCAAGCCGAGTGCGTCCGCTCGGCCGGCCGAGCGATTTTGCGTTCGGCCG	
SD178A	CGTCTAGACGCAAAATCGCCATGGCGAGATCTCGGACGCACATATGTTGC	Probe P1m
SD178B	GCAACATATGTGCGTCCGAGATCTCGCCATGGCGATTTTGCGTCTAGACG	
aveA1p-Fw	ATGGTCGGGAACCTCCGCAA	Probe *aveA1_aveD*_int

aveA1p-Rev	CTGTGTCCTCACCGCTAGGC	
SD111A	CGACAAGAGAAATCGGAAATT	Probe *aveA4_orf1_* int
SD111B	GCCTGCACCTGTGACAAG	
aveFp-Fw	GCAGCGTCGCGTGAACTC	Probe *aveFp*
aveFp-Rev	GCCCCAGGCAGAACGGAC	
aveRp-Fw	CCGCACCGCCATACATAC	Probe *aveRp*
aveRp-Rev	GAAACTCCCTGCATGATGTTC	
SD74A	CGCAAGATCTATGTCGACCTCAC	Probe *sig8p*
SD74B	CTCTTGTTGCCTCGGTCACTGT	
ZJY19	GCCACACGCACCCTCCG	Probe *avaR1p*
ZJY20	GGGAGTTGGGGGTGAGATATGTG	
SD208A	AGCCATACGACTCTTCTCTGTG	Probe *sig25_smrA_*int
SD208B	GGTATCCCTGCGGTCTTG	
olmRIp-Fw	CCGCACCCTCCCGACCTG	Probe *olmRIp*
olmRIp-Rev	GACCTCGCCCAATCAGCACC	
SD327A	GGTTCTACGGGGTCAAGGAG	Probe *olmA1p*
SD327B	CTATCGGCTGAATGGATCGC	
SD182A	GGCCTGGTGAATTCCTGC	Probe *olmA4p*
SD182B	GAAGGTACGGACGGCTCG	
SD186A	CCTGGAGCGGTGAGGGCA	Probe *melC1p*
SD186B	GCGGGTGAGTTCGGGCAT	
SD187A	CCTCAACCACCAGCGAAAC	Probe *melC1-2p*
SD187B	CCCCAGCGCGAACAGCAC	
SD320A	GGTCTCACACCGTCCTCTA	Probe *pteF_pteR_*int
SD320B	GGTATCGCCAGATGTTGAA	
SD321A	TCCCGACTGTCAATGCTC	Probe *pteA1p*
SD321B	AGAGAACGAAAGGCACCG	
SD322A	GGCCTTGTCTGAACGCATTC	Probe *crtUp*
SD322B	CGCCTTGTCGGAAGCATT	
SD323A	CATCACCCACACCGTCGT	Probe *crtY_crtE_*int
SD323B	GGTCGTGCTCCGTTGAAC	
SD243A	GTCCTAAACGAGCCATCTACC	Probe *pks1-3p*
SD243B	GCATCGGATTCCTGTGTG	
SD318A	ACACCCATGCTTTGTCCGAG	Probe *pks3-2p*
SD318B	CCGGTTGTAGGTGAGGATGG	
SD211A	CCTCGCCGTTGATCTCACC	Probe *amfCp*
SD211B	CCCTCGACCGGATTGTGG	
SD210A	CCGATGGCTGTAGGCGTTTAT	Probe *whiBp*
SD210B	CCCTCGTCTGTCTTCGCG	
SD194A	GAGCCCCACGATGAAAAC	Probe *ssgCp*
SD194B	CGACCATTCCGGTGACTG	
SD221A	CATCGCGCAGGGAAACGG	Probe *amfRp*
SD221B	CGGGGTAAAGGGGGTCTC	
SD115A	GCCCGGTTCTCGTAAGAC	Probe *ftsHp*

SD115B	CAGCACGATCCACATGAC	
SD218A	GCACTGCTACGGCTGATC	Probe *ftsZp*
SD218B	TCGCCTCGAGTTACGTGT	
SD276A	CATTGGCCGTAGTCCACAC	Probe *aceE1p*
SD276B	ATCGAGACGTCCGTACAGAC	
SD277A	TCAGGACTCGTACGGAACAC	Probe *acnAp*
SD277B	GGTCACCCTTACCAAGCTG	
SD278A	GTCCATTGAGCACCAGAACC	Probe *korAp*
SD278B	CCTGTTCCGTTGTACTGCTG	
SD279A	TTGGTCCTCGCGCTGTAC	Probe *icdAp*
SD279B	ATCTCGTGATGGGGTCCC	
SD284A	CCCAACGCTGGTCCTACT	Probe *pgmAp*
SD284B	GACAGTTCGGCTTCATCG	
SD283A	GGCAGTTGGTGTCGTGTAA	Probe *gap2p*
SD283B	CCTACCTCCCTGAAGCCC	
SD285A	CCCCTTTGTATCTTCCACCAGA	Probe *pykA1p*
SD285B	GGGCCACGTTCATGCCGG	
SD196A	CGGGACCTACATATGCAAG	Probe *dasRp*
SD196B	CGGTAGTACTTGGGCACAC	
SD293A	AGACCTTGACCACATCGG	Probe *accA3p*
SD293B	ATGACTCCCTCTCCTTGAAAC	
SD226A	CCCGGCGGTCTGTTCCTC	Probe *accD4p*
SD226B	TCCTGCTCGAGATCCTGCTGAG	
SD290A	CCACTCGCCCGTCCTGAT	Probe *fadD5*
SD290B	CTGCTCCTTCGTCCGCCT	
SD291A	ATGGTGGGTCCAGATTTCCTAC	Probe *fadA6*
SD291B	GAGCAGTTCGACGGGGTG	
SD292A	CCTTGTGGTTCGTGGCTT	Probe *pcaF1*
SD292B	CGACCTGACTCCTGACCG	
SD274A	CGGTCCCGCATACTGATACG	Probe *leuA2p*
SD274B	CGGTACTTGTGGATCGGCAT	
SD281A	GCCGCTACTGTTGTCCAT	Probe *leuCp*
SD281B	TCGTCCCAGACCTTCTCC	
SD280A	GCCCTCCGCTCACATAAT	Probe *ilvB1p*
SD280B	GTGTTGAACTGGTGCCTGT	
SD272A	GAGGCCATGCTCAAGATGC	Probe *ilvCp*
SD272B	TGGGTTCTCTCCTTGGTGTG	
SD273A	CGGAACAGATCATCGCCAG	Probe *ilvD1p*
SD273B	GCGGTGCCTACGTCTCGG	
SD275A	GTACGAGTAGCAGGCTGACC	Probe *ilvEp*
SD275B	CTGTGAACGGTGTGCGTG	
SD179A	GGCGGCCATTACGGATAC	Probe *glnA1p*
SD179B	CCACCACAACACCATTTCG	
SD288A	CAGGACGACATGGACCCC	Probe *ndh1p*

SD288B	GTCACCCAAACCCGACGG	
SD286A	GGTTCCGTATGCGTTGCG	Probe *ctaD2p*
SD286B	ATGCGGTGTCTGCTTCGG	
qRT-PCR		
16S-QP-Fw	AGCGGAGCATGTGGCTTAAT	*16S rRNA* ORF
16S-QP-Rev	ACGTATTCACCGCAGCAATG	
SD66A	GGGGATCGGGGATGAAC	*sav_742* ORF
SD66B	ACCAATAGTCGAACCGGTTG	
SD65A	TTGTCCCGGCTGTTCTTC	*sig8* ORF
SD65B	GACGAGGGTGACGTTCATCT	
GJ99	CGGACAGGACTACGCACTTC	*aveA1* ORF
GJ100	ACGAGATACGACCGGAGATC	
aveD-QP-Fw	GGACTACTACGACCGTTTGACC	*aveD* ORF
aveD-QP-Rev	CTCAGCTTGCCGATGAGGAG	
SD161A	TGGAGGTGTCCTGGGAGG	*aveA4* ORF
SD161B	ACCGAGAGCAGACGGGTC	
SD120A	GGACCTGCTGGTGAACAAC	*orf1* ORF
SD120B	CGGCAACCATGAATGACAC	
aveF-QP-Fw	CCGCCACCGACATGAACC	*aveF* ORF
aveF-QP-Rev	GGTGACCCAACGGCCTTC	
GJ97	CAGAAGAACTCACGCTCGTC	*aveR* ORF
GJ98	ACTCTTTCCACAGCCCATTC	
ZJY62	ATCCGATCGTCCAGGGCAG	*avaR1* ORF
ZJY63	GAAGAGGGACTGCGTGTGC	
LS28	CTGCAGAGCGCGCTGTTC	*sig25* ORF
LS29	GGTTGGTCATGGTGCGACG	
LS30	ACTATGTCGTGAAGCCCGTG	*smrA* ORF
LS31	TCCTCGCCGTTCTTGGTGA	
SD324A	GAGTTCTTCGGCGTCTCC	*olmA4* ORF
SD324B	GGTGTTCTCCAGGGCTTC	
SD325A	CACTCTCTTGCTGATTCCG	*pteF* ORF
SD325B	CCGTTGAACTGCCTGAAG	
SD326A	TGCCCACCGAACTACTTGTC	*pteR* ORF
SD326B	GCACCGAAATTTGGCCTGTT	
SD250A	CGTGCTCGACCTCACCTC	*pks1-3* ORF
SD250B	CGGGAAAGGCATCGTTGT	
SD305A	CGAGGTGTACGACGACTTCC	*pks3-2* ORF
SD305B	TGACGTTGGAGATCAGGCTG	
SD219A	GCCGACCTCAGCTATGTG	*amfC* ORF
SD219B	GAAAGCCGCTCCATGAC	
SD203A	GACAGCGGCAGTGACAAG	*whiB* ORF
SD203B	TTGGCAGCCTCGAACTTC	
SD199A	GTGGCATGTCGATTTCGC	*ssgC* ORF
SD199B	GGGTACAGGCAGTGAGGACTC	

SD309A	GGAGTTCCCGACCGTCTC	*aceE1* ORF
SD309B	CGTGCGACTTGGAGGTGT	
SD310A	AGCCTGAAGGTGCTGCTG	*acnA* ORF
SD310B	GAGTCCCAGTTGCCGATG	
SD311A	TCACCTCAGAGACGGCGT	*korA* ORF
SD311B	GGGTCAGGATGTCGTGGT	
SD318A	ACACCCATGCTTTGTCCGAG	*pgmA* ORF
SD318B	CCGGTTGTAGGTGAGGATGG	
SD200A	ATGAGCACCGACGTCAGC	*dasR* ORF
SD200B	GCAGCGTCTCCGTCATGT	
SD249A	CTTCGGCATGGAGAACAACC	*accD4* ORF
SD249B	AAGTCCTGCGAGAACACGG	
SD316A	GGTGTCGAGTCCATGAGCC	*fadA6* ORF
SD316B	ACTTGGCGGCGATCAGTTC	
SD317A	AGAACGGGTACTTCGACGAC	*pcaF1* ORF
SD317B	CTTGAGACGGGACAGCTTCT	
SD306A	GGGCTACAAGGAGATCGAGG	*leuA2* ORF
SD306B	CGGAGATCGTGACGTCGTC	
SD308A	ATGGGTAGGACACTCGCGG	*leuC* ORF
SD308B	ATGAAGAGGAGGTCGGGCT	
SD307A	CGGAGACGGTATTCGGTATC	*ilvB1* ORF
SD307B	GCGCTTCGAGTCCATCAG	
SD313A	CCGACCTGTCCATCATCCAG	*ilvC* ORF
SD313B	CCCGAGTCACGCAGCGAC	
SD188A	GGACAAGCAGCAGGAGTTC	*glnA1* ORF
SD188B	TTGAGGAAGCCGAGTACG	
SD315A	AGTTGGGTCTGGTGCGGT	*ndh1* ORF
SD315B	AGTGGTGGCGAGCCTCTC	
SD312A	CCTCTATCTGATCACGTCGTTC	*ctaD2* ORF
SD312B	GTTGTCCACGATCTGCATG	

* Underlining: restriction endonuclease site; corresponding enzyme is shown in parentheses at the end of the sequence.

References

1 Macneil, D. J. & Klapko, L. M. Transformation of *Streptomyces avermitilis* by plasmid DNA. *J Ind Microbiol Biotechnol* **2**, 209-218 (1987).

2 Bierman, M. *et al.* Plasmid cloning vectors for the conjugal transfer of DNA from *Escherichia coli* to *Streptomyces* spp. *Gene* **116**, 43-49 (1992).

3 Li, L. *et al.* Overexpression of ribosome recycling factor causes increased production of avermectin in *Streptomyces avermitilis* strains. *J Ind Microbiol Biotechnol* **37**, 673-679 (2010).
